# Computational Study of the Photophysical Properties and Electronic Structure of Gold (III) Complexes with Different Groups

**DOI:** 10.1002/open.70248

**Published:** 2026-07-13

**Authors:** Caijie Bu, Tao Yuan, Han Xiao, Jing Wei, Minyi Zhang

**Affiliations:** ^1^ College of Chemistry and Materials Science Fujian Normal University Fuzhou Fujian P. R. China; ^2^ State Key Laboratory of Structural Chemistry Fujian Institute of Research on the Structure of Matter Chinese Academy of Sciences Fuzhou Fujian P. R. China; ^3^ Fujian College University of Chinese Academy of Sciences Fuzhou Fujian P. R. China

**Keywords:** electronic structure, luminescence mechanism, luminescent materials, photophysical properties, transition characteristics

## Abstract

A series of novel delayed phosphorescent gold(III) complexes, Au(EDG)(EAG) (where EDG = electron‐donating group and EAG = electron‐accepting group), have been reported, exhibiting stable and high‐performance luminescence. The thermally stimulated delayed phosphorescence (TSDP) of these gold(III) complexes has been attributed to phosphorescence emission from the second triplet excited state T_2_. However, the underlying reason why the T_2_ state dominates the phosphorescent emitting state remains unclear. Moreover, the effects of different substituent groups on TSDP efficiency have not been fully explored. Thus, this work employs time‐dependent density functional theory (TD‐DFT) calculations to investigate the electronic structures, transition characteristics, and photophysical properties of Au1‐7 complexes, aiming to elucidate the TSDP luminescence mechanism of these complexes. Analysis of electron–hole orbitals reveals that the internal conversion (IC) from T_1_ to T_2_ is more feasible. We evaluated intersystem crossing (ISC) rates and Huang–Rhys factors to rationalize the experimental observations. Our results indicate that the introduction of electron‐accepting group substituents, such as phenylmethyl group, significantly alters the transition properties of the T_1_ state. The nonradiative process from T_1_ to T_2_ is enhanced, leading to an extended triplet state lifetime. These findings provide valuable insights for the rational design of next‐generation luminescent materials based on gold(III) complexes.

## Graphical abstract

This work presents a theoretical investigation of seven gold(III) complexes, focusing on the influence of T_1_ and T_2_ states transition characteristics on internal conversion processes and their implications for the efficiency of Thermally Stimulated Delayed Phosphorescence (TSDP). The ligand‐to‐ligand charge transfer (LLCT) transition feature of T_1_ is highly sensitive to the environment, which is more prone to internal conversion to the T_2_ state with the internal ligand charge transfer (ILCT) transition. Huang–Rhys factor analysis indicates the vibrational normal mode predominantly of T_1_ associated with the most intense coupling corresponds to the vibration of the EAG (phenylmethyl group), the nonradiative process of Au1 ˜ 3 complexes from T_1_ to T_2_ is more favorable than other Au complexes. EDG (carbazolate group) and EAG (phenylmethyl group) arise to the extension of the conjugated system as designed Au5, which is significantly enhanced in the visible light absorption capacity.

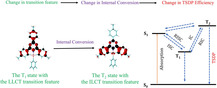

## 1 Introduction

Phosphorescent organic light‐emitting devices (PHOLEDs) have attracted increasing attention over the past few decades due to their widespread applications in high‐resolution mobile phones, tablet displays, and lighting. The development of stable and high‐performance phosphorescent emitters is of critical importance [1, 2, 3]. Among various candidate materials, Au(III) complexes have emerged as promising candidates due to their high chemical stability and longer triplet‐state lifetime. These properties endow Au(III) complexes with exceptional photochemical and photophysical properties, particularly in energy and electron transfer processes, making them a focal point of extensive research. Their phosphorescent characteristics originate from the interplay between molecular structure and electronic configuration, enabling the design of phosphorescent materials that span the entire visible spectrum. However, the relatively strong nonradiative processes, such as intersystem crossing, can lead to significant energy dissipation during the transition. This poses a major challenge in achieving efficient and stable triplet‐state emission, limiting the overall performance of PHOLEDs [4, 5, 6].

**SCHEME 1:**
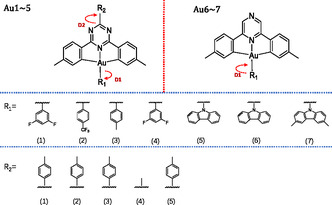
Overview of the structure of complexes Au1‐Au7. The *D*1 and *D*2 represent dihedral angles, *R*_1_= electron‐donating group, *R*_2_ = electron‐accepting group.

Recently, Vivian Wing‐Wah Yam and coworkers designed a series of functional Au(EDG)(EAG) complexes (where EDG = electron‐donating group and EAG = electron‐accepting group), as shown in Scheme 1. The Au1‐Au4 complexes feature a tridentate EAG and a phenyl EDG coordinated to the Au(III) center. Their photophysical performance follows the order Au1‐Au3 > Au4, primarily due to the differences in the internal conversion (IC) efficiency between triplet states and in the extent of visible light absorption. The Au4 complex, which lacks a phenylmethyl substituent in the triazine‐based EAG, exhibits weaker photochemical and photophysical properties. The Au6‐Au7 complexes feature diazine‐based EAG andcarbazolate‐based EDG coordinated to the Au(III) center. It was found that when the carbazolate group acts as the EDG group, it induces a red shift and shortens the triplet state lifetime. To explore the intrinsic structure‐property relationships among these molecules, they designed a new Au5 complex featuring a triazine‐based EAG and an acarbazolate‐based EDG. Remarkably, they discovered, for the first time, a novel emission mechanism called thermally stimulated delayed phosphorescence (TSDP). In this process, thermal energy facilitates thespin‐allowed IC from the lowest triplet state T_1_ to the second lowest triplet state T_2_. This thermal activation leads to emission from the higher‐energy T_2_ state, which is crucial for achieving stable, high‐energy phosphorescent emitters across various colors and promotes the development of new types of PHOLEDs. Thus, the study of auxiliary ligands in Au1−7 complexes plays a vital role in enhancing the photophysical properties of gold complexes [2, 7]. These findings provide an opportunity to further elucidate how ligand engineering improves the performance of these complexes as luminescent materials.

In these Au(III) complexes, two distinct triplet states have been identified, both of which play critical roles in determining the overall photophysical and photochemical properties. However, the nonradiative processes involving the singlet states and the two distinct triplet states in these compounds remain poorly understood, and the microscopic electronic mechanism underlying such complex systems is still unclear. Processes involving different spin multiplicities are crucial for improving key properties of Au(III) complexes, including visible light absorption capacity and excited‐state lifetimes. Through theoretical calculations, the TSDP emission mechanism can be further elucidated, and the influence of ligand functionalization on TSDP efficiency can be systematically investigated, thereby clarifying its impact on the luminescent performance of these complexes.

This study presents theoretical investigation of nonradiative processes involving different spin multiplicities, providing detailed insights into the photochemical relaxation pathways of Au(III) complexes. A comparative analysis of the geometries and excited states of Au1−7 complexes reveals changes in electronic and geometric configurations upon photoexcitation. Wavefunction analysis further provides detailed insights into their coordination structures and transition characteristics [8, 9]. The absorption and charge–transfer spectra are examined to assess visible light absorption capability and the nature of electronic excitations. By considering spin–orbit coupling and electronic orbital coupling, the rates of intersystem crossing (ISC) and IC are calculated using semiclassical Marcus theory, and the Huang–Rhys factors are evaluated to quantify the efficiency of nonradiative processes such as IC. These theoretical calculations successfully rationalize the experimentally observed differences in triplet state lifetimes. Notably, our results indicate that a triazine‐based EAG plays a key role in determining the performance of phosphorescent materials. Furthermore, this study elucidates the TSDP mechanism from a microscopic electronic perspective and explores the relationship between different ligand substituents and their luminescent performance. For example, in the case of the EAG, the phenylmethyl group is essential for the transition characteristics of T_1_ state, a feature that has not yet been addressed experimentally before. Notably, the newly designed Au5 complex exhibits strong absorption in the visible region and shows a high internal crossing efficiency compared to other gold complexes, indicating its potential as a thermally activated TSDP emitter.

## 2 Methodology

### 2.1 Theoretical Backgrounds

Nonradiative transitions are crucial for dissipating electronic energy in both singlet (Sn) and triplet (Tn) excited states, thereby contributing significantly to molecular photostability and the photophysical properties of luminescent materials. Typical nonradiative processes include IC and ISC, both of which strongly influence photochemical properties. The rates of nonradiative transitions are primarily calculated using Fermi's Golden Rule [10, 11, 12].

Equation (1) is based on Fermi's Golden Rule derived from time‐dependent perturbation theory; *ħ* denotes the reduced Planck constant; $\Psi_{i}$ and $\Psi_{f}$ represent the vibrational wavefunctions of the initial and final states, respectively; and $\delta (E_{i}-E_{f})$ is the Dirac delta function ensuring energy conservation. In practical calculations, the harmonic oscillator approximation and the Franck–Condon approximation are commonly employed to evaluate the vibronic contributions to nonradiative transitions. For general electron transfer reactions where the spin multiplicity remains unchanged, $\left\langle \Psi_{i}|H_{\mathrm{if}}^{\prime }|\Psi_{f}\right\rangle$ represents the electronic coupling matrix element. For processes involving a change in spin multiplicity, such as intersystem crossing, $\left\langle \Psi_{i}|H_{\mathrm{if}}^{\prime }|\Psi_{f}\right\rangle$ denotes the spin–orbit coupling matrix element [13, 14].

(1) $$k_{i\to f}=\frac{2\pi }{\hbar }|\left\langle \Psi_{i}\left|H_{\mathrm{if}}^{\prime }\right|\Psi_{f}\right\rangle {|}^{2}\delta \left(E_{i}-E_{f}\right)$$

The spin–orbit coupling (SOC) between the singlet and triplet states is described by the matrix element $\left\langle \Psi_{S}|H_{\mathrm{soc}}^{\prime }|\Psi_{T}\right\rangle$, as shown in Equation (2). Here, $\Psi_{S}$ and $\Psi_{T}$ represent the wave functions of the singlet and triplet states, respectively, and $H_{\mathrm{soc}}^{\prime }$ denotes the SOC operator. Both one‐electron and two‐electron SOC integrals are considered in $H_{\mathrm{soc}}^{\prime }$ [15].

(2) $$\left\langle \Psi_{S}\left|H_{\mathrm{soc}}^{\prime }\right|\Psi_{T}\right\rangle =\langle \Psi_{S}|H_{\mathrm{soc}1}^{\prime }+H_{\mathrm{soc}2}^{\prime }|\Psi_{T}\rangle$$

To quantify the vibronic coupling effects associated with nonradiative transitions, the normal vibrational coordinates of the initial and final electronic states are related through the Duschinsky transformation, as expressed in Equation (3) [16]. In this equation, *J* denotes the Duschinsky matrix, and ΔQ represents the displacement vector between the equilibrium geometries of the two electronic states. The displacement vector Δ*Q* obtained from the Duschinsky transformation provides the basis for evaluating the reorganization energy associated with each vibrational mode. Under the harmonic oscillator approximation, where λ_*i*_ represents the reorganization energy of the *i*‐th vibrational mode, k_*i*_ denotes the corresponding vibrational force constant, and $\Delta Q_{i}$ is the displacement along the normal coordinate. Based on these quantities, the Huang–Rhys factor $s_{i}$ can be calculated according to Equation (5), where h is Planck's constant, and ${\text{ν}}_{i}$ is the vibrational frequency of the *i*‐th normal mode. The Huang–Rhys factor is used to characterize the strength of vibronic coupling and evaluate the contribution of each vibrational mode to nonradiative transitions [17, 18, 19].

(3) $$Q_{2}=JQ_{1}+\Delta Q$$

(4) $$\lambda_{i}=\frac{1}{2}k_{i}\;\Delta Q_{i}^{2}$$

(5) $$s_{i}=\frac{\lambda_{i}}{h{\text{ν}}_{i}}$$

In phosphorescent materials, phosphorescence emission primarily originates from the radiative transition from triplet excited state to the singlet ground state. When SOC is considered, the phosphorescence transition dipole moment (*μ*) and oscillator strength (*f*) are evaluated using quadratic response theory [20, 21], while the phosphorescence rate is calculated using Einstein's spontaneous emission equations [22, 23, 24] (Equations (6)–(8)).

(6) $$\mu_{T_{1}\to S_{0}}=\sum_{k}\frac{\left\langle \psi_{T_{1}}|H_{\mathrm{SOC}}^{\prime }|\psi_{S_{k}}\right\rangle }{E_{T_{1}}-E_{S_{k}}}\mu_{S_{k}\to S_{0}}+\sum_{m}\frac{\left\langle \psi_{T_{m}}|H_{\mathrm{SOC}}^{\prime }|\psi_{S_{0}}\right\rangle }{E_{T_{m}}-E_{S_{0}}}\mu_{T_{m}\to T_{1}}$$

(7) $$\begin{array}{l} f_{T_{1}\to S_{0}}=\frac{2}{3}\left(E_{T_{1}}-E_{S_{0}}\right){\left|\mu_{T_{1}\to S_{0}}\right|}^{2} \end{array}$$

(8) $$\begin{array}{l} k_{p}=\frac{2}{c^{3}\hbar^{4}}f_{T_{1}\to S_{0}}{\left(E_{T_{1}}-E_{S_{0}}\right)}^{2}=\frac{f_{T_{1}\to S_{0}}{\left(E_{T_{1}}-E_{S_{0}}\right)}^{2}}{1.4992} \end{array}(f/a.u,E/{\mathrm{cm}}^{-1})$$

### 2.2 Calculation Details

The geometric structures of the complexes were adopted from previous studies by Vivian Wing‐Wah Yam [7, 25], in which representative Au complexes were reported. The tert‐butyl groups, which have negligible impact on the present study, were replaced by methyl groups to reduce the computational cost. Geometric optimizations of Au1‐Au7 complexes were carried out using Gaussian16 program (revision C.01) [26]. DFT calculations for the ground state (S_0_) and the lowest triplet T_1_ state were performed using the PBE0 exchange‐correlation functional and the def2‐SVP basis set [27, 28, 29]. Higher excited states (S_1_ and T_2_) were investigated within the framework of time‐dependent density functional theory (TD‐DFT). The solvent environment was simulated using the Integral Equation Formalism Polarizable Continuum Model (IEF‐PCM) [30, 31, 32] with water selected as the solvent. Grimme's third‐generation dispersion correction (D3) was employed for dispersion interactions [33, 34]. The electronic coupling matrix elements and SOC (Spin‐Orbit Coupling) matrix elements were calculated at the CAM‐B3LYP/def2‐SVP theoretical level [35] using the Dalton2022 program [36, 37]. Single‐electron and two‐electron SOC integrals between the excited state S_1_ and triplet states (T_1_ and T_2_), as well as the electronic coupling integrals between T_1_ and T_2_, were computed. Additionally, the Multiwfn program (version 3.8) was used for wavefunction analysis [38, 39], including the hole‐electron [40] and odd‐electron density (OED) analysis [41]. Charge decomposition analysis (CDA) [42] and the newly proposed charge–transfer spectra (CTS) analysis [9] were also performed using Multiwfn. Visual Molecular Dynamics (VMD, version 1.9.3) was employed to calculate the root–mean‐square deviation (RMSD) of the structures and for visualization [43, 44].

## 3 Results and Discussion

### 3.1 Geometric Structure

The optimized structures of all ground‐state and excited‐state gold complexes are provided in Section 2.5 of the Supporting Information. Table 1 and Figure S1 show that the calculation of minimum RMSD values enables a quantitative comparison of structural changes between the ground state S_0_ and various excited states, which are crucial for the photochemical processes of luminescent materials. These minimum RMSD values reflect the extent of structural changes associated with absorption or emission processes, in accordance with the Franck‐Condon principle [45]. Overall, the structural deformations of T_1_ or T_2_ triplet states in all the gold complexes are lower relative to their S_0_ states, indicating that the structural changes upon triplet excitation are minimal. As a result, the geometric structure of these gold complexes remains relatively stable as the electron is excited to their triplet states. Consequently, these triplet states are less likely to undergo reverse intersystem crossing to S_1_ and subsequently relax back to the ground state.

**TABLE 1:** Comparisons of ground‐state and excited‐state structure of Au1‐Au7 by minimizing the molecular RMSD values.

RMSD	Au1	Au2	Au3	Au4	Au5	Au6	Au7
S_0_/S_1_	0.504	0.509	0.431	0.435	0.221	0.288	0.474
S_0_/T_1_	0.131	0.131	0.133	0.213	0.212	0.251	0.482
S_0_/T_2_	0.190	0.133	0.183	0.217	0.081	0.192	0.466

Although RMSD analysis provides valuable insights into the overall structural dynamics, it is essential to complement it with additional analyses. Table S1 lists the computed geometric parameters for the four studied states (S_0_, S_1_, T_1,_ and T_2_) of Au1−7 complexes. Since our work mainly focuses on the influences of EDG and EAG introduction, we discuss in detail only the changes in the dihedral angles (see Scheme 1, *D*1 and *D*2) associated with the ligand groups.

The changes in the *D*1 value between T_1_/T_2_ and S_0_ states in all gold complexes do not exceed 2°, whereas considerably larger structural distortions are observed between the S_1_ and S_0_ states, reaching over 20° in several complexes. As a result, T_1_ and T_2_ served as competing photochemical pathways, and it is more likely that these triplet states will return directly to the ground states through phosphorescence emission. Moreover, *D*2 values also exhibit a trend similar to that of D1, indicating the EAG(phenylmethyl group) is also important for the photochemical process of the Au1‐Au3 and Au5 complexes. Notably, for the Au5−7 complexes, where the EDG is a pyridyl group, the RMSD values of the S_1_ and T_1_ states relative to S_0_ are highly similar. The changes in *D*1 for Au5−7 in the S_1_/T_1_ state relative to the S_0_ state (less than 10°) are the main reason for the similar RMSD values, reflecting the coplanarity of the pyridyl group. Overall, ligand modification of gold complexes can modulate the extent of aromatic ring conjugation, thereby influencing the electronic transition modes and photophysical behavior.

### 3.2 Transition Feature

**FIGURE 1:**
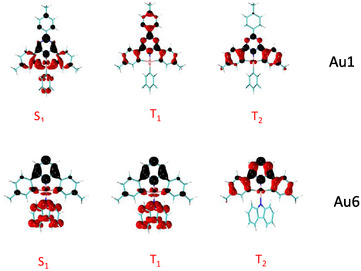
The real‐space distribution (S_1_, T_1_ and T_2_ states) of holes and electrons in Au1 and Au6 complexes. The real‐space distribution of holes and electrons in rest of the molecules is provided in Figure S2. The red and black isosurfaces represent the hole and electron distributions, respectively.

In this section, we elucidate why these gold complexes are more likely to emit phosphorescence from the T_2_ state. Electron‐hole analysis was employed to investigate the transition characteristics of these gold complexes. Compared to natural transition orbitals (NTO), electron–hole analysis provides a more powerful and practical approach for comprehensively examining the transition features of electronic excitations [40]. The real‐space distributions of electron–hole pairs for all excited states of the gold complexes are presented in Figures 1, S2, and S3 and Table S2. For the S_1_ excited state of all gold complexes, electrons are primarily distributed on the EAG (pyrazine group), while holes are mainly located on the EDG (substituted phenyl group). Thus, the electrons and holes in the S_1_ excited state exhibit limited spatial overlap and broad separation, which indicates a typical feature of ligand‐to‐ligand charge transfer (LLCT) from the EDG to the EAG.

When the EAG contains a phenylmethyl group, as in Au1−3 complexes, the electron–hole distributions in the T_1_ excited state undergo significant changes, with holes predominantly localized on the EAG. This results in the LLCT transition characteristics from EAG to EAG. In contrast, the Au 5–7 complexes retain LLCT transitions from the EDG to the EAG in the T_1_ excited state, similar to those observed in the S_1_ state. In T_2_ excited states, both electrons and holes are both mainly distributed on the pyrazine group of EAG, which presents the characteristic of internal ligand charge transfer (ILCT). But the Au5 complex, containing an EAG and an EDG, exhibits LLCT transition feature in the T_2_ state, which arises from the conjugation effect of the carbazolate group. In addition, all gold complexes exhibit the distribution of electrons and holes on the metal surface across their various excited states, which can be attributed to the metaltoligand charge transfer (MLCT) characteristics by metal perturbation. These transition features are significant for identifying the bands in absorption spectra. Notably, since the LLCT transition mode is highly sensitive to the environmental effects, the T_1_ state is more prone to internal conversion to the T_2_ state. For instance, the Au4 complex, which lacks the phenylmethyl group, exhibits identical ILCT transition characteristics in both the T_1_ and T_2_ state. As a result, the TSDP efficiency of Au4 complex is lower than that of Au1−3 complexes.

### 3.3 Photophysical Properties

Experimental spectral studies indicate that the observed phosphorescence lifetimes in these gold complexes arise from contributions of both T_1_ and T_2_ triplet states, which exhibit distinct lifetimes, suggesting a thermal equilibrium between the two triplet states.

Potential competing photochemical pathways involve the T_1_ state with LLCT transition feature and the T_2_ state with ILCT transition feature [22, 23]. The rates of possible nonradiative processes were calculated, including ISC from S_1_ to T_1_ or T_2_, IC from T_1_ to T_2_, as well as the corresponding reverse processes, namely reverse intersystem crossing R_ISC_ and reverse internal conversion R_IC_. The nonradiative processes between T_1_ and T_2_ states can be treated as a first‐order equilibrium reaction, characterized by equilibrium constant *K* (*K* = *k*_IC_/*k*_RIC_). It should be emphasized that this equilibrium constant *K* does not imply that a true thermodynamic equilibrium exists between the T_1_ and T_2_ states but rather serves as a quantitative descriptor of the nonradiative process (internal conversion) between the two triplet states. The adiabatic energy gaps (Δ*E*) and relevant nonradiative rates data are listed in Tables 2 and 3.

**TABLE 2:** Calculated ISC^a^ and RISC^b^ rates for all gold complexes.

Mol	ISC^a^	Δ*E*, eV^c^	*K*_ISC_, s^−1^ d	RISC^b^	Δ*E*, eV^c^	*k*_RISC_, s^−1^ e
Au1	S_1_ → T_1_	−0.35	1.53 × 10^8^	T_1_ → S_1_	0.35	9.59 × 10^6^
	S_1_ → T_2_	−0.16	7.36 × 10^6^	T_2_ → S_1_	0.16	1.46 × 10^9^
Au2	S_1_ → T_1_	−0.36	5.13 × 10^5^	T_1_ → S_1_	0.36	6.74 × 10^5^
	S_1_ → T_2_	−0.16	5.06 × 10^7^	T_2_ → S_1_	1.97	1.27 × 10^8^
Au3	S_1_ → T_1_	−0.23	1.96 × 10^1^	T_1_ → S_1_	0.23	2.74 × 10^4^
	S_1_ → T_2_	−0.03	7.47 × 10^5^	T_2_ → S_1_	0.03	1.26 × 10^8^
Au4	S_1_ → T_1_	−0.51	3.65 × 10^4^	T_1_ → S_1_	0.51	8.55 × 10^6^
	S1 → T_2_	−0.28	1.59 × 10^4^	T_2_ → S_1_	0.28	6.07 × 10^5^
Au5	S_1_ → T_1_	−0.03	1.65 × 10^9^	T_1_ → S_1_	0.03	4.51 × 10^9^
	S_1_ → T_2_	0.37	6.48 × 10^6^	T_2_ → S_1_	0.37	1.64 × 10^6^
Au6	S_1_ → T_1_	−0.03	2.31 × 10^9^	T_1_ → S_1_	0.03	2.49 × 10^9^
	S_1_ → T_2_	0.10	2.04 × 10^7^	T_2_ → S_1_	−0.10	5.66 × 10^6^
Au7	S_1_ → T_1_	−0.03	3.49 × 10^9^	T_1_ → S_1_	0.03	4.29 × 10^9^
	S_1_ → T_2_	0.28	5.97 × 10^4^	T_2_ → S_1_	−0.28	9.05 × 10^4^

a Intersystem crossing (ISC).

b Reverse intersystem crossing (RISC).

c Singlet‐triplet energy gaps (Δ*E*).

d Internal crossing rate constant (*K*_ISC_).

e Internal crossing rate constant (*k*_RISC_).

**TABLE 3:** Theoretically predicted rates of IC/RIC^a^ processes under the framework of Marcus.

Mol	IC/RIC^a^	Δ*E*, eV^b^	*k*_IC/RIC_, s^−1^ c	*K* d
Au1	T_1_ → T_2_	−0.20	1.09 × 10^14^	4.21 × 10^4^
	T_2_ → T_1_	0.20	2.58 × 10^9^	
Au2	T_1_ → T_2_	−0.20	2.24 × 10^14^	2.00 × 10^4^
	T_2_ → T_1_	0.20	1.12 × 10^10^	
Au3	T_1_ → T_2_	−0.20	8.65 × 10^13^	1.95 × 10^3^
	T_2_ → T_1_	0.20	4.44 × 10^10^	
Au4	T_1_ → T_2_	−0.23	1.40 × 10^12^	2.85 × 10^2^
	T_2_ → T_1_	0.23	4.90 × 10^9^	
Au5	T_1_ → T_2_	−0.39	3.16 × 10^12^	1.31 × 10^2^
	T_2_ → T_1_	0.39	2.41 × 10^10^	
Au6	T_1_ → T_2_	−0.13	1.37 × 10^11^	9.19 × 10^1^
	T_2_ → T_1_	0.13	1.49 × 10^9^	
Au7	T_1_ → T_2_	−0.31	4.32 × 10^9^	9.31 × 10^1^
	T_2_ → T_1_	0.31	4.64 × 10^7^	

a Internal conversion (IC) and reverse (RIC).

b Triplet–triplet energy gaps (Δ*E*).

c Internal conversion rate constant or reverse conversion rate constant (*k*_IC/RIC_).

d Equilibrium constant *K* (*K* = *k*_IC_/*k*_RIC_).

As shown in Tables 2 and 3, for all Au1−7 complexes, the IC rate from T_1_ to T_2_ is significantly higher than the ISC rate from S_1_ to T_1_ or T_2_. Thus, the IC from T_1_ to T_2_ states occurs rapidly and preferentially over ISC processes. In addition, the equilibrium constant *K* values of Au1−3 complexes are substantially larger than those of the Au4−7 complexes. This difference can be attributed to the nature of T_1_ states: in Au1−3 complexes, the T_1_ states exhibit an LLCT character from the EAG to the EAG, which makes them more likely to undergo IC to the T_2_states with an ILCT character localized on the EAG. Therefore, the EAG with the phenylmethyl group plays an important role in the IC process from T_1_ to T_2_. Table S4 lists the phosphorescence emission rates, lifetimes, and parameters related to wavelength and oscillator strength. For the Au1−5 complexes, IC occurs from the short‐lived T_1_ state to the long‐lived T_2_ state. In contrast, for the Au6 and Au7 complexes, although the phosphorescence lifetimes of the T_2_ states are shorter than those of the T_1_ states, the IC rate constants *k*_IC_ are 1.37 × 10^11^ s^−1^ and 4.32 × 10^9^ s^−1^, respectively. Such large *k*_IC_ values enable rapid population transfer from the T_1_ state to the T_2_ state, resulting in the phosphorescence lifetimes of Au6 and Au7 complexes being shorter than those of the other Au complexes.

**FIGURE 2:**
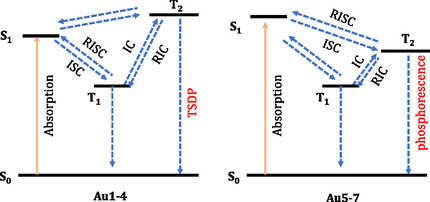
Jablonski diagrams for Au1‐Au7 complexes. ISC denotes intersystem crossing, RISC denotes reverse intersystem crossing, IC denotes internal conversion, and RIC denotes reverse internal conversion.

As illustrated by the Jablonski diagram in Figure 2, the photophysical pathway of Au1‐Au7 complexes start with light absorption, promoting excitation from ground state to singlet excited state. The excited singlet state then undergoes IC to T_1_ or T_2_state. In addition to phosphorescence emission, the T_1_ state also undergoes ultrafast IC to decay into the T_2_ state. Consequently, high‐energy phosphorescence emission occurs from the T_2_ state. The energy gaps from S_1_ to T_2_ in the Au5−7 complexes are positive, which can be attributed to the conjugation effects of their electron‐donating carbazolate groups. From this perspective, after the Au1−3 complexes are excited to the S_1_ state via light absorption, two competing nonradiative pathways toward T_1_ state and T_2_ states can occur, ultimately leading to high‐energy phosphorescence emission. This behavior makes the Au1−3 complexes promising TSDP emitters.

**FIGURE 3:**
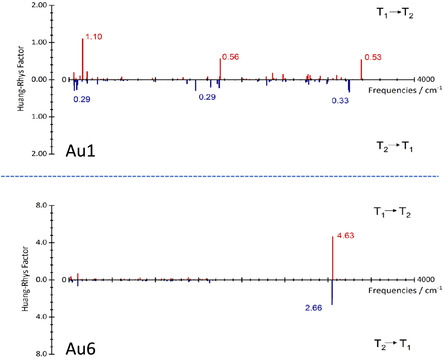
Huang–Rhys factors of Au1 and Au6 complexes.

**FIGURE 4:**
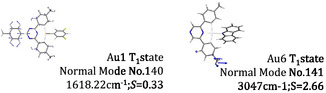
Key normal modes in nonradiative processes from T_2_ to T_1_ states of Au1 and Au6 complexes; S denotes the Huang–Rhys factor.

Taking the Au1 and Au6 complexes as representative examples, Huang–Rhys factor diagrams were further employed to evaluate the nonradiative processes from the T_1_ to the T_2_ state (see Figures 3 and S10). The Huang–Rhys factors of Au1 are distributed over several vibrational modes, indicating that multiple normal modes participate in the T_1_  →  T_2_ thermally activated nonradiative process. In contrast, Au6 exhibits a dominant Huang–Rhys factor peak at a specific high‐frequency vibrational mode, suggesting that its vibrational contribution is mainly concentrated in this mode. The broader Huang–Rhys factor distribution in Au1 may provide multiple vibrational channels for the T_1_  →  T_2_ internal conversion process. This indicates that Au1 exhibits a stronger LLCT character, which is favorable for the thermally activated delayed phosphorescence process. As illustrated in Figure 4, As illustrated in Figure 4, the dominant T1 vibrational mode of Au1 is delocalized over the entire EAG moiety. In contrast, the strongest coupled T1 vibrational mode in Au6 is primarily characterized by the torsional (rotational) motion of the methyl substituent on the EAG group. This shift results in a reduced Huang–Rhys factor, and the EAG plays a more prominent role in the photochemistry processes. This observation is consistent with our understanding that the introduction of the phenylmethyl group into the EAG significantly impacts the performance of photonic materials.

### 3.4 UV‐Vis Absorption Spectra

**FIGURE 5:**
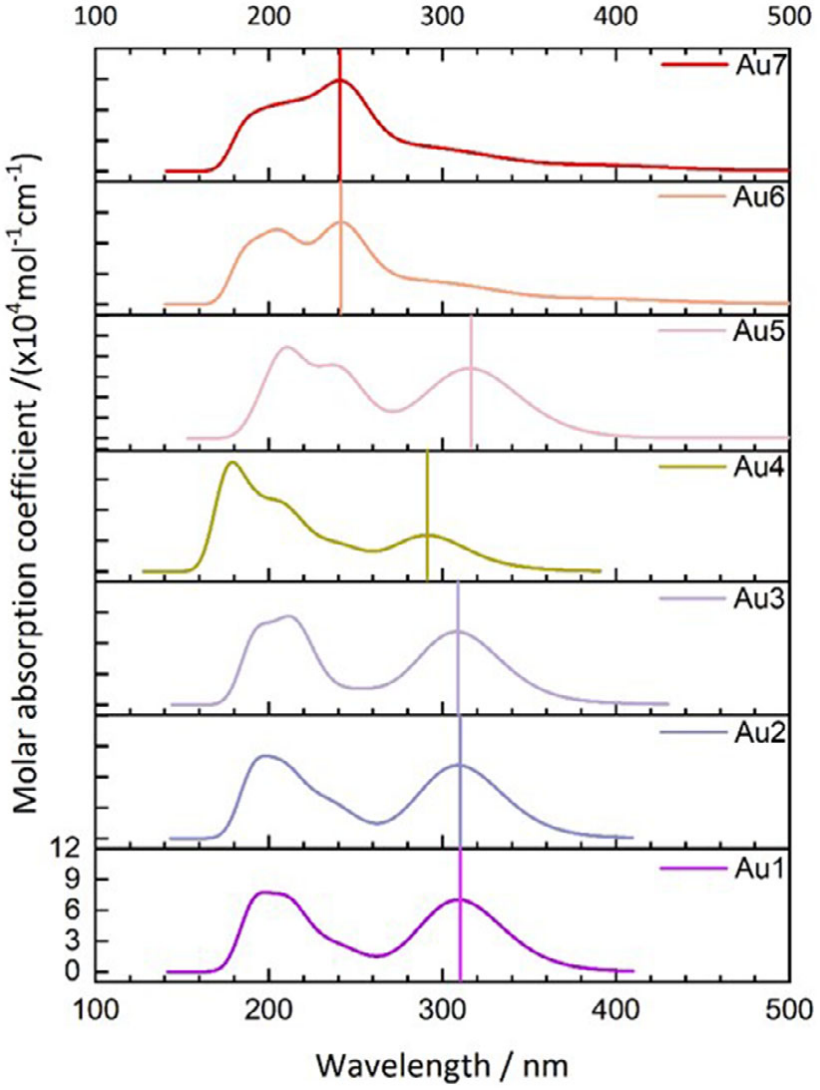
UV‐Vis spectra of Au1‐Au7 complexes calculated by TD‐DFT method combined with IEF‐PCM model in water.

The TD‐DFTsimulated of UV‐Vis absorption spectra are shown in Figure 5, showing good agreement with the experimentally observed UV‐Vis absorption spectra [7, 25]. For the Au1−3 complexes, the absorption maxima are located at approximately 310 nm. When the EAG of gold complex does not contain the phenylmethyl group, the absorption maximum of Au4 complex is blue shift to 301 nm. This indicates that the introduction of the phenylmethyl group into the EAG enhances light absorption in the visible region. Owing to the resonance effect, the absorption maxima of the Au6−7 complexes are both blue shift to around 240 nm. Notably, the Au5 complex exhibits the longest absorption maximum at 316 nm, which arises from the extension of the conjugated system due to the combined effect of the carbazolate and phenylmethyl groups.

**FIGURE 6:**
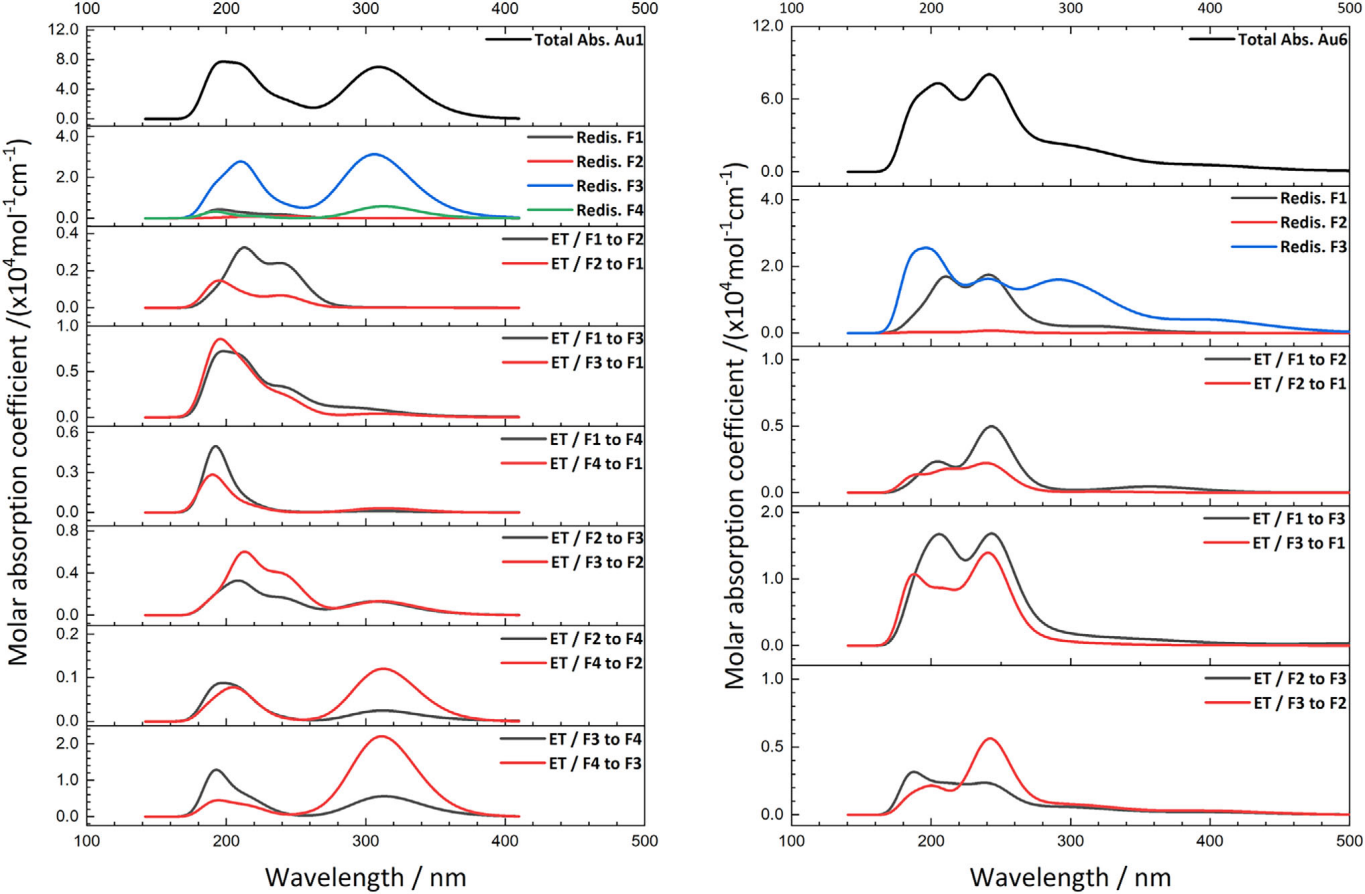
Charge‐transfer spectra (CTS) of Au1 and Au6. Redis: redistribution; ET: electron transfer; the damping constant of the Gaussian broadening function is taken to be 0.25 eV.

Based on TD‐DFT calculations, we construct the newly proposed charge transfer spectra (CTS) to analyze intrafragment charge redistribution and charge transfer. When the system can be partitioned into distinct characteristic fragments using the CTS method, that provides clear and intuitive insights into the physical origin of each absorption peak in terms of both intrafragment charge redistribution and charge transfer between different fragments. For the typical Au1 and Au6 complexes, we define the following fragments: Fragment 1 (F1) is the EDG, Fragment 2 (F2) is the gold metal (Au), Fragment 3 (F3) is the pyrazinyl electron‐accepting group (EAG), and Fragment 4 (F4) is the phenylmethyl electron‐accepting group (EAG). As shown in Figure 6, the most prominent absorption band of the Au1 complex is centered at approximately 310 nm and is mainly attributed to the LLCT from the F4  →  F3 [*π*(aryl)  →  π*(triazine)]. Therefore, the F4 fragment (phenylmethyl group) plays a key role in inducing a red shift in the absorption spectra of these gold complexes. In contrast, the most prominent absorption peak of Au6 is centered at around 243 nm, and the main contribution arises from the LLCT between F1 (EDG) and F3 (EAG) [*π*(auxiliary)  →  π*(triazine)], which is distinct from the transition features observed in the Au1 complex. As a result, different ligand groups significantly influence the excited‐state properties of these gold complexes. The molecular design for photosensitizers with enhanced visible light absorption remains an important direction for further research. The strategic optimization of EAG and EDG is crucial for developing phosphorescent materials that exhibit strong absorption across the entire visible light region.

### 3.5 Charge Decomposition Analysis

**FIGURE 7:**
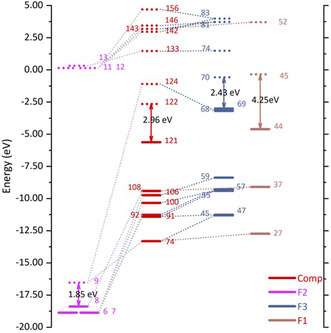
Charge decomposition analysis diagram of Au6. Virtual orbitals are marked as short dashed lines. F1 = electron‐donating group (EDG), F2 = gold metal (Au), F3 = electron‐accepting group (EAG) and Comp = Au1 complex.

In this section, a generalized charge decomposition analysis (CDA) was performed to gain a deeper understanding of the electronic structure of these gold complexes. Figure 7 presents the orbital interaction diagram for Au6 molecule as a representative example [42, 46]. By analyzing the role of fragment orbitals (FOs), it is possible to clarify how these FOs participate in the chemical interactions within the molecular orbitals of the complexes [47, 48]. The bonding orbitals for coordination structure between the metal center and organic ligands in the Au6 complex have been thoroughly discussed in the following analysis. The complete CDA results, including all contributions of FOs to molecular orbitals, are provided in Supporting Information.

Figure 7 presents the orbital interaction diagram of the Au6 molecule. According to the effective atomic number (EAN) principle and the complete CDA results, the Au(III) complex achieves a stable structure with six bonding orbitals that collectively contain 12 electrons. Molecular orbital 74 (MO74) is formed by the bonding of Au^3+^ with the lone pair electrons of nitrogen atoms from F1. MO91, MO92, MO100, and 106 are attributed to coordination bonds contributed by the nitrogen atoms of the F3 (EAG). All 5d orbitals of the Au atom are involved in the formation of coordination bonds, contributing to the stability of the complex. Table S2 and Figures S4–S8 show that the molecular orbital (MO) 121 (HOMO) of Au6 is primarily composed of FO44 (F1 HOMO‐89.70%), and the orbital (MO) 122 (LUMO) is composed of FO71 (F3 HOMO‐88.88%). Additionally, the HOMO → LUMO excitation with a contribution of 0.989 represents the first excited state S_1_, exhibiting ligand‐to‐ligand charge transfer (F1–F3) characteristics. This observation is consistent with the CTS spectrum analysis.

## 4 Conclusion

This study presents a theoretical investigation of seven gold(III) complexes, including experimentally reported species (Au1 ∼ Au4 and Au6 ∼ 7) and the newly theoretically designed Au5 complex. Au5 was designed by introducing a carbazolate group as the EDG and a phenylmethyl group as the EAG. The semiclassical Marcus theory is employed to calculate the ISC rates between the singlet and triplet states, as well as the IC rates, enabling a microscopic elucidation of the photochemical pathways. Upon photoexcitation, the system undergoes ISC to T_1_ or T_2_ states. In the T_1_ states, the phosphorescent emission and IC to T_2_ state can occur simultaneously, and the complexes ultimately emit high‐energy phosphorescence from T_2_ state. Analysis of electron–hole orbitals reveals that the LLCT transition character of the T_1_ is highly sensitive to the environment, facilitating internal conversion to the T_2_ state, which exhibits ILCT transition character. The Au1 ∼ 3 complexes exhibit higher IC rate from T_1_ to T_2_ than the Au4 ∼ 7 complexes. Huang–Rhys factor analysis indicates that the dominant vibrational normal mode of the T_1_ state associated with the strongest vibronic coupling mainly corresponds to the vibrations of the EAG (phenylmethyl group), which explains why the nonradiative T_1_ to T_2_ process is more favorable in Au1‐Au3 than in the other Au complexes. Therefore, the gold (III) complexes containing EAG (phenylmethyl triazine) are promising candidates for efficient thermally activated delayed phosphorescence (TADP) emitters. Additionally, the calculated UV‐visible absorption and CTS spectra reveal that the designed Au5 complex, featuring a carbazolate EDG and a phenylmethyl EAG, exhibits significantly enhanced visible light absorption. This improvement enables a broader emission spectrum spanning from saturated blue to saturated red, making Au5 a highly promising phosphorescent material. Overall, this work provides valuable insights and guidance for the future molecular design of related luminescent materials.

## Conflicts of Interest

The authors declare no conflicts of interest.

## Supporting information

The authors have cited additional references within the Supporting Information [36, 42, 47].

## Data Availability

The data that support the findings of this study are available from the corresponding author upon reasonable request.
