# Supplementary material for: Computational Study of the Photophysical Properties and Electronic Structure of Gold (III) Complexes with Different Groups
Source: ChemistryOpen. 2026 Jul 13;15(7):e70248. doi: 10.1002/open.70248 (PMC13359157; doi:10.1002/open.70248)
Supplement: Supplementary file 1 — The authors have cited additional references within the Supporting Information [36, 42, 47]. [file OPEN-15-e70248-s001.pdf]

Computational Study of the Photophysical Properties and Electronic Structure of Gold (III) Complexes with Different Groups

Caijie Bu,[a,b] Tao Yuan,[b] Han Xiao[b], Jing Wei*[b,c] and Minyi Zhang*[b,c]

[a] Caijie Bu
College of Chemistry and Materials Science, Fujian Normal University
Qishan Campus, No.18 Middle Wulongjiang Avenue, Shangjie, Minhou, Fuzhou, P. R. China
Postcode: 350117

[b] Caijie Bu, Tao Yuan, Jing Wei, Minyi Zhang
State Key Laboratory of Structural Chemistry, Fujian Institute of Research on the Structure of Matter, Chinese Academy of Sciences
No.8, Gaoxindadao Road, Shangjie, Minhou, Fuzhou, P. R. China Postcode: 350108
E-mail: myzhang@fjirsm.ac.cn, weijing@fjirsm.ac.cn

[c] Jing Wei, Minyi Zhang
Fujian College, University of Chinese Academy of Sciences
No.8, Gaoxindadao Road, Shangjie, Minhou, Fuzhou, P. R. China Postcode: 350108
E-mail: myzhang@fjirsm.ac.cn, weijing@fjirsm.ac.cn

1.Supplemental Figures

1.1 Comparison of Structures with RMSD Value

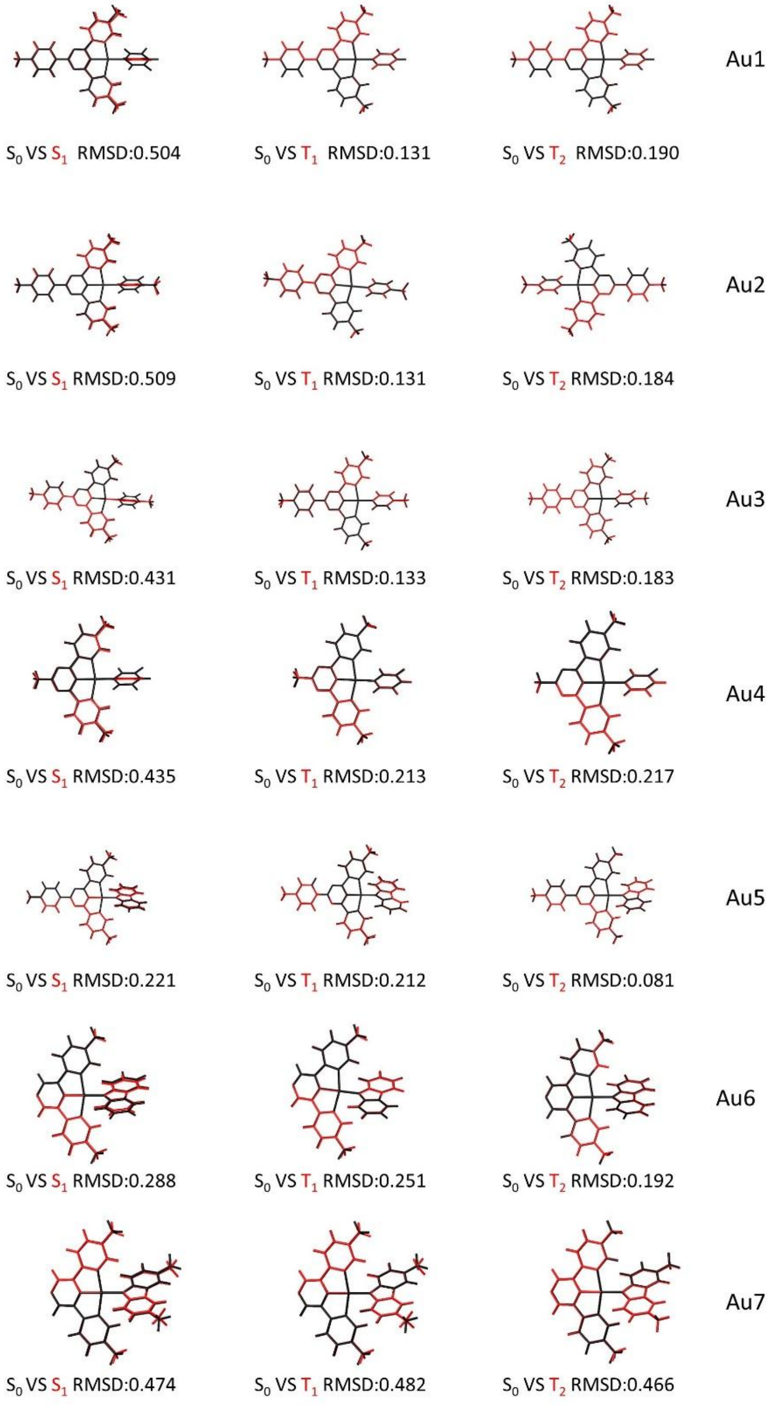

Figure S1. Comparison of Au1-Au7 structures of ground state (black) and excited states (red) with RMSD of total structure.

1.2 The Real-space Distribution (S1, T1 and T2 states) of Holes and Electrons

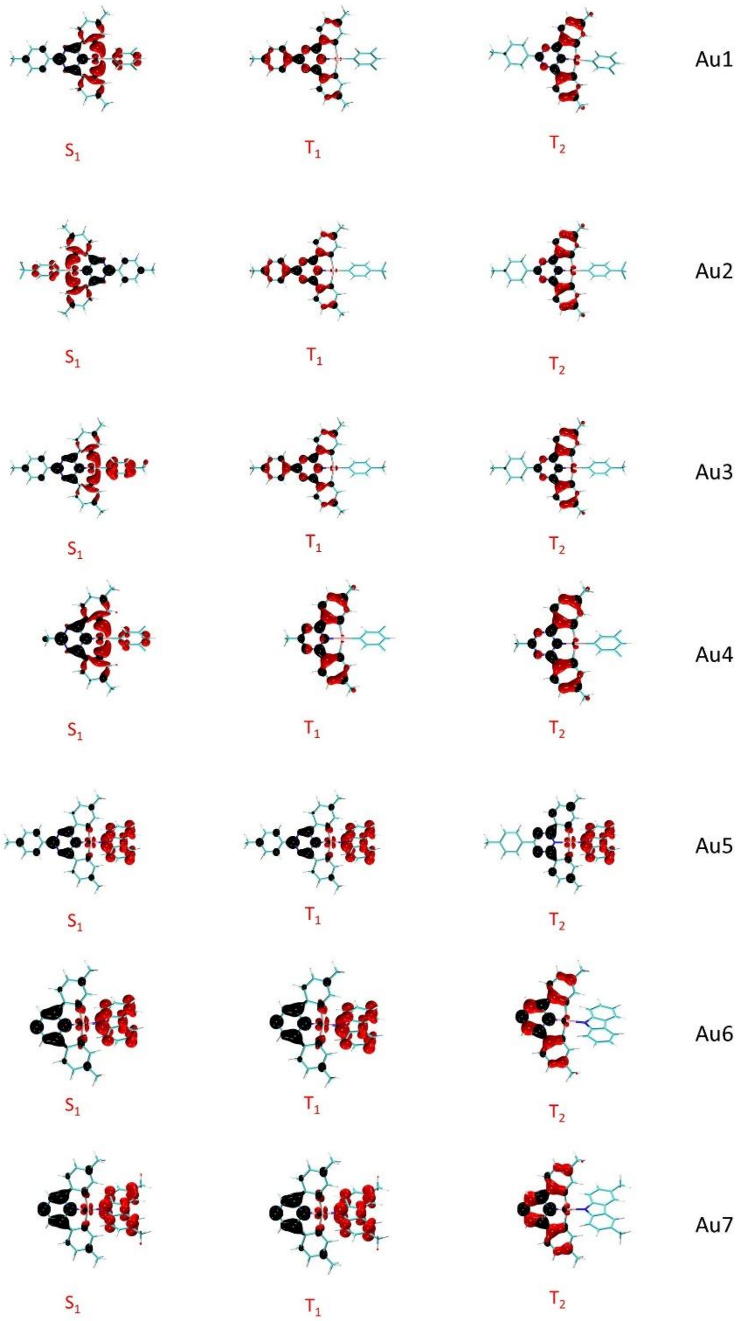

Figure S2. The ground state (S0) structure-based hole-electrons analysis related to excitation features of the S_1, T_1 and T_2 states in this work.

1.3 Old Electron Density (OED) Analysis of Au Complexes.

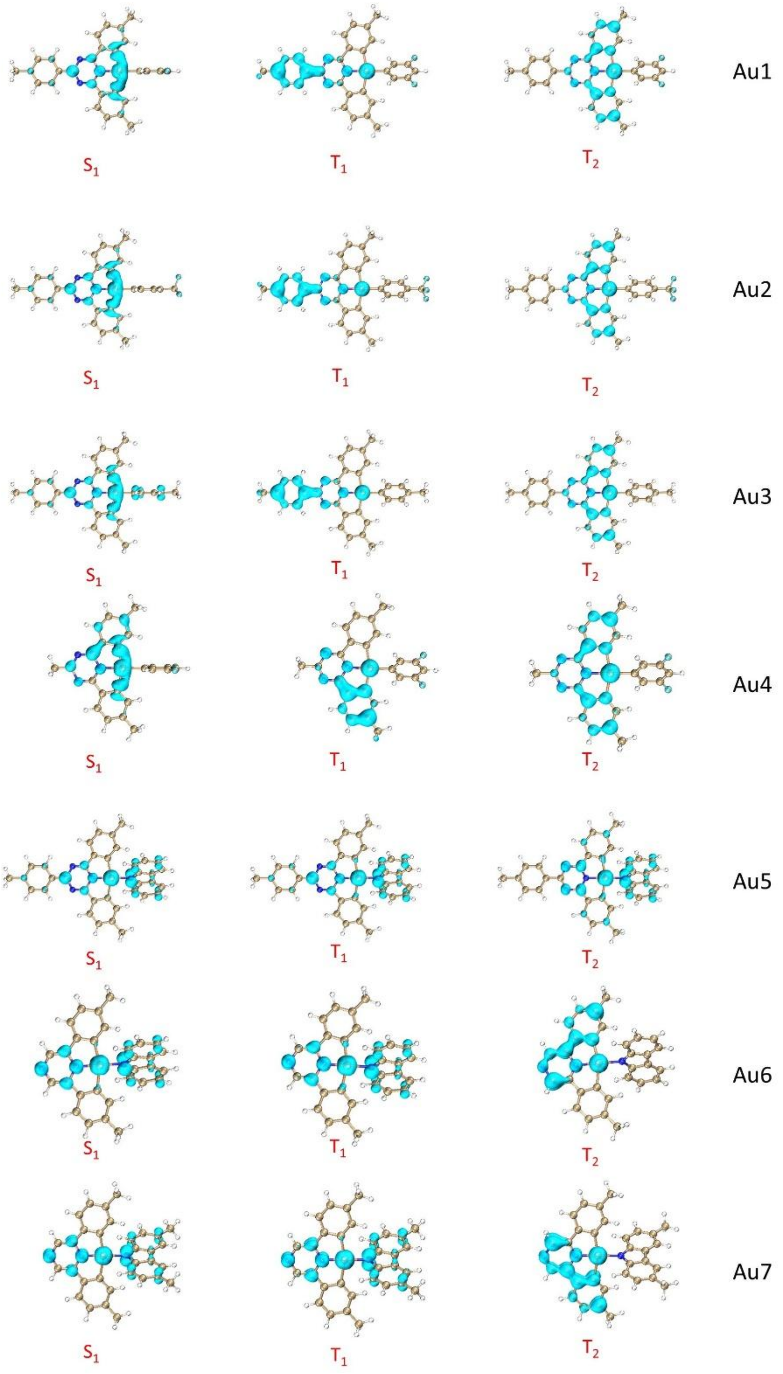

Figure S3. The old electron density analys related to excitation states of the S_1, T_1 and T_2..

1.4 Isosurfaces of Molecular Orbitals

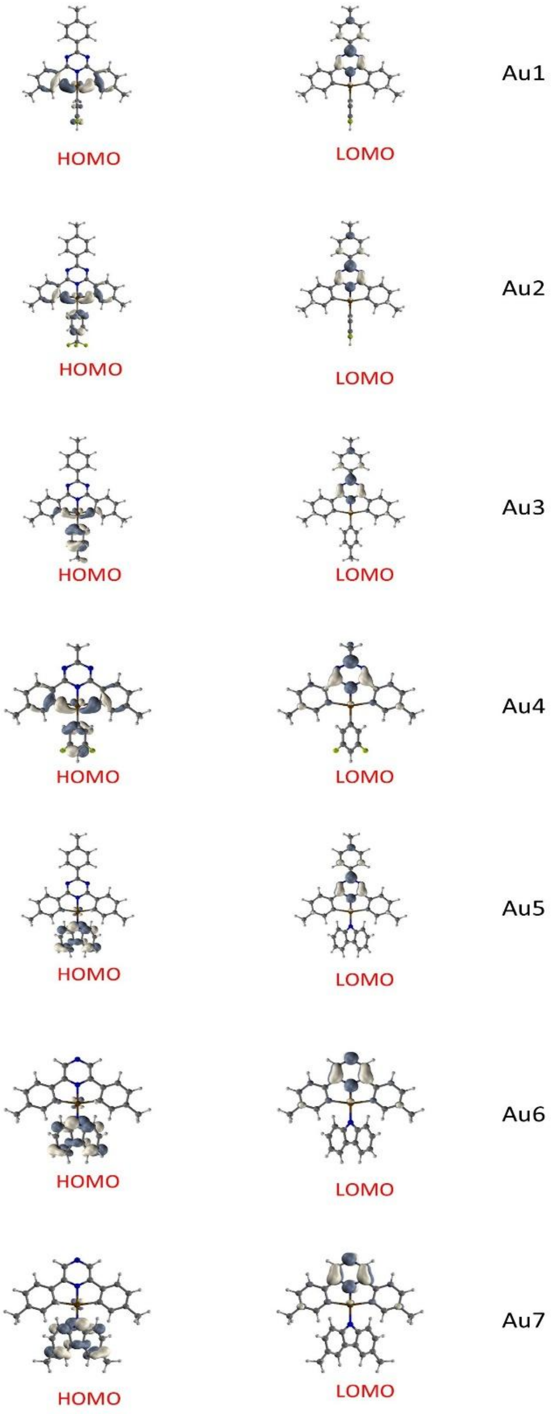

Figure S4. Molecular orbitals of Au1~Au7: HOMO and LUMO.

1.5 Isosurface of Fragment or Complex Orbitals

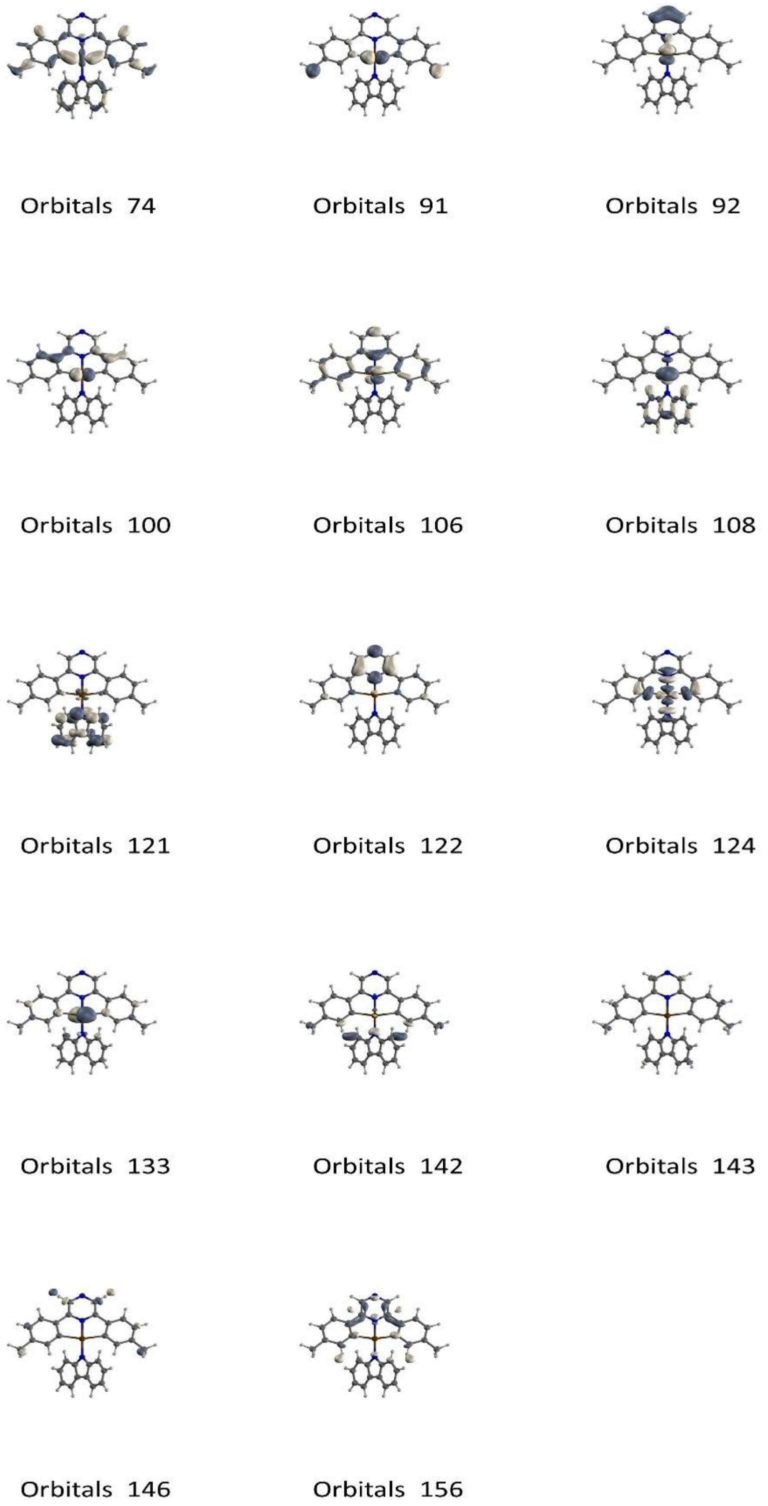

Figure S5. Complexes orbitals of Au6.

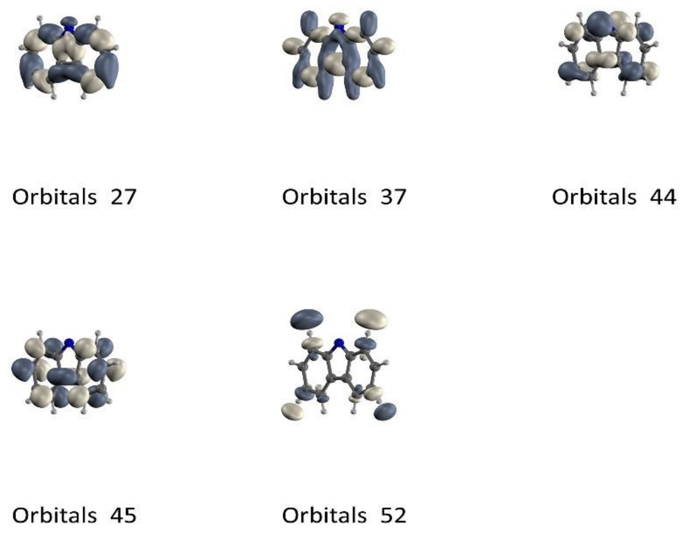

Figure S6. Fragment orbitals of F1 in Au6**.**

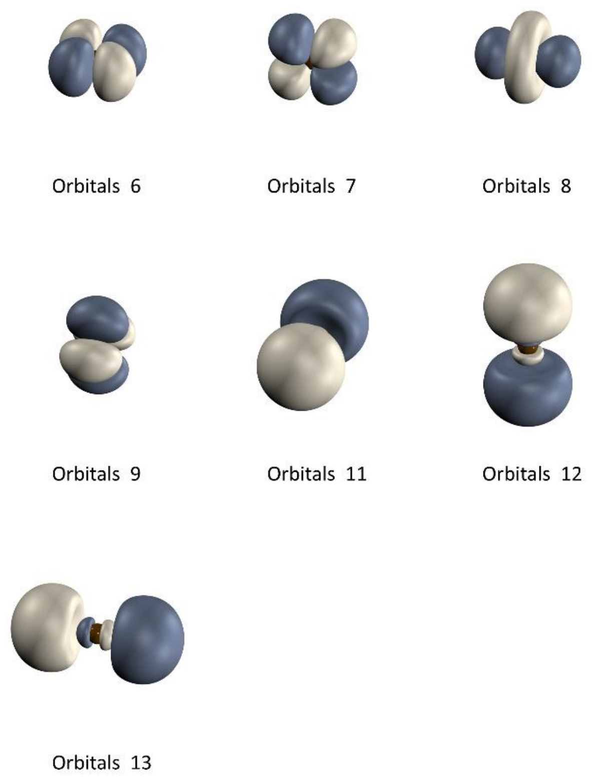

Figure S7. Fragment orbitals of F2 in Au6.

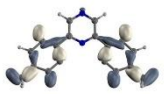

Orbitals 45

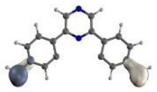

Orbitals 47

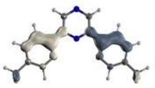

Orbitals 55

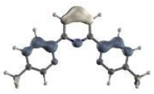

Orbitals 57

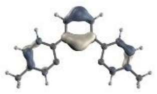

Orbitals 59

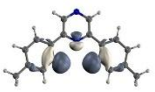

Orbitals 68

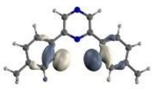

Orbitals 69

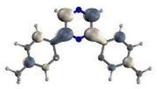

Orbitals 70

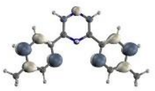

Orbitals 74

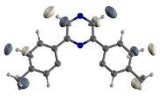

Orbitals 81

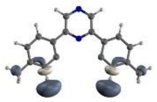

Orbitals 83

Figure S8. Fragment orbitals of F3 in Au6.

1.6 Density of States of Au6

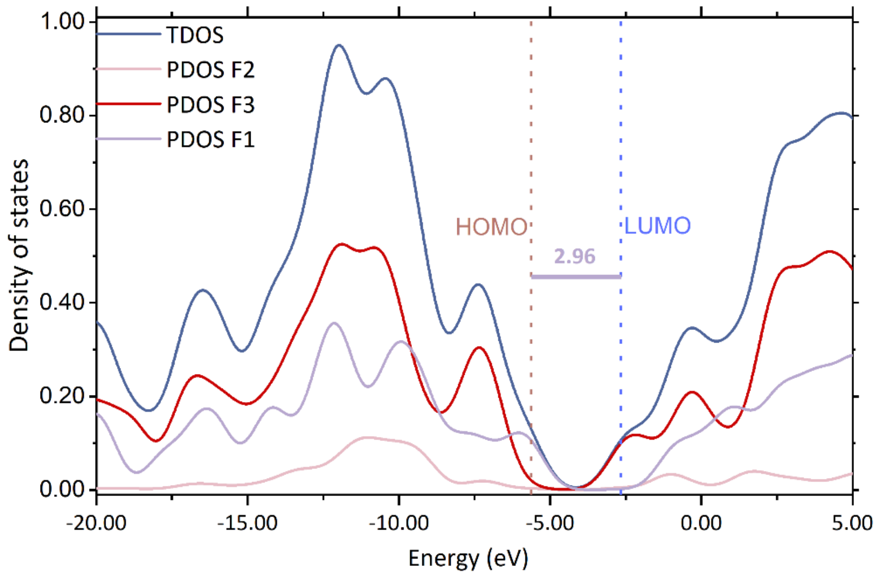

Figure S9. Total Density of States (TDOS) and Partial Density of States (PDOS) of Au6. F1 stands for electron-donating group, F2 stands for Au^{3+} and F3 stands for electron-accepting group.

1.7 Most Strongly Coupled Normal Modes of Vibration

Au1

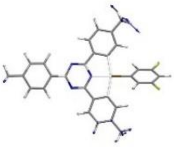

Au1 T_1state
Normal Mode No.4
30.52cm^{-1};S=0.29

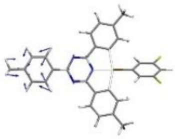

Au1 T_1state
Normal Mode No.61
733.63cm^{-1};S=0.29

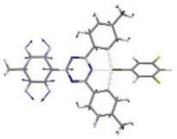

Au1 T_1state
Normal Mode No.140
1618.22cm^{-1};S=0.33

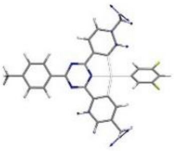

Au1 T_2state
Normal Mode No.11
78.96cm^{-1};S=1.10

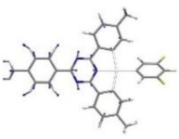

Au1 T_2state
Normal Mode No.74
875.37cm^{-1};S=0.56

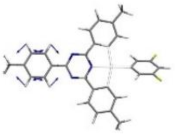

Au1 T_2state
Normal Mode No.145
30.52cm^{-1};S=0.53

Au6

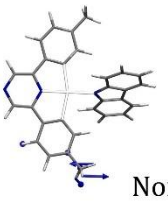

Au6 T_1state
Normal Mode No.141
3047cm^{-1};S=2.66

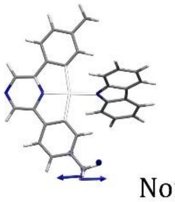

Au6 T_2state
Normal Mode No.142
3053cm^{-1};S=4.63

Figure S10. Most Strongly Coupled Normal Modes of Vibration of Au1 and Au6.

2.Calculation Result

2.1 Key Geometrical Parameters

Table S1. Calculated dihedral angles of Au1~7compleses in their S0, S1, T1 and T2 optimized structure. (in degree).

complexes \ states		S0	S1	T1	T2
Au1	D1	56.83	98.63	57.11	57.27
	D2	0.06	1.82	0.02	0.03
Au2	D1	56.65	98.27	56.90	57.02
	D2	0.12	2.05	2.33	0.13
Au3	D1	54.75	90.52	54.96	55.54
	D2	0.12	0.31	0.06	0.12
Au4	D1	56.80	82.68	56.56	56.97
	D2	——	——	——	——
Au5	D1	61.22	66.45	62.71	62.68
	D2	0.01	0.14	0.45	0.06
Au6	D1	62.56	69.52	64.41	63.69
	D2	——	——	——	——
Au7	D1	62.69	68.15	64.80	63.72
	D2	——	——	——	——

2.2. Electron-hole analysis of Au 1~7

Table S2. The electron-hole decomposition of fragments F1-F3 in complexes Au1~7 complexes (F1 = electron-donating group, F2 = Au metal, F3 = tridentate pyrazine group, F4 = benzyl group).

complexes \ states		Fragment1%		Fragment2%		Fragment3%		Fragment4%	
		Hole	Electron	Hole	Electron	Hole	Electron	Hole	Electron
Au1	S1	17.31	0.95	8.77	5.75	73.79	78.37	0.13	14.93
	T1	0.44	0.22	1.15	1.49	70.90	77.97	27.51	20.30
	T2	0.15	0.50	1.93	2.79	97.92	92.56	0.00	4.46
Au2	S1	15.86	0.97	8.92	5.77	75.09	78.31	0.13	14.95
	T1	0.17	0.09	1.17	1.39	70.98	78.05	27.68	20.47
	T2	-0.05	0.47	1.95	2.77	98.16	92.35	-0.06	4.41
Au3	S1	39.25	0.99	8.74	5.79	51.86	77.63	0.14	15.59
	T1	0.52	0.23	1.70	1.26	71.28	78.19	26.60	20.32
	T2	-0.09	0.44	1.90	2.61	98.16	92.55	0.03	4.39
Au4	S1	16.29	1.32	8.74	7.41	74.97	91.27	——	——
	T1	0.03	0.40	1.02	1.33	98.94	98.61	——	——
	T2	-0.20	0.56	1.76	2.62	98.44	96.81	——	——
Au5	S1	88.81	0.37	3.23	5.82	7.84	56.58	0.07	16.70
	T1	87.02	0.33	3.55	6.03	9.10	57.16	0.24	16.24
	T2	85.68	0.23	3.46	0.21	10.67	98.31	0.12	0.76
Au6	S1	87.68	0.49	3.30	7.03	9.02	92.48	——	——
	T1	85.27	7.20	3.61	7.20	11.11	92.31	——	——
	T2	-0.03	0.07	1.57	4.50	95.43	96.93	——	——
Au7	S1	89.99	0.50	2.93	7.00	7.08	92.50	——	——
	T1	88.42	0.49	3.17	7.15	8.41	92.36	——	——
	T2	-0.05	0.29	1.58	4.32	98.47	95.39	——	——

2.3. Contributions of the main transition orbitals.

Table S3. The contributions of the main transition orbitals for each excited state (S_1, T_1, T_2) in Au1~7 complexes.

complexes \ states		MO	MO contribution(%)[a]
Au1	S1	H→L	97.9
	T1	H-2→L	54.8
	T2	H-1→L	54.5
Au2	S1	H→L	98.1
	T1	H-2→L	58.3
	T2	H-1→L	52.9
Au3	S1	H→L	95.6
	T1	H-2→L	77.6
	T2	H-1→L	53.5
Au4	S1	H→L	98.0
	T1	H→L	83.2
	T2	H-1→L	52.4
Au5	S1	H→L	98.8
	T1	H→L	97.8
	T2	H→L+1	98.1
Au6	S1	H→L	98.9
	T1	H→L	98.0
	T2	H-2→L	76.4
Au7	S1	H→L	99.2
	T1	H→L	98.6
	T2	H-2→L	75.2

[a] MO contribution is calculated by the Multiwfn program.

2.4 Calculated Phosphorescence Lifetime

Table S4. Calculated phosphorescence emission lifetime (τ)

Mol	Kp	f/a.u.	lambda/nm	KF	τ(s)[a]
Au1	T1→S0	1.24×10^{-5}	183.35	2.64×10^{4}	4.06×10^{-4}
	T2→S0	4.91×10^{-7}	1211.88	2.23×10^{1}	4.48×10^{-2}
Au2	T1→S0	1.84×10^{-7}	117.39	8.91×10^{2}	1.12×10^{-3}
	T2→S0	8.35×10^{-7}	1215.11	3.77×10^{1}	2.65×10^{-2}
Au3	T1→S0	5.70×10^{-7}	145.51	1.79×10^{3}	5.57×10^{-4}
	T2→S0	1.20×10^{-6}	1412.86	4.01×10^{1}	2.49×10^{-2}
Au4	T1→S0	1.08×10^{-7}	4606.54	3.38×10^{1}	2.96×10^{0}
	T2→S0	4.54×10^{-7}	1577.42	1.22×10^{1}	8.22×10^{-2}
Au5	T1→S0	6.60×10^{-6}	1468.74	2.04×10^{2}	4.90×10^{-3}
	T2→S0	4.98×10^{-7}	1483.22	151×10^{1}	6.62×10^{-2}
Au6	T1→S0	1.49×10^{-9}	2678.80	1.38×10^{-2}	7.23×10^{1}
	T2→S0	2.12×10^{-6}	1311.03	8.24×10^{1}	1.21×10^{-2}
Au7	T1→S0	1.63×10^{-9}	2596.38	1.61×10^{-2}	6.20×10^{1}
	T2→S0	1.36×10^{-6}	1232.03	5.99×10^{1}	1.67×10^{-2}

[a] Phosphorescence emission lifetime is calculated by the Dalton program.[18a]

2.5 Geometric Structures in Cartesian

Structure of Au1, S0 state

C	1.56220157	-1.16840687	0.04260256
C	3.50630517	0.00006826	0.00259875
C	1.56220112	1.16845547	-0.04001758
N	2.88673022	1.19449728	-0.03954246
N	2.88673489	-1.19439488	0.04374948
C	0.66669151	-2.32643033	0.08647908
C	1.15108259	-3.63816053	0.12737193
C	-0.73559546	-2.05880357	0.08175227
C	0.25656492	-4.70112668	0.16764012
H	2.23012822	-3.81195539	0.12366240
C	-1.59921699	-3.14829619	0.12527968
C	-1.12828152	-4.47356751	0.17053407
H	0.63388361	-5.72671455	0.19554754
H	-2.68025956	-2.97727829	0.11995061
C	-2.09299436	-5.61882014	0.24965936
H	-2.37109023	-5.81395249	1.29924707
H	-3.02271020	-5.40011728	-0.29536605
H	-1.65840134	-6.54546627	-0.15136416
C	0.66670220	2.32644860	-0.08488632
C	1.15109770	3.63818517	-0.12536030
C	-0.73558551	2.05878446	-0.08146847
C	0.25658796	4.70113121	-0.16650250
H	2.23013495	3.81200774	-0.12063992
C	-1.59919066	3.14825020	-0.12582040
C	-1.12824379	4.47353713	-0.17066696
H	0.63390703	5.72672725	-0.19408608
H	-2.68023415	2.97720792	-0.12148284
C	-2.09294900	5.61873499	-0.25067256
H	-1.65835890	6.54569842	0.14960969
H	-2.37111735	5.81300849	-1.30039903
H	-3.02264565	5.40044743	0.29456080
Au	-1.11581289	-0.00001370	-0.00001402
C	-3.12927929	-0.00002461	-0.00089007
C	-3.84020637	-0.70096130	-0.98598942
C	-3.84098795	0.70090042	0.98365627
C	-5.23033213	-0.68082123	-0.96913047
H	-3.33575306	-1.26771456	-1.77079389
C	-5.23109825	0.68072951	0.96572388
H	-3.33715079	1.26765966	1.76885199
C	-5.95950146	-0.00005535	-0.00198533
H	-7.04970669	-0.00006992	-0.00240555

F	-5.89671686	-1.34547993	-1.91692484
F	-5.89823115	1.34537012	1.91300416
C	4.97758930	0.00011061	0.00322794
C	5.69213864	-1.20737475	0.04658210
C	5.69205429	1.20779000	-0.03563606
C	7.08089354	-1.20237154	0.04948216
H	5.14028839	-2.14792932	0.08087248
C	7.08080776	1.20309706	-0.03238048
H	5.14014441	2.14846451	-0.06527418
C	7.80263484	0.00035986	0.00740927
H	7.62193718	-2.15168567	0.08744263
H	7.62179520	2.15281859	-0.05898243
C	9.30093555	-0.00063374	-0.02276586
H	9.71510367	-0.87652079	0.49664463
H	9.66300082	-0.03730346	-1.06417209
H	9.71510880	0.90956253	0.43385484
N	0.90761927	0.00001249	0.00095801

Structure of Au2, S0 state

C	-2.03608251	1.16754178	0.01187862
C	-3.97608032	-0.00809797	-0.00501593
C	-2.02790900	-1.17024275	-0.01257830
N	-3.35248014	-1.20116251	-0.01544584
N	-3.36082467	1.18924038	0.00865335
C	-1.14411207	2.32940564	0.02918565
C	-1.63333686	3.63999815	0.03678943
C	0.25930895	2.06692854	0.03418573
C	-0.74302673	4.70710150	0.05313138
H	-2.71300202	3.80963761	0.02633591
C	1.11848669	3.16080036	0.05304658
C	0.64266034	4.48499421	0.06485547
H	-1.12430846	5.73160934	0.05519486
H	2.20030515	2.99450841	0.05483230
C	1.60261886	5.63572359	0.11860220
H	1.86969952	5.86361884	1.16444729
H	2.53830135	5.40446966	-0.41075229
H	1.16850276	6.54822767	-0.31419580
C	-1.12774431	-2.32584517	-0.02558383
C	-1.60760449	-3.63982409	-0.03462729
C	0.27386666	-2.05346834	-0.02465363
C	-0.70960977	-4.70062786	-0.04641474
H	-2.68607671	-3.81715879	-0.02883189
C	1.14084954	-3.14112512	-0.03898633
C	0.67444277	-4.46871740	-0.05205225

H	-1.08358922	-5.72781667	-0.04960308
H	2.22145412	-2.96705304	-0.03602538
C	1.64324157	-5.61225426	-0.10040984
H	1.20869664	-6.53178270	0.31668376
H	1.92985278	-5.82809125	-1.14362337
H	2.56826453	-5.37921907	0.44671547
Au	0.64797231	0.00801696	0.00525717
C	2.66049286	0.01446001	0.00868324
C	3.38031320	0.69354408	-0.98693916
C	3.38159031	-0.65701169	1.00837568
C	4.77383442	0.69463754	-0.99119189
H	2.85025904	1.23685201	-1.77316384
C	4.77518291	-0.64724116	1.01812759
H	2.85265745	-1.19768335	1.79718910
C	5.47395195	0.02544717	0.01428187
C	-5.44748774	-0.01307048	-0.00844168
C	-6.16605515	1.19273420	-0.00026164
C	-6.15795900	-1.22362480	-0.02398226
C	-7.55481830	1.18327986	-0.00625120
H	-5.61732849	2.13570954	0.00778247
C	-7.54671294	-1.22340168	-0.02985066
H	-5.60290534	-2.16284876	-0.03442042
C	-8.27256511	-0.02246084	-0.01843290
H	-8.09901408	2.13154075	-0.00372314
H	-8.08450018	-2.17518751	-0.04591275
C	-9.77093816	-0.02795864	0.00849360
H	-10.13530159	-0.04560365	1.04959144
H	-10.18715868	0.87164015	-0.46684835
H	-10.18093646	-0.91528665	-0.49459911
H	5.32054337	-1.16406199	1.81103653
H	5.31803978	1.22772141	-1.77413419
C	6.97218392	-0.01379681	-0.01428040
F	7.43172347	-1.05386942	-0.73022504
F	7.49711792	-0.13323871	1.21137585
F	7.49443616	1.08854344	-0.56637315
N	-1.37778965	0.00092711	0.00102259

Structure of Au3, S0 state

C	1.46030362	1.16821837	0.01524163
C	3.40388664	-0.00123111	0.00133045
C	1.45898367	-1.16852198	-0.01187570
N	2.78415771	-1.19612236	-0.01262808
N	2.78551517	1.19435219	0.01491315
C	0.56442694	2.32753919	0.03130925

C	1.05125049	3.63904216	0.04402471
C	-0.83896021	2.06149946	0.03128075
C	0.15921413	4.70478682	0.06134918
H	2.13067264	3.81061325	0.03721939
C	-1.69916940	3.15477962	0.05188091
C	-1.22596115	4.47980927	0.06916685
H	0.53868085	5.72998275	0.06767812
H	-2.78051704	2.98549189	0.05058376
C	-2.18917568	5.62793775	0.12406314
H	-1.74713000	6.55020367	-0.27887009
H	-3.11123580	5.40609450	-0.43277333
H	-2.48308997	5.83270621	1.16747683
C	0.56183205	-2.32682795	-0.02810789
C	1.04739289	-3.63897390	-0.04864494
C	-0.84099507	-2.05935325	-0.02877543
C	0.15442787	-4.70346568	-0.06953450
H	2.12669394	-3.81144894	-0.05087727
C	-1.70257499	-3.15186920	-0.05340518
C	-1.23088662	-4.47714080	-0.06951890
H	0.53280993	-5.72892741	-0.08892634
H	-2.78364399	-2.98104014	-0.06075986
C	-2.19325234	-5.62724691	-0.05842091
H	-1.76902174	-6.51699860	-0.54541115
H	-2.44119066	-5.90958555	0.97888030
H	-3.13762266	-5.36783382	-0.55817934
Au	-1.22435424	0.00130245	0.00005356
C	-3.23928802	0.00189546	-0.00455631
C	-3.96699668	-0.70127594	0.96656044
C	-3.96199746	0.70932608	-0.97632924
C	-5.36302016	-0.68989381	0.96751087
H	-3.44239555	-1.26760272	1.74121109
C	-5.35804430	0.70363178	-0.98038312
H	-3.43348943	1.28033694	-1.74484581
C	-6.08668502	0.00601772	-0.00889082
C	4.87580773	-0.00205569	0.00102663
C	5.59110416	1.20553816	0.01696442
C	5.58973063	-1.21048005	-0.01128587
C	6.97995187	1.19994213	0.01917692
H	5.03968455	2.14687683	0.03059327
C	6.97860258	-1.20650872	-0.00902282
H	5.03725763	-2.15125870	-0.01959151
C	7.70115178	-0.00368483	0.00350972
H	7.52144145	2.14963940	0.03563853
H	7.51901845	-2.15693617	-0.01482651

C	9.19952430	-0.00407872	-0.02745782
H	9.56133191	0.01065067	-1.06948975
H	9.61438045	0.88222736	0.47344494
H	9.61347185	-0.90467423	0.44803215
H	-5.89301220	1.26199680	-1.75512349
H	-5.90194735	-1.23867513	1.74633818
C	-7.58864224	-0.01852662	-0.03031040
N	0.80591601	0.00022378	0.00268555
H	-8.00148323	0.90135103	-0.46993403
H	-7.96113818	-0.86294633	-0.63471417
H	-8.00531532	-0.13528517	0.98094408

Structure of Au4, S0 state

C	2.61203992	1.16784929	0.04665560
C	2.61208700	-1.16830602	-0.03883832
N	3.94036459	-1.19465151	-0.03557000
N	3.94038518	1.19394839	0.05056767
C	1.71722891	2.32532672	0.09189001
C	2.20326753	3.63631932	0.13552061
C	0.31457450	2.05866058	0.08552025
C	1.30969488	4.70008034	0.17661277
H	3.28271521	3.80748202	0.13331468
C	-0.54779340	3.14880977	0.12998270
C	-0.07527786	4.47368226	0.17770451
H	1.68786425	5.72527731	0.20666204
H	-1.62903905	2.97911513	0.12359971
C	-1.03961037	5.61916542	0.25711567
H	-1.95985553	5.40971788	-0.30741087
H	-1.33646109	5.79835663	1.30437796
H	-0.59716129	6.55107560	-0.12234102
C	1.71719782	-2.32570695	-0.08517930
C	2.20315237	-3.63679747	-0.12622631
C	0.31454804	-2.05884603	-0.08272492
C	1.30953699	-4.70047858	-0.16905938
H	3.28256266	-3.80812279	-0.12066799
C	-0.54783324	-3.14890263	-0.12875141
C	-0.07537584	-4.47388765	-0.17424110
H	1.68764279	-5.72575156	-0.19720391
H	-1.62907200	-2.97907452	-0.12526616
C	-1.03978108	-5.61919045	-0.25537946
H	-0.59733419	-6.55180221	0.12231626
H	-1.95982683	-5.41062129	0.30982680
H	-1.33710542	-5.79641820	-1.30283626
Au	-0.06545308	-0.00005512	0.00073114

C	-2.07860646	0.00013738	-0.00155411
C	-2.79066016	-0.70261341	0.98132985
C	-2.78849456	0.70303465	-0.98586811
C	-4.18076886	-0.68219743	0.96252300
H	-2.28729635	-1.27082909	1.76575321
C	-4.17865344	0.68293460	-0.96988736
H	-2.28342275	1.27116177	-1.76926026
C	-4.90842598	0.00045498	-0.00442719
H	-5.99862983	0.00058307	-0.00553418
F	-4.84854986	-1.34845896	1.90811649
F	-4.84433888	1.34936246	-1.91684024
C	6.04221069	0.00052357	-0.02195848
H	6.44025890	0.89112761	0.47934763
H	6.37316050	0.03020565	-1.07374149
H	6.44000485	-0.91767544	0.42723094
C	4.55283857	-0.00032234	0.00782827
N	1.95999472	-0.00014893	0.00229666

Structure of Au5, S0 state

Au	-0.70171327	0.00019099	0.00044928
C	-0.35148496	-2.04305023	-0.26509435
C	1.04575452	-2.31612324	-0.29480848
C	1.52185190	-3.62004114	-0.46627718
C	0.61531649	-4.66383040	-0.61261229
C	-0.76798716	-4.42513673	-0.59596103
C	-1.22998984	-3.10690895	-0.42144672
H	2.59904057	-3.80357975	-0.48185864
H	0.98233471	-5.68517293	-0.74217849
H	-2.30847855	-2.92351601	-0.39948912
C	-1.74736586	-5.54540532	-0.77779275
H	-1.27511106	-6.52814106	-0.64224532
H	-2.58643305	-5.45715237	-0.07135676
H	-2.17914023	-5.51763277	-1.79214225
C	1.93222845	-1.16593778	-0.14381363
C	1.93254317	1.16526862	0.14764014
C	1.04635760	2.31582488	0.29752601
C	1.52275953	3.61957522	0.46939137
C	-0.35096090	2.04329096	0.26630467
C	0.61645645	4.66373396	0.61457491
H	2.59999926	3.80270480	0.48617845
C	-1.22923269	3.10749371	0.42155780
C	-0.76691512	4.42557333	0.59639864
H	0.98372134	5.68495057	0.74443213
H	-2.30776594	2.92450027	0.39847057

C	-1.74610246	5.54622808	0.77687656
H	-2.18018284	5.51794319	1.79022229
H	-1.27305538	6.52881465	0.64304582
H	-2.58363564	5.45889400	0.06849908
C	-3.51411111	0.65710415	-0.89941846
C	-3.51521841	-0.65623505	0.89706066
C	-3.15643118	1.45105639	-1.99960085
C	-4.88495002	0.42716437	-0.58310650
C	-3.15890663	-1.45013274	1.99772596
C	-4.88566582	-0.42664332	0.57879947
C	-4.17309663	2.01492542	-2.76180635
H	-2.10533110	1.62406510	-2.24248135
C	-5.89014511	1.00460905	-1.36990118
C	-4.17651432	-2.01429069	2.75845849
H	-2.10811253	-1.62292440	2.24208161
C	-5.89183546	-1.00436042	1.36414658
C	-5.53020661	1.79773925	-2.45434954
H	-3.91143272	2.63940308	-3.62021183
H	-6.94363518	0.83268311	-1.13258415
C	-5.53324043	-1.79743345	2.44908069
H	-3.91591534	-2.63874212	3.61720675
H	-6.94502924	-0.83269891	1.12532849
H	-6.30379670	2.25508656	-3.07593470
H	-6.30759830	-2.25499916	3.06954815
N	-2.70632127	0.00056917	-0.00058454
C	5.34473529	-0.00105994	0.00366880
C	6.05854883	-1.20086565	-0.14619196
C	6.05917929	1.19810475	0.15749218
C	7.44698875	-1.19637561	-0.14336462
H	5.50669167	-2.13491798	-0.26183149
C	7.44741485	1.19237773	0.15932146
H	5.50766858	2.13172531	0.27823157
C	8.16862248	-0.00205908	0.00628742
H	7.98804700	-2.13945749	-0.25742367
H	7.98898385	2.13381977	0.28429621
C	9.66658330	0.00255160	-0.02474816
H	10.02688715	0.16049472	-1.05538717
H	10.08074004	0.81307838	0.59175842
H	10.08158000	-0.95406574	0.32289324
C	3.87607650	-0.00076619	0.00328228
N	3.25396403	-1.18521872	-0.14483713
N	3.25430449	1.18398074	0.15040017
N	1.27204815	-0.00019457	0.00154133

Structure of Au6, S0 state

C	-0.80331569	2.03938940	-0.27146872
C	-2.19750891	2.32712636	-0.30573009
C	-2.63957114	3.64279948	-0.48527691
C	-1.71214472	4.67104435	-0.63129334
C	-0.33531044	4.41200837	-0.60755908
C	0.09653948	3.08452337	-0.42789570
H	-3.70698313	3.87553407	-0.51198591
H	-2.06479952	5.69668481	-0.76679415
H	1.17068132	2.87683086	-0.40199641
C	0.66799916	5.51311985	-0.77903278
H	0.19752269	6.50524774	-0.74020600
H	1.44438874	5.46414518	-0.00000739
H	1.18401981	5.42086896	-1.74885281
C	-3.09864898	1.18351000	-0.15144828
C	-4.50172754	1.14393177	-0.14379630
N	-5.16714738	0.00303690	-0.00004592
H	-5.08491844	2.06114004	-0.25839705
C	-3.10006533	-1.17992943	0.15129352
C	-4.50309647	-1.13865823	0.14368963
H	-5.08739294	-2.05515819	0.25830997
C	-2.20031293	-2.32462695	0.30562600
C	-2.64400904	-3.63974998	0.48516686
C	-0.80575370	-2.03859605	0.27146203
C	-1.71786312	-4.66914258	0.63125639
H	-3.71171169	-3.87115361	0.51183186
C	0.09278372	-3.08484258	0.42795888
C	-0.34071647	-4.41179873	0.60759404
H	-2.07178615	-5.69434500	0.76676038
H	1.16718648	-2.87848353	0.40216552
Au	-0.48410437	0.00021209	0.00002287
N	-2.47314165	0.00141252	-0.00010471
C	0.66127590	-5.51412962	0.77894810
H	1.17915747	-5.42122468	1.74770291
H	0.18931216	-6.50562975	0.74229460
H	1.43636993	-5.46737925	-0.00151735
C	2.32628714	-0.64028619	-0.91124942
C	2.32701086	0.63757597	0.91146858
C	1.96856055	-1.41296467	-2.02631471
C	3.69690428	-0.41701919	-0.58951771
C	1.97010534	1.41066442	2.02651518
C	3.69737326	0.41304846	0.58955492
C	2.98539233	-1.96155513	-2.79934765
H	0.91739336	-1.58198418	-2.27175520

C	4.70219146	-0.97887003	-1.38739165
C	2.98752316	1.95836990	2.79939741
H	0.91910480	1.58070119	2.27197223
C	4.70326201	0.97402797	1.38728872
C	4.34240899	-1.75017861	-2.48752477
H	2.72394864	-2.56928644	-3.66974318
H	5.75563218	-0.81191950	-1.14639015
C	4.34431068	1.74572067	2.48742188
H	2.72675475	2.56641962	3.66977298
H	5.75652554	0.80612225	1.14617489
H	5.11612725	-2.19504105	-3.11793581
H	5.11851409	2.18989703	3.11772111
N	1.51884547	-0.00099456	0.00019321

Structure of Au7, S0 state

C	-1.08665897	1.99927434	0.48305997
C	-2.48098464	2.28230540	0.54394106
C	-2.92315714	3.57374030	0.85238527
C	-1.99575140	4.58181305	1.10280784
C	-0.61892488	4.32593026	1.05642363
C	-0.18686503	3.02304143	0.74514979
H	-3.99054809	3.80358296	0.89796398
H	-2.34851521	5.58899518	1.33911061
H	0.88721877	2.81817994	0.69941080
C	0.38461807	5.40405928	1.33772544
H	-0.08773175	6.39294120	1.41605318
H	0.91405722	5.20510792	2.28392043
H	1.15058232	5.44501992	0.54785924
C	-3.38194065	1.16014808	0.27363205
C	-4.78503176	1.12050516	0.26440454
N	-5.44993180	0.00099896	0.00000340
H	-5.36870648	2.01958428	0.47777102
C	-3.38241343	-1.15901780	-0.27352878
C	-4.78548760	-1.11878638	-0.26436388
H	-5.36952750	-2.01761898	-0.47776716
C	-2.48191166	-2.28154608	-0.54381471
C	-2.92459770	-3.57283334	-0.85213877
C	-1.08747361	-1.99905575	-0.48300619
C	-1.99759166	-4.58128841	-1.10250904
H	-3.99207921	-3.80227223	-0.89764648
C	-0.18808631	-3.02319428	-0.74503845
C	-0.62066363	-4.32594164	-1.05618787
H	-2.35075802	-5.58835202	-1.33871600
H	0.88607781	-2.81873882	-0.69934698

Au	-0.76655128	0.00005337	-0.00000754
N	-2.75604631	0.00043529	0.00006008
C	0.38245359	-5.40448802	-1.33740658
H	1.14830257	-5.44579988	-0.54744568
H	-0.09030381	-6.39316811	-1.41581815
H	0.91209637	-5.20572931	-2.28352709
C	2.04447543	-0.72812410	0.84050966
C	2.04459041	0.72742477	-0.84073810
C	1.69761798	-1.61145405	1.87353493
C	3.41273585	-0.47314837	0.54401338
C	1.69785027	1.61075465	-1.87380169
C	3.41280985	0.47233616	-0.54416807
C	2.71854892	-2.23123008	2.58118349
H	0.64896520	-1.80935336	2.10826548
C	4.42044645	-1.11273671	1.28024604
C	2.71885980	2.23041545	-2.58143225
H	0.64921696	1.80874693	-2.10853815
C	4.42060737	1.11179845	-1.28039481
C	4.08668726	-1.99876346	2.30287092
H	2.45714311	-2.92478565	3.38628320
H	5.47226863	-0.91598916	1.05088147
C	4.08696649	1.99781833	-2.30306465
H	2.45755191	2.92397571	-3.38655957
H	5.47240426	0.91495241	-1.05099756
N	1.23460047	-0.00032591	-0.00015175
C	5.14521454	2.70117021	-3.10741583
H	5.05750127	2.46798344	-4.18122515
H	5.06189042	3.79637507	-3.01302629
H	6.15557866	2.41313842	-2.78328276
C	5.14484439	-2.70224723	3.10722602
H	5.05715950	-2.46904861	4.18103519
H	5.06138642	-3.79744150	3.01283574
H	6.15524395	-2.41433786	2.78309418

Structure of Au1, S1 state

C	1.60434468	-1.16956331	-0.08721366
C	3.55486850	-0.00001002	-0.00906368
C	1.60435124	1.16955403	-0.08721475
N	2.89989748	1.19765727	0.02711127
N	2.89988962	-1.19767535	0.02711295
C	0.70195219	-2.30812822	0.04080867
C	1.10555559	-3.64679414	0.13766479
C	-0.68076307	-2.04040989	0.08701846
C	0.16187137	-4.65058876	0.31205797

H	2.17370973	-3.87365829	0.08661302
C	-1.63021732	-3.02674519	0.29876287
C	-1.21304499	-4.36668083	0.39351397
H	0.49548476	-5.68765651	0.40325116
H	-2.69153200	-2.78138922	0.37149513
C	-2.21908913	-5.46676599	0.55592401
H	-1.84907773	-6.24489906	1.23998471
H	-3.17571493	-5.08792827	0.94146142
H	-2.41998515	-5.95487248	-0.41234741
C	0.70196533	2.30812273	0.04080591
C	1.10557464	3.64678778	0.13765875
C	-0.68075179	2.04041187	0.08701608
C	0.16189527	4.65058712	0.31204875
H	2.17372982	3.87364679	0.08660590
C	-1.63020134	3.02675254	0.29875696
C	-1.21302285	4.36668632	0.39350492
H	0.49551358	5.68765355	0.40323899
H	-2.69151726	2.78140169	0.37148882
C	-2.21906125	5.46677720	0.55591156
H	-1.84904820	6.24490779	1.23997426
H	-2.41995046	5.95488582	-0.41236028
H	-3.17569090	5.08794503	0.94144481
Au	-1.11054385	0.00000190	-0.20924430
C	-3.12012443	0.00000533	-0.14484769
C	-3.85103328	-0.00000607	-1.34418588
C	-3.80022509	0.00001696	1.08308114
C	-5.23961960	-0.00000549	-1.28885620
H	-3.36414327	-0.00001556	-2.32107814
C	-5.18862976	0.00001715	1.08556728
H	-3.27092203	0.00002588	2.03760610
C	-5.93810762	0.00000601	-0.08702443
H	-7.02824580	0.00000623	-0.06358828
F	-5.93363914	-0.00001641	-2.42651954
F	-5.83582502	0.00002825	2.25022161
C	5.00940313	-0.00001649	-0.01147196
C	5.73932993	-1.20648315	-0.01573008
C	5.73933950	1.20645589	-0.01572142
C	7.12827575	-1.19964214	-0.02609472
H	5.19253095	-2.15100704	-0.00691829
C	7.12827432	1.19961221	-0.02608540
H	5.19253918	2.15097930	-0.00690273
C	7.85710332	-0.00002200	-0.03451706
H	7.66694872	-2.15210203	-0.02573705
H	7.66694952	2.15207198	-0.02572093

C	9.35706136	0.00002050	-0.08242026
H	9.77731914	-0.89249396	0.40425776
H	9.71975836	0.00147271	-1.12480808
H	9.77733850	0.89120862	0.40668664
N	0.91999870	-0.00000359	-0.30788076

Structure of Au2, S1 state

C	-2.07856092	1.16743122	-0.09176465
C	-4.02350696	-0.01103068	-0.01422519
C	-2.06726720	-1.17065313	-0.09238507
N	-3.36275602	-1.20552616	0.02234743
N	-3.37429332	1.18981171	0.02303665
C	-1.18127147	2.31002819	0.03848841
C	-1.59175365	3.64652090	0.13624821
C	0.20283078	2.04904919	0.08590369
C	-0.65355969	4.65510045	0.31263822
H	-2.66102703	3.86781749	0.08421228
C	1.14673466	3.04047674	0.29976113
C	0.72267750	4.37815822	0.39529284
H	-0.99262569	5.69034817	0.40443520
H	2.20930260	2.80098579	0.37315686
C	1.72287726	5.48327838	0.55977494
H	1.34816427	6.25883002	1.24421011
H	2.68109460	5.10906814	0.94589157
H	1.92221526	5.97337628	-0.40781418
C	-1.15901126	-2.30458095	0.03740116
C	-1.55653055	-3.64502236	0.13508339
C	0.22253604	-2.03026406	0.08473533
C	-0.60864247	-4.64451857	0.31135117
H	-2.62360103	-3.87675568	0.08317815
C	1.17594722	-3.01261592	0.29830519
C	0.76486247	-4.35431432	0.39390707
H	-0.93776208	-5.68297435	0.40313030
H	2.23616249	-2.76293297	0.37160192
C	1.77578838	-5.44962324	0.55843112
H	1.40944291	-6.22786707	1.24435072
H	1.97855957	-5.93904331	-0.40878401
H	2.73084671	-5.06571272	0.94284644
Au	0.64442635	0.01153281	-0.21360772
C	2.65352705	0.01970374	-0.14609552
C	3.39619315	0.03294279	-1.33907058
C	3.34182041	0.01460697	1.07898001
C	4.78864013	0.03785662	-1.30941586
H	2.88700049	0.04215897	-2.30582624

C	4.73264343	0.01969255	1.11256604
H	2.78704626	0.00930426	2.02029372
C	5.45606316	0.02931995	-0.08341429
C	-5.47803698	-0.01793762	-0.01699989
C	-6.21368702	1.18516260	-0.02530854
C	-6.20225902	-1.22770812	-0.02699547
C	-7.60244467	1.17180025	-0.04046207
H	-5.67125625	2.13225925	-0.02265846
C	-7.59129342	-1.22738882	-0.04212368
H	-5.65102359	-2.16967815	-0.02575117
C	-8.32574054	-0.03131948	-0.04671339
H	-8.14549574	2.12173171	-0.05022929
H	-8.12536457	-2.18236589	-0.05319747
C	-9.82637725	-0.03748170	-0.02984523
H	-10.21080301	-0.01310372	1.00442886
H	-10.24019780	0.84025727	-0.54809111
H	-10.23257003	-0.94280887	-0.50449405
H	5.25888080	0.02193129	2.06957561
H	5.35746554	0.05369429	-2.24133811
C	6.95664453	-0.02048462	-0.04450197
F	7.40580681	-1.28292907	0.02505368
F	7.45010280	0.62259073	1.01966543
F	7.50618675	0.52597991	-1.13393608
N	-1.38895955	0.00178623	-0.31430471

Structure of Au3, S1 state

C	1.48945763	1.17493735	0.01500958
C	3.44138011	-0.00106160	0.00589181
C	1.48909938	-1.17644411	0.01516718
N	2.79033610	-1.20354359	0.00302887
N	2.79068036	1.20174998	0.00286149
C	0.59539327	2.32771293	0.00269562
C	1.02324597	3.66348959	-0.00823178
C	-0.79026197	2.06981793	-0.00124996
C	0.09527536	4.69436631	-0.02476763
H	2.09756261	3.86626216	-0.00485543
C	-1.72329100	3.09921067	-0.02008492
C	-1.28692718	4.43511321	-0.03062569
H	0.44301902	5.73099666	-0.03457085
H	-2.79342927	2.88425114	-0.02553879
C	-2.27536714	5.56395928	-0.04341173
H	-2.10205591	6.22968913	-0.90372342
H	-3.30985407	5.19804715	-0.09603242
H	-2.17769154	6.18140944	0.86408895

C	0.59450782	-2.32866312	0.00293796
C	1.02120896	-3.66483063	-0.00789254
C	-0.79094542	-2.06975698	-0.00109364
C	0.09233802	-4.69488793	-0.02444411
H	2.09535019	-3.86854932	-0.00442982
C	-1.72493118	-3.09822700	-0.01999361
C	-1.28966840	-4.43451474	-0.03044839
H	0.43920157	-5.73181535	-0.03415136
H	-2.79487139	-2.88228154	-0.02554678
C	-2.27902104	-5.56255902	-0.04341415
H	-2.10707728	-6.22751950	-0.90460521
H	-2.18095102	-6.18104991	0.86332893
H	-3.31326482	-5.19577605	-0.09468680
Au	-1.23684389	0.00017666	0.01993290
C	-3.21504614	0.00282757	0.00855225
C	-3.94950848	0.00671554	1.21882799
C	-3.93877516	0.00513458	-1.20832300
C	-5.33501758	0.01143392	1.20872889
H	-3.42027753	0.00890150	2.17467457
C	-5.32379013	0.00985408	-1.21044222
H	-3.40075119	0.00605100	-2.15923981
C	-6.05214864	0.01080551	-0.00378646
C	4.89537841	-0.00123452	0.00363971
C	5.62786563	1.20488870	0.00498710
C	5.62761533	-1.20752474	0.00521882
C	7.01681367	1.19769906	0.00508629
H	5.08148128	2.14961353	0.00925417
C	7.01654543	-1.20063519	0.00532045
H	5.08102332	-2.15213078	0.00966854
C	7.74663773	-0.00153627	0.00241665
H	7.55525524	2.15044517	0.00949350
H	7.55478448	-2.15349567	0.00991872
C	9.24715972	-0.00175972	-0.03295792
H	9.62036645	-0.00373283	-1.07174660
H	9.66355022	0.89070190	0.45737912
H	9.66335707	-0.89254022	0.46061368
H	-5.86515117	0.01503984	-2.16028269
H	-5.88462299	0.01787411	2.15376743
C	-7.54524643	-0.01830022	-0.01287513
N	0.78871757	-0.00058229	0.03745161
H	-7.95483319	0.50894736	-0.88631589
H	-7.90109588	-1.06200574	-0.07020654
H	-7.96635412	0.41915924	0.90302770

Structure of Au4, S1 state

C	2.66227099	1.13241269	-0.04868831
C	2.65134133	-1.21683066	-0.04531998
N	3.99538645	-1.21355074	-0.04407595
N	3.96932670	1.18535599	-0.02896786
C	1.76888268	2.30211114	0.00723325
C	2.20622976	3.62593168	0.03969317
C	0.38578452	2.06123027	0.04673759
C	1.28052163	4.66429512	0.12523970
H	3.28146713	3.81941006	0.00566847
C	-0.54567149	3.07431251	0.15309668
C	-0.09547575	4.41248504	0.18031642
H	1.63492743	5.69748164	0.15892040
H	-1.61483908	2.86082381	0.20524915
C	-1.09534025	5.52794051	0.24853487
H	-1.58997783	5.66281969	-0.72729755
H	-1.88484422	5.31130520	0.98327663
H	-0.61756987	6.47997122	0.51725643
C	1.77540575	-2.33607478	0.02760547
C	2.14637234	-3.70169544	0.04880112
C	0.37722359	-2.08867096	0.07861127
C	1.18739470	-4.69923989	0.14596658
H	3.21046006	-3.94478072	-0.01588857
C	-0.58789980	-3.06104611	0.18692051
C	-0.18802863	-4.41861476	0.21933318
H	1.51295889	-5.74330802	0.16939241
H	-1.64855722	-2.80642967	0.24221493
C	-1.21907567	-5.49957820	0.35929585
H	-0.78512820	-6.49007845	0.16103043
H	-1.64466305	-5.52037920	1.37707452
H	-2.05988056	-5.34933146	-0.33585114
Au	-0.06191145	-0.00505082	-0.08721065
C	-2.06536649	0.02641789	-0.07369514
C	-2.76932216	0.11226990	1.13902396
C	-2.77370088	-0.04388001	-1.28515382
C	-4.15725390	0.11734769	1.11449763
H	-2.25852767	0.17312948	2.10151998
C	-4.16232177	-0.03723322	-1.25675883
H	-2.26706295	-0.10696192	-2.24974634
C	-4.88348973	0.04299973	-0.07038168
H	-5.97389265	0.04876636	-0.06837903
F	-4.82717382	0.19562788	2.26330507
F	-4.83538853	-0.10832846	-2.40435729
C	6.09343669	0.00162053	-0.08685541

H	6.47387531	0.55119416	0.78934619
H	6.43820951	0.55330447	-0.97654355
H	6.51644259	-1.00987000	-0.09731772
C	4.60308567	-0.04632499	-0.05800773
N	1.97252250	-0.01278543	-0.10654078

Structure of Au5, S1 state

Au	-0.69723896	-0.00013457	0.00258724
C	-0.34545649	-2.04314842	-0.26401394
C	1.04591785	-2.32661019	-0.29530200
C	1.49019351	-3.64736325	-0.45160657
C	0.56782896	-4.67634184	-0.57683619
C	-0.81656274	-4.42028255	-0.55081680
C	-1.25086691	-3.09569855	-0.39323267
H	2.56450591	-3.84926711	-0.46850580
H	0.91885455	-5.70525901	-0.69672542
H	-2.32724574	-2.89700473	-0.37068283
C	-1.79818709	-5.54561006	-0.70021518
H	-1.59806853	-6.34767633	0.02777405
H	-2.83247660	-5.20265452	-0.55724045
H	-1.72798574	-6.00067949	-1.70190636
C	1.93786361	-1.18324151	-0.14848361
C	1.93824719	1.18171373	0.15629477
C	1.04668802	2.32575529	0.30027384
C	1.49145834	3.64653908	0.45486690
C	-0.34478675	2.04296035	0.26765049
C	0.56947745	4.67620487	0.57722377
H	2.56585385	3.84790897	0.47276516
C	-1.24980788	3.09618372	0.39408633
C	-0.81500976	4.42080932	0.54995884
H	0.92088742	5.70514122	0.69581269
H	-2.32626071	2.89802268	0.37048104
C	-1.79623154	5.54690030	0.69621003
H	-1.72722609	6.00344693	1.69731258
H	-1.59461737	6.34775079	-0.03270278
H	-2.83050218	5.20429399	0.55226746
C	-3.51383958	0.57375359	-0.95596298
C	-3.51749690	-0.57229500	0.95172110
C	-3.12378021	1.27691299	-2.10356452
C	-4.88523231	0.37786167	-0.62822854
C	-3.13184456	-1.27571677	2.10063421
C	-4.88761817	-0.37577887	0.61910607
C	-4.12994067	1.77835467	-2.92755534
H	-2.06767897	1.42969252	-2.33277956

C	-5.87067827	0.88503530	-1.45681498
C	-4.14116957	-1.77675952	2.92100241
H	-2.07663278	-1.42891851	2.33368275
C	-5.87624696	-0.88259161	1.44411880
C	-5.47964997	1.58727925	-2.61150373
H	-3.86015526	2.33074020	-3.82944252
H	-6.92879398	0.74919200	-1.22491094
C	-5.48965655	-1.58506982	2.60015227
H	-3.87484827	-2.32930947	3.82381868
H	-6.93346820	-0.74630700	1.20842007
H	-6.24727284	1.99379673	-3.27296860
H	-6.25981069	-1.99129305	3.25884885
N	-2.70964859	0.00055675	-0.00069816
C	5.34156975	-0.00113169	0.00287939
C	6.07364620	-1.19773720	-0.14806483
C	6.07391719	1.19473446	0.15833361
C	7.46264007	-1.19130724	-0.14541997
H	5.52683485	-2.13504424	-0.26416541
C	7.46290858	1.18752189	0.15926219
H	5.52733312	2.13121095	0.28198438
C	8.19264305	-0.00174085	0.00507825
H	8.00088166	-2.13708210	-0.26131276
H	8.00131569	2.13187660	0.28554998
C	9.69320779	0.00234600	-0.02801308
H	10.06769211	0.13443137	-1.05788050
H	10.10888517	0.82404973	0.57389418
H	10.10873758	-0.94473913	0.34700253
C	3.88647272	-0.00097334	0.00308291
N	3.24000279	-1.19506402	-0.15038470
N	3.24037914	1.19344661	0.15566162
N	1.24632896	-0.00101055	0.00676553

Structure of Au6, S1 state

C	0.82030969	2.04044599	0.25775586
C	2.21278897	2.33390301	0.29113466
C	2.62268017	3.66968079	0.44212639
C	1.68203900	4.68459562	0.55761671
C	0.30226807	4.41163983	0.52699086
C	-0.10378449	3.07852465	0.37473419
H	3.68727443	3.91836643	0.46487195
H	2.02088160	5.71827830	0.67301959
H	-1.17605370	2.85787818	0.34690899
C	-0.69916449	5.52161367	0.66631927
H	-0.50965071	6.32571786	-0.06251954

H	-1.72625504	5.15989732	0.51577527
H	-0.64771611	5.98128837	1.66724633
C	3.10828346	1.19514261	0.15168567
C	4.49077570	1.13934786	0.14331842
N	5.19235056	-0.00310994	-0.00117609
H	5.06643079	2.06315110	0.25859169
C	3.10673637	-1.19899796	-0.15143609
C	4.48930287	-1.14474808	-0.14494780
H	5.06376540	-2.06915430	-0.26134923
C	2.20978416	-2.33661903	-0.29079962
C	2.61795506	-3.67293531	-0.44161690
C	0.81767337	-2.04136150	-0.25752131
C	1.67600534	-4.68663792	-0.55720243
H	3.68222620	-3.92302594	-0.46401798
C	-0.10774513	-3.07824321	-0.37451310
C	0.29659549	-4.41188482	-0.52680662
H	2.01352580	-5.72077401	-0.67241224
H	-1.17973040	-2.85622216	-0.34667328
Au	0.48763335	-0.00026586	0.00030832
N	2.43532542	-0.00160197	0.00099053
C	-0.70632388	-5.52041799	-0.66690617
H	-0.65945993	-5.97551897	-1.67017336
H	-0.51446802	-6.32799454	0.05742434
H	-1.73249272	-5.15851038	-0.51061143
C	-2.33053442	-0.51519407	0.98498580
C	-2.32964458	0.51800076	-0.98511214
C	-1.94272662	-1.15233101	2.17083318
C	-3.70119237	-0.33721085	0.64448814
C	-1.94074536	1.15476055	-2.17081049
C	-3.70061220	0.34145420	-0.64509878
C	-2.95057299	-1.60224787	3.02227172
H	-0.88675870	-1.29445394	2.40741384
C	-4.68843868	-0.79284538	1.50064345
C	-2.94781336	1.60574724	-3.02259919
H	-0.88454277	1.29583199	-2.40697105
C	-4.68707117	0.79811577	-1.50161244
C	-4.29976175	-1.42654081	2.69490690
H	-2.68267039	-2.10212507	3.95489402
H	-5.74607879	-0.66857470	1.25995809
C	-4.29730252	1.43142774	-2.69572509
H	-2.67905883	2.10538037	-3.95510690
H	-5.74492783	0.67492967	-1.26132252
H	-5.06863892	-1.79183549	3.37865576
H	-5.06555435	1.79752785	-3.37974631

N	-1.52362795	0.00097455	0.00006674

Structure of Au7, S1 state

C	-1.09880937	2.00028598	0.47781079
C	-2.49185909	2.29086718	0.54012270
C	-2.90271504	3.60331784	0.82943760
C	-1.96263742	4.60105200	1.05126967
C	-0.58282824	4.33243444	0.99432597
C	-0.17680632	3.02232556	0.70476278
H	-3.96743755	3.84825557	0.87611719
H	-2.30183901	5.61729764	1.27239909
H	0.89562687	2.80590590	0.65471448
C	0.41858234	5.42171157	1.24969142
H	0.21785819	6.30563045	0.62360290
H	0.38093597	5.76004864	2.29856469
H	1.44407902	5.08337513	1.04370904
C	-3.38891178	1.17413845	0.27952866
C	-4.77156114	1.12022254	0.26606051
N	-5.47374435	-0.00001054	0.00025931
H	-5.34686157	2.02636222	0.48065258
C	-3.38893536	-1.17405485	-0.27963091
C	-4.77158339	-1.12021782	-0.26571043
H	-5.34690290	-2.02641102	-0.48002554
C	-2.49190426	-2.29080019	-0.54023039
C	-2.90278742	-3.60323727	-0.82956986
C	-1.09884865	-2.00025761	-0.47787693
C	-1.96273143	-4.60100210	-1.05135217
H	-3.96751553	-3.84813572	-0.87632626
C	-0.17686711	-3.02232343	-0.70480061
C	-0.58291625	-4.33242627	-0.99435029
H	-2.30195468	-5.61723594	-1.27250280
H	0.89557057	-2.80593278	-0.65472990
Au	-0.77062581	0.00001957	-0.00007073
N	-2.71779537	0.00008606	-0.00026563
C	0.41846905	-5.42174877	-1.24962094
H	1.44399527	-5.08334407	-1.04389955
H	0.21787206	-6.30551139	-0.62326852
H	0.38063931	-5.76035752	-2.29839856
C	2.05082662	-0.64148393	0.90657548
C	2.05089423	0.64146610	-0.90651395
C	1.67499337	-1.43192524	1.99928852
C	3.42319009	-0.42196838	0.59458437
C	1.67514230	1.43198408	-1.99919878
C	3.42323361	0.42190985	-0.59445179

C	2.68571070	-1.98850852	2.77821233
H	0.62178737	-1.61011930	2.22355133
C	4.40934751	-0.98596160	1.37930880
C	2.68591674	1.98857850	-2.77804008
H	0.62195394	1.61024124	-2.22349549
C	4.40945024	0.98593049	-1.37908389
C	4.04642947	-1.78230333	2.49174390
H	2.41470563	-2.60808398	3.63588323
H	5.46584797	-0.82660589	1.14999509
C	4.04661548	1.78230739	-2.49151979
H	2.41497203	2.60823847	-3.63566898
H	5.46593285	0.82659125	-1.14967701
N	1.24554504	-0.00000979	0.00000194
C	5.11654670	2.39281753	-3.34079401
H	5.76232818	1.61203899	-3.77385559
H	4.69731808	2.98905159	-4.16171023
H	5.76997807	3.04319599	-2.73746496
C	5.11629921	-2.39318324	3.34082851
H	5.76510324	-1.61300336	3.77037362
H	4.69700036	-2.98603985	4.16415118
H	5.76673543	-3.04713984	2.73808461

Structure of Au1, T1 state

C	-1.57236397	-1.18329049	-0.04083057
C	-3.52135831	-0.00234565	0.00055576
C	-1.57248371	1.17999253	0.04178063
N	-2.88620658	1.21052248	0.04299015
N	-2.88529844	-1.21433976	-0.04184576
C	-0.66581680	-2.33468033	-0.08452059
C	-1.13811687	-3.65201662	-0.12531026
C	0.73377753	-2.05908346	-0.08021905
C	-0.23616772	-4.70768568	-0.16587780
H	-2.21578364	-3.83492261	-0.12127609
C	1.60721734	-3.14341194	-0.12428844
C	1.14851443	-4.47110263	-0.16935734
H	-0.60636469	-5.73613809	-0.19369353
H	2.68671813	-2.96274480	-0.11952692
C	2.11686219	-5.61403569	-0.24839173
H	2.26933095	-5.91929876	-1.29752175
H	3.10037253	-5.33962783	0.15889430
H	1.74584253	-6.49643535	0.29341428
C	-0.66748701	2.33267916	0.08526780
C	-1.14144948	3.64929666	0.12608777
C	0.73234260	2.05849926	0.08060668

C	-0.24064984	4.70608154	0.16636267
H	-2.21932915	3.83092622	0.12228298
C	1.60449185	3.14374353	0.12436072
C	1.14415917	4.47099866	0.16949817
H	-0.61199707	5.73410615	0.19417589
H	2.68421658	2.96439139	0.11924153
C	2.11162350	5.61467398	0.24848319
H	1.73428519	6.50106989	-0.28221644
H	2.27420829	5.91102126	1.29866032
H	3.09151719	5.34491272	-0.17053362
Au	1.10542095	-0.00024688	0.00013960
C	3.12040065	0.00056755	-0.00023135
C	3.83370937	-0.69434538	0.98798495
C	3.83266159	0.69622622	-0.98868389
C	5.22369923	-0.67367296	0.97050078
H	3.33021614	-1.25732285	1.77615476
C	5.22267558	0.67699521	-0.97166618
H	3.32832713	1.25870172	-1.77667575
C	5.95228089	0.00203873	-0.00070373
H	7.04248913	0.00260935	-0.00088195
F	5.89110441	-1.33280448	1.92182062
F	5.88907898	1.33682929	-1.92320149
C	-4.94490514	-0.00089949	0.00042111
C	-5.69662951	-1.24432803	-0.04371242
C	-5.69692632	1.25194241	0.04445662
C	-7.05901756	-1.22625545	-0.04402150
H	-5.13940369	-2.18025537	-0.07644003
C	-7.06268703	1.23967637	0.04301398
H	-5.13526644	2.18490532	0.07800289
C	-7.80990071	0.01334296	-0.00107324
H	-7.61264117	-2.16858768	-0.07767128
H	-7.61120012	2.18478929	0.07617934
C	-9.29493548	-0.01191224	-0.00143947
H	-9.68066518	-0.53462387	-0.89478709
H	-9.67998217	-0.57521731	0.86745383
H	-9.72551581	0.99802772	0.02268058
N	-0.91140971	-0.00091679	0.00045045

Structure of Au2, T1 state

C	-2.04612519	1.18227024	0.01260286
C	-3.99084642	-0.00627282	-0.00289548
C	-2.03756300	-1.18196105	-0.01138143
N	-3.35129077	-1.21765319	-0.01410120
N	-3.35928535	1.20886504	0.01067619

C	-1.14330413	2.33765883	0.02913048
C	-1.62069235	3.65380307	0.03706278
C	0.25754492	2.06745826	0.03352559
C	-0.72308649	4.71381177	0.05322828
H	-2.69905206	3.83235531	0.02701085
C	1.12636058	3.15634267	0.05247631
C	0.66250514	4.48293370	0.06460580
H	-1.09742723	5.74114130	0.05555807
H	2.20672166	2.98065891	0.05421047
C	1.62613680	5.63135232	0.11753957
H	1.77243264	5.96520573	1.15880725
H	2.61259873	5.35011167	-0.27775741
H	1.25426939	6.49751583	-0.44933437
C	-1.12775590	-2.33198576	-0.02437568
C	-1.59696875	-3.65094826	-0.03337073
C	0.27129957	-2.05284201	-0.02383644
C	-0.69257163	-4.70534534	-0.04566229
H	-2.67421755	-3.83628046	-0.02712430
C	1.14700568	-3.13610343	-0.03885724
C	0.69144241	-4.46566642	-0.05198678
H	-1.06039075	-5.73501618	-0.04879226
H	2.22624198	-2.95356863	-0.03647480
C	1.66284102	-5.60769064	-0.10087419
H	1.28995218	-6.47991592	0.45585704
H	1.82374250	-5.93343657	-1.14256396
H	2.64280419	-5.32271024	0.30774613
Au	0.63788500	0.00858612	0.00525576
C	2.65183292	0.01450736	0.00822595
C	3.37328657	0.68831069	-0.99039043
C	3.37432099	-0.65224205	1.01065326
C	4.76675124	0.68898583	-0.99514387
H	2.84351403	1.22821802	-1.77918960
C	4.76787222	-0.64311088	1.02048968
H	2.84547958	-1.18912287	1.80215932
C	5.46702623	0.02445363	0.01341453
C	-5.41431135	-0.01278546	-0.00556066
C	-6.17056496	1.22871401	0.00591957
C	-6.16196487	-1.26893290	-0.01993195
C	-7.53290247	1.20584311	0.00333633
H	-5.61667871	2.16713782	0.01668590
C	-7.52777311	-1.26140695	-0.02214181
H	-5.59705434	-2.20048362	-0.02878926
C	-8.27933916	-0.03701079	-0.01067856
H	-8.08986810	2.14677235	0.01214089

H	-8.07296714	-2.20896031	-0.03296179
C	-9.76448685	-0.01709119	-0.01357122
H	-10.15376294	0.51947621	0.87003894
H	-10.15001087	0.52971881	-0.89264008
H	-10.19138475	-1.02886740	-0.02065392
H	5.31311668	-1.15631104	1.81586105
H	5.31102437	1.21802503	-1.78082567
C	6.96506347	-0.01548291	-0.01536242
F	7.42437961	-1.05768908	-0.72857863
F	7.49049469	-0.13173187	1.21046563
F	7.48783425	1.08511058	-0.57068486
N	-1.38122015	0.00184903	0.00169190

Structure of Au3, T1 state

C	1.47049030	1.18316411	0.01299085
C	3.41928835	0.00080421	-0.00148396
C	1.46905014	-1.18042517	-0.01269865
N	2.78317114	-1.21271064	-0.01544815
N	2.78383563	1.21427271	0.01298960
C	0.56373272	2.33578174	0.02898228
C	1.03864557	3.65303318	0.04173860
C	-0.83686822	2.06197028	0.02952190
C	0.13935079	4.71142812	0.05960390
H	2.11673989	3.83351583	0.03445840
C	-1.70695282	3.15018119	0.05081935
C	-1.24587727	4.47751646	0.06820300
H	0.51174476	5.73949302	0.06590435
H	-2.78676351	2.97138842	0.05043515
C	-2.21164246	5.62416604	0.12370301
H	-1.84537547	6.48918996	-0.44867967
H	-3.20006343	5.33957369	-0.26422707
H	-2.35170354	5.96176375	1.16463063
C	0.56239595	-2.33320021	-0.02772875
C	1.03746349	-3.65041511	-0.04851906
C	-0.83786455	-2.05912213	-0.02729301
C	0.13819331	-4.70855006	-0.06880574
H	2.11560788	-3.83072017	-0.05125423
C	-1.70817667	-3.14731851	-0.05131469
C	-1.24720013	-4.47451495	-0.06844381
H	0.51047410	-5.73651206	-0.08801372
H	-2.78792739	-2.96830905	-0.05741967
C	-2.21183194	-5.62344525	-0.06147416
H	-1.86323611	-6.44679620	-0.70261405
H	-2.31881778	-6.03250590	0.95742499

H	-3.21130019	-5.31513862	-0.39929426
Au	-1.21362113	0.00162048	0.00020714
C	-3.22961977	0.00147855	-0.00311700
C	-3.95803947	-0.69770299	0.97115509
C	-3.95487592	0.70414052	-0.97726062
C	-5.35392387	-0.68706056	0.97326206
H	-3.43284924	-1.26042016	1.74807622
C	-5.35077733	0.69762448	-0.98096846
H	-3.42723567	1.27213777	-1.74864259
C	-6.07870429	0.00399398	-0.00592505
C	4.84242330	-0.00149898	-0.00245064
C	5.59542622	1.24199317	0.01280193
C	5.59405983	-1.25503020	-0.01852214
C	6.95808775	1.22308245	0.01252987
H	5.03895595	2.17888086	0.02484744
C	6.96009735	-1.24335506	-0.01839045
H	5.03216170	-2.18838693	-0.03067473
C	7.70792101	-0.01705181	-0.00306309
H	7.51209099	2.16575706	0.02463216
H	7.50808173	-2.18931825	-0.03054003
C	9.19331409	0.00700246	-0.00357249
H	9.57900156	0.55831592	-0.87966092
H	9.57985375	0.54063118	0.88305052
H	9.62288342	-1.00363793	-0.01384340
H	-5.88656311	1.25194174	-1.75808759
H	-5.89219769	-1.23238813	1.75500133
C	-7.58058356	-0.02159920	-0.02650684
N	0.80933931	0.00104670	0.00147508
H	-7.99428743	0.89595468	-0.47019665
H	-7.95288122	-0.86902100	-0.62688377
H	-7.99675858	-0.13409345	0.98545166

Structure of Au4, T1 state

C	2.65746800	1.09412991	0.04436198
C	2.56026945	-1.25278083	-0.04183556
N	3.88664306	-1.33235256	-0.04395008
N	4.00628581	1.05627698	0.04086853
C	1.84092497	2.25647288	0.08860542
C	2.38054317	3.58847556	0.13482718
C	0.35136470	2.04102045	0.08509066
C	1.52836076	4.65140944	0.18310088
H	3.46431957	3.71916674	0.13192877
C	-0.44919848	3.15589359	0.14055504
C	0.07566235	4.48127516	0.18882514

H	1.92945961	5.66812585	0.21969249
H	-1.53706666	3.03406631	0.14256591
C	-0.79571640	5.68458347	0.24131529
H	-1.86164535	5.41938954	0.23656381
H	-0.59077711	6.28434001	1.14642632
H	-0.60024365	6.35435503	-0.61544097
C	1.62752483	-2.38360467	-0.08869704
C	2.06750323	-3.70894723	-0.13351270
C	0.23479308	-2.06894941	-0.08501541
C	1.13685559	-4.74300394	-0.17807445
H	3.14054820	-3.91688633	-0.13085931
C	-0.66437648	-3.12733124	-0.13275342
C	-0.23778442	-4.46931412	-0.18048943
H	1.47985360	-5.78044901	-0.21058937
H	-1.73937838	-2.92086590	-0.12991636
C	-1.24777605	-5.57596160	-0.25043782
H	-0.78879319	-6.55998964	-0.08191628
H	-2.04735539	-5.43146918	0.49215350
H	-1.73278507	-5.59576119	-1.24047049
Au	-0.07073591	0.00313562	-0.00025843
C	-2.08494957	0.06818549	-0.00110631
C	-2.81921595	-0.61424691	0.98013324
C	-2.77440917	0.79776312	-0.98090484
C	-4.20784203	-0.54868972	0.96403617
H	-2.33345947	-1.20156190	1.76160871
C	-4.16443847	0.82409222	-0.96198104
H	-2.25225869	1.35139568	-1.76346199
C	-4.91480930	0.16184247	0.00171829
H	-6.00442485	0.19767908	0.00271669
F	-4.89551778	-1.19694054	1.90828280
F	-4.80953904	1.51647011	-1.90495362
C	6.04777785	-0.24544609	-0.00628996
H	6.50540983	0.74961625	0.02707701
H	6.38277126	-0.78023082	-0.90842133
H	6.38358746	-0.83946860	0.85772212
C	4.56010970	-0.14693309	-0.00210073
N	1.94764810	-0.06600189	0.00263164

Structure of Au5, T1 state

Au	-0.69823708	-0.00094608	-0.05857309
C	-0.33551821	-2.04639302	-0.30413592
C	1.05711910	-2.32140741	-0.33355314
C	1.50939579	-3.64233539	-0.46884567
C	0.59356130	-4.67846924	-0.57695229

C	-0.79261140	-4.42989847	-0.55701958
C	-1.23474993	-3.10564148	-0.42267538
H	2.58493167	-3.83756570	-0.48427608
H	0.95058717	-5.70714161	-0.67995593
H	-2.31226723	-2.91340390	-0.40588528
C	-1.76743504	-5.56373755	-0.68466239
H	-1.57439172	-6.34109469	0.07165212
H	-2.80499723	-5.22120606	-0.56667189
H	-1.68090758	-6.05049226	-1.66994326
C	1.94175602	-1.17133698	-0.20727602
C	1.93174401	1.19052659	0.10082475
C	1.03719758	2.32618361	0.27865546
C	1.47788565	3.64405654	0.46916822
C	-0.35289357	2.03873182	0.24933619
C	0.55295022	4.66549317	0.62916691
H	2.55159246	3.84927189	0.48369353
C	-1.26142721	3.08352536	0.41567444
C	-0.83083531	4.40467276	0.60667039
H	0.90100241	5.69211928	0.77454978
H	-2.33718395	2.88167837	0.39841484
C	-1.81571046	5.52097892	0.79660345
H	-1.74445028	5.94222107	1.81294275
H	-1.62007875	6.34773904	0.09557817
H	-2.84923226	5.17946841	0.64479548
C	-3.54080385	0.61605550	-0.90590255
C	-3.49036976	-0.63439384	0.93596379
C	-3.18537944	1.38040488	-2.02483345
C	-4.90154427	0.40164848	-0.54912821
C	-3.07441274	-1.39447905	2.03710003
C	-4.86868254	-0.41887532	0.65472384
C	-4.21559668	1.92641468	-2.78893897
H	-2.13695092	1.54546542	-2.27933683
C	-5.91160558	0.95186435	-1.31919666
C	-4.06125736	-1.93654195	2.85885921
H	-2.01362423	-1.55849582	2.23378532
C	-5.83505427	-0.96561217	1.48115270
C	-5.55542396	1.71718149	-2.44405368
H	-3.97214549	2.52759990	-3.66682537
H	-6.96228074	0.80095176	-1.06391060
C	-5.41793446	-1.72752348	2.58733269
H	-3.77025196	-2.53422382	3.72457817
H	-6.89810779	-0.81385310	1.28432247
H	-6.34217665	2.15832029	-3.05926337
H	-6.16996265	-2.16557840	3.24660987

N	-2.70830777	-0.01060560	-0.00768148
C	5.34031195	0.01468947	0.02099521
C	6.07713904	-1.17918801	-0.12471217
C	6.06629858	1.21284947	0.18430471
C	7.46607480	-1.16794198	-0.11054399
H	5.53440187	-2.11821904	-0.24608452
C	7.45536087	1.21074861	0.19676922
H	5.51514437	2.14707634	0.30466006
C	8.19027525	0.02422610	0.04713224
H	8.00871492	-2.11159265	-0.22287036
H	7.98937957	2.15679187	0.32859858
C	9.69099758	0.03353350	0.02576900
H	10.07250974	0.15923324	-1.00227810
H	10.09898829	0.86117303	0.62469116
H	10.10694605	-0.90933906	0.41078169
C	3.88444869	0.01048791	0.00343889
N	3.24304801	-1.18471492	-0.14897713
N	3.23275100	1.19966026	0.16180408
N	1.24621134	0.01921018	-0.14919480

Structure of Au6, T1 state

C	0.82057812	2.03893646	0.28544205
C	2.21300317	2.33154881	0.31609601
C	2.62343526	3.66571605	0.48296270
C	1.68384624	4.67872819	0.61735988
C	0.30378699	4.40536402	0.59384155
C	-0.10297907	3.07436288	0.42777918
H	3.68820004	3.91381011	0.50258698
H	2.02319237	5.71096132	0.74350514
H	-1.17537255	2.85467944	0.40816319
C	-0.69741306	5.51256955	0.75462558
H	-0.51178250	6.32717188	0.03653691
H	-1.72503179	5.15250426	0.60370983
H	-0.64119636	5.95767384	1.76185450
C	3.10715118	1.19533988	0.16053226
C	4.48979285	1.14033339	0.15279296
N	5.19198383	-0.00026146	0.00001261
H	5.06469070	2.06344106	0.27694708
C	3.10703081	-1.19564323	-0.16057437
C	4.48967819	-1.14078322	-0.15278227
H	5.06448274	-2.06395465	-0.27689501
C	2.21276826	-2.33176503	-0.31612247
C	2.62305787	-3.66598174	-0.48288694
C	0.82036677	-2.03900676	-0.28546862

C	1.68335758	-4.67891049	-0.61721722
H	3.68779501	-3.91419991	-0.50243252
C	-0.10329250	-3.07434046	-0.42773012
C	0.30333519	-4.40539879	-0.59374966
H	2.02260079	-5.71119262	-0.74323338
H	-1.17566440	-2.85455275	-0.40805973
Au	0.48586136	-0.00001625	-0.00002078
N	2.43349742	-0.00011365	-0.00005177
C	-0.69803023	-5.51239429	-0.75495507
H	-0.64514433	-5.95385128	-1.76398977
H	-0.50968276	-6.32948613	-0.04044558
H	-1.72525850	-5.15318873	-0.59937553
C	-2.33011079	-0.61528827	0.92771836
C	-2.33009004	0.61550343	-0.92766262
C	-1.94467938	-1.36724500	2.04507798
C	-3.70008133	-0.40378336	0.60638130
C	-1.94463356	1.36742665	-2.04503599
C	-3.70006751	0.40410247	-0.60628706
C	-2.95374490	-1.90309879	2.84389291
H	-0.88948425	-1.53038776	2.27100262
C	-4.68894729	-0.94454187	1.41034179
C	-2.95368112	1.90335143	-2.84382583
H	-0.88943262	1.53049364	-2.27098825
C	-4.68891545	0.94493085	-1.41022285
C	-4.30253210	-1.69685568	2.53372348
H	-2.68645576	-2.49422389	3.72174191
H	-5.74619590	-0.79567816	1.18224116
C	-4.30247514	1.69721011	-2.53361890
H	-2.68637204	2.49445447	-3.72168364
H	-5.74616870	0.79614433	-1.18209346
H	-5.07227369	-2.13022207	3.17548067
H	-5.07220183	2.13062995	-3.17535784
N	-1.52167852	0.00007997	0.00001895

Structure of Au7, T1 state

C	-1.09949760	1.99910163	0.48753558
C	-2.49268511	2.28871181	0.54733216
C	-2.90468218	3.59872711	0.84748933
C	-1.96598097	4.59387825	1.08408631
C	-0.58576881	4.32480717	1.03408214
C	-0.17849676	3.01766727	0.73448467
H	-3.96966953	3.84281234	0.89217887
H	-2.30610718	5.60803349	1.31320519
H	0.89420375	2.80183708	0.69152497

C	0.41474084	5.41062690	1.30716535
H	0.21735431	6.30199014	0.69067017
H	0.37221930	5.73643464	2.35982805
H	1.44109915	5.07432980	1.10206916
C	-3.38799996	1.17350701	0.27913326
C	-4.77056419	1.11928114	0.26547746
N	-5.47280480	-0.00080962	-0.00018030
H	-5.34584291	2.02522118	0.48099473
C	-3.38760303	-1.17448888	-0.27922167
C	-4.77018704	-1.12068043	-0.26576199
H	-5.34516191	-2.02678956	-0.48138262
C	-2.49191368	-2.28940187	-0.54737753
C	-2.90346697	-3.59954565	-0.84756086
C	-1.09881782	-1.99933142	-0.48752450
C	-1.96442889	-4.59438180	-1.08416476
H	-3.96836968	-3.84399394	-0.89226423
C	-0.17748018	-3.01758195	-0.73448497
C	-0.58431363	-4.32485197	-1.03413062
H	-2.30422238	-5.60864268	-1.31330953
H	0.89514832	-2.80140541	-0.69147247
Au	-0.76886178	-0.00006870	0.00003463
N	-2.71520044	-0.00039570	0.00003815
C	0.41657899	-5.41029701	-1.30730996
H	1.44275012	-5.07388835	-1.10146449
H	0.21905239	-6.30207809	-0.69147090
H	0.37471518	-5.73546339	-2.36020083
C	2.04979657	-0.69769099	0.86480388
C	2.04963912	0.69829299	-0.86465999
C	1.67508010	-1.54928335	1.91041384
C	3.42118167	-0.45884120	0.56629207
C	1.67473162	1.54986670	-1.91021715
C	3.42107908	0.45975334	-0.56614717
C	2.68680434	-2.15432881	2.65127693
H	0.62193699	-1.73509730	2.12829534
C	4.40865825	-1.06988786	1.31329171
C	2.68631968	2.15517965	-2.65104775
H	0.62154587	1.73550367	-2.12804225
C	4.40841768	1.07106591	-1.31310955
C	4.04714445	-1.93346330	2.37458408
H	2.41654022	-2.82332956	3.47122655
H	5.46466520	-0.89509205	1.09309040
C	4.04670958	1.93456774	-2.37439827
H	2.41590477	2.82422707	-3.47090942
H	5.46446264	0.89655604	-1.09286114

N	1.24297508	0.00021716	0.00007474
C	5.11720542	2.59885315	-3.18176501
H	5.77472315	1.84857335	-3.64937164
H	4.69842583	3.23347872	-3.97363264
H	5.75853192	3.22353301	-2.53944396
C	5.11778131	-2.59813671	3.18144430
H	5.78024480	-1.84849275	3.64295006
H	4.69922246	-3.22738018	3.97771195
H	5.75402830	-3.22870054	2.53974923

Structure of Au1, T2 state

C	-1.56164863	-1.17396642	-0.04468223
C	-3.51211994	0.00009820	-0.00263812
C	-1.56163679	1.17405039	0.04206424
N	-2.88742565	1.19674512	0.04118967
N	-2.88744070	-1.19658755	-0.04553961
C	-0.68337495	-2.32423800	-0.08953621
C	-1.16577715	-3.65164440	-0.13761747
C	0.75478545	-2.04944458	-0.08297320
C	-0.26994641	-4.69821896	-0.18076829
H	-2.24393103	-3.82731550	-0.13947652
C	1.61541513	-3.13857996	-0.13132281
C	1.14637869	-4.46720039	-0.17921927
H	-0.63674294	-5.72742815	-0.21753033
H	2.69622887	-2.96769913	-0.12930751
C	2.08051379	-5.63008235	-0.22842654
H	1.90944196	-6.23435568	-1.13562079
H	3.13153861	-5.31122694	-0.21834418
H	1.91386882	-6.30518785	0.62819493
C	-0.68335924	2.32428306	0.08782436
C	-1.16574590	3.65170899	0.13533429
C	0.75480125	2.04942094	0.08270557
C	-0.26990999	4.69824762	0.17929446
H	-2.24389263	3.82743358	0.13612412
C	1.61543285	3.13852609	0.13181530
C	1.14641075	4.46716400	0.17914354
H	-0.63669481	5.72747620	0.21562261
H	2.69623957	2.96759053	0.13085904
C	2.08053746	5.63002007	0.22912976
H	1.91484324	6.30495284	-0.62781477
H	1.90850575	6.23448999	1.13600906
H	3.13156105	5.31112481	0.22023549
Au	1.11784042	-0.00001893	0.00007411
C	3.13610157	-0.00005018	0.00093397

C	3.84893905	-0.69370669	0.99035023
C	3.84974189	0.69360868	-0.98790320
C	5.23902652	-0.67491639	0.97301755
H	3.34483181	-1.25395217	1.78012462
C	5.23981351	0.67476808	-0.96948298
H	3.34626995	1.25388237	-1.77806255
C	5.96867305	-0.00009338	0.00204952
H	7.05887786	-0.00011434	0.00247560
F	5.90541394	-1.33398656	1.92518533
F	5.90697083	1.33382123	-1.92112292
C	-4.98231326	0.00013951	-0.00321657
C	-5.70078662	-1.20587855	-0.04766982
C	-5.70071544	1.20634802	0.03659634
C	-7.09025473	-1.20067577	-0.05041344
H	-5.15076936	-2.14759111	-0.08306440
C	-7.09019150	1.20142522	0.03349085
H	-5.15064873	2.14821173	0.06675689
C	-7.81413434	0.00036174	-0.00720542
H	-7.63020102	-2.15091883	-0.08918509
H	-7.63011000	2.15206596	0.06101435
C	-9.31331386	-0.00069763	0.02333091
H	-9.72759463	-0.87526167	-0.49866836
H	-9.67921503	-0.03864344	1.06344426
H	-9.72758145	0.90952798	-0.43365234
N	-0.89423865	0.00002252	-0.00087615

Structure of Au2, T2 state

C	-2.03595648	1.17310470	0.01358715
C	-3.98224200	-0.00820405	-0.00515680
C	-2.02766123	-1.17584894	-0.01470296
N	-3.35358130	-1.20348701	-0.01721551
N	-3.36202292	1.19142477	0.01011478
C	-1.16129441	2.32717777	0.03207849
C	-1.64874367	3.65357940	0.04617896
C	0.27820001	2.05773219	0.03596472
C	-0.75715665	4.70449716	0.06627606
H	-2.72755221	3.82512183	0.04081980
C	1.13415841	3.15144242	0.06035249
C	0.66001470	4.47908277	0.07447225
H	-1.12809001	5.73282306	0.07695972
H	2.21578034	2.98562277	0.06603074
C	1.58959747	5.64642763	0.09788418
H	1.41252514	6.27298191	0.98865766
H	2.64184237	5.33145082	0.10020796

H	1.42412287	6.29888842	-0.77634890
C	-1.14477996	-2.32372636	-0.02901365
C	-1.62265931	-3.65357802	-0.04492134
C	0.29276855	-2.04405493	-0.02634771
C	-0.72347976	-4.69811102	-0.05995287
H	-2.70023368	-3.83285852	-0.04460876
C	1.15665483	-3.13160248	-0.04546044
C	0.69207756	-4.46258257	-0.06119376
H	-1.08701176	-5.72907283	-0.07172608
H	2.23707075	-2.95795172	-0.04581586
C	1.63013129	-5.62320828	-0.08046497
H	1.46062300	-6.28098482	0.78893449
H	1.46643528	-6.24673938	-0.97596494
H	2.68006395	-5.30073341	-0.07115637
Au	0.64953800	0.00809257	0.00500797
C	2.66675574	0.01466760	0.00847719
C	3.38858415	0.68797467	-0.99014314
C	3.38979264	-0.65095661	1.01127401
C	4.78214115	0.69048128	-0.99403090
H	2.85881790	1.22607783	-1.78024047
C	4.78338187	-0.64245910	1.02078010
H	2.86106131	-1.18638450	1.80389454
C	5.48261823	0.02583383	0.01422316
C	-5.45257705	-0.01318868	-0.00835441
C	-6.17505926	1.19119157	0.00145903
C	-6.16692808	-1.22230076	-0.02549297
C	-7.56450594	1.18151582	-0.00419174
H	-5.62814863	2.13533962	0.01038886
C	-7.55642144	-1.22187695	-0.03102629
H	-5.61368124	-2.16269538	-0.03754177
C	-8.28436930	-0.02261918	-0.01777166
H	-8.10764602	2.13071496	-0.00024434
H	-8.09312424	-2.17458561	-0.04826535
C	-9.78360576	-0.02791615	0.00982458
H	-10.15153537	-0.03974534	1.04984636
H	-10.19989312	0.86866413	-0.47166223
H	-10.19395649	-0.91719943	-0.48992908
H	5.32859247	-1.15569134	1.81618854
H	5.32626700	1.22004280	-1.77945881
C	6.98060583	-0.01386538	-0.01380086
F	7.44062085	-1.06020128	-0.72058230
F	7.50553216	-0.12294692	1.21299401
F	7.50352943	1.08354434	-0.57520672
N	-1.36485657	0.00097358	0.00077568

Structure of Au3, T2 state

C	1.45979785	1.17360217	0.01429372
C	3.40964582	-0.00157274	0.00227621
C	1.45834440	-1.17429193	-0.01237698
N	2.78486944	-1.19880759	-0.01103067
N	2.78634184	1.19643386	0.01493814
C	0.58104354	2.32513994	0.02994024
C	1.06573208	3.65270464	0.04608426
C	-0.85832676	2.05212711	0.02982072
C	0.17218245	4.70201397	0.06527647
H	2.14423074	3.82646174	0.04335352
C	-1.71572007	3.14511312	0.05365542
C	-1.24447186	4.47362111	0.07047433
H	0.54114579	5.73107349	0.07778268
H	-2.79679883	2.97565318	0.05640253
C	-2.17653861	5.63919870	0.09463466
H	-2.00769997	6.29685664	-0.77501916
H	-3.22811551	5.32190764	0.08945053
H	-2.00621399	6.26129339	0.98990501
C	0.57817043	-2.32474462	-0.02810180
C	1.06113932	-3.65294712	-0.04340027
C	-0.86085573	-2.04991929	-0.02849817
C	0.16622588	-4.70111763	-0.06149723
H	2.13941245	-3.82811058	-0.04053570
C	-1.71967967	-3.14179533	-0.05069676
C	-1.25014368	-4.47092301	-0.06649296
H	0.53386701	-5.73066296	-0.07305707
H	-2.80053337	-2.97086015	-0.05337141
C	-2.18372012	-5.63531295	-0.08956175
H	-2.01416666	-6.25856072	-0.98416584
H	-2.01580552	-6.29227667	0.78080447
H	-3.23488134	-5.31663793	-0.08471882
Au	-1.22622952	0.00131460	-0.00018346
C	-3.24594365	0.00190129	-0.00069292
C	-3.97346784	-0.68706096	0.98118501
C	-3.97262236	0.69525938	-0.98009550
C	-5.36957762	-0.67712064	0.98456709
H	-3.44746740	-1.24174719	1.76342703
C	-5.36876771	0.69089525	-0.98074515
H	-3.44599196	1.25490304	-1.75835325
C	-6.09568146	0.00596368	0.00097355
C	4.88032832	-0.00244665	0.00244779
C	5.59959883	1.20375585	0.01796335
C	5.59817889	-1.20951797	-0.00883308

C	6.98912442	1.19790785	0.02049829
H	5.04998724	2.14627918	0.03115612
C	6.98772644	-1.20535232	-0.00624983
H	5.04747654	-2.15146103	-0.01648938
C	7.71240658	-0.00413932	0.00569157
H	7.52958088	2.14853347	0.03655389
H	7.52707263	-2.15672553	-0.01136703
C	9.21163431	-0.00460396	-0.02522194
H	9.57747822	0.00883840	-1.06596243
H	9.62651712	0.88166582	0.47618136
H	9.62553667	-0.90409345	0.45288607
H	-5.90532462	1.23924595	-1.76160292
H	-5.90674419	-1.21578608	1.77171729
C	-7.59776404	-0.01889736	-0.01696003
N	0.79271408	0.00007543	0.00004633
H	-8.01161803	0.89487644	-0.46828236
H	-7.97192181	-0.87151474	-0.60874435
H	-8.01235804	-0.12155874	0.99671580

Structure of Au4, T2 state

C	2.61219814	1.17297246	0.04866081
C	2.61195750	-1.17400451	-0.04084325
N	3.94385534	-1.19635337	-0.03725710
N	3.94413019	1.19476516	0.05189019
C	1.73547563	2.32229058	0.09534509
C	2.22173236	3.65020569	0.14721518
C	0.29572226	2.05089361	0.08646155
C	1.32892949	4.69873672	0.19126245
H	3.30062966	3.82125308	0.15103998
C	-0.56106508	3.14119334	0.13585832
C	-0.08867255	4.47126949	0.18708743
H	1.69837538	5.72694697	0.23078330
H	-1.64250380	2.97368328	0.13243168
C	-1.02091510	5.63563739	0.23696351
H	-2.07248544	5.31833887	0.22602769
H	-0.85017320	6.23922009	1.14478789
H	-0.85366916	6.31159904	-0.61900965
C	1.73491872	-2.32306322	-0.08779644
C	2.22075943	-3.65135755	-0.13498347
C	0.29519941	-2.05103145	-0.08454949
C	1.32765519	-4.69952410	-0.18097280
H	3.29958219	-3.82291438	-0.13394949
C	-0.56187239	-3.14099123	-0.13587249
C	-0.08989156	-4.47139789	-0.18320182

H	1.69676390	-5.72798093	-0.21711679
H	-1.64324203	-2.97299462	-0.13656073
C	-1.02247982	-5.63537460	-0.23551567
H	-0.85689241	-6.31221940	0.62004710
H	-2.07394424	-5.31766607	-0.22617363
H	-0.85052224	-6.23813956	-1.14368901
Au	-0.06780087	-0.00001404	0.00029338
C	-2.08498445	0.00033782	-0.00149662
C	-2.79896728	-0.69985991	0.98239828
C	-2.79691163	0.70079734	-0.98669592
C	-4.18902094	-0.68044580	0.96342583
H	-2.29583281	-1.26567046	1.76877028
C	-4.18700316	0.68195832	-0.97022891
H	-2.29214174	1.26638683	-1.77218294
C	-4.91722993	0.00091159	-0.00405344
H	-6.00743354	0.00114148	-0.00502874
F	-4.85677815	-1.34559827	1.91035890
F	-4.85277870	1.34738537	-1.91836444
C	6.05001780	-0.00002075	-0.02371377
H	6.44805083	0.88810656	0.48289213
H	6.39149501	0.03057737	-1.07225970
H	6.44781492	-0.91688556	0.42921218
C	4.55848886	-0.00083894	0.00752082
N	1.94832358	-0.00034375	0.00182408

Structure of Au5, T2 state

Au	-0.70082036	-0.00000619	0.00016264
C	-0.36155025	-2.05148579	-0.28313104
C	1.04270002	-2.30931603	-0.30968607
C	1.49605835	-3.62839578	-0.47704131
C	0.57584508	-4.66669389	-0.61459787
C	-0.80423423	-4.43133229	-0.59433024
C	-1.25209342	-3.09796193	-0.42960696
H	2.57087554	-3.82522647	-0.49190278
H	0.93962868	-5.69063859	-0.74160906
H	-2.33063020	-2.90825304	-0.41752316
C	-1.79902543	-5.54397146	-0.74678519
H	-1.30445452	-6.52209410	-0.83073140
H	-2.48854642	-5.58381781	0.11255650
H	-2.42319970	-5.40263368	-1.64486333
C	1.91438399	-1.17088674	-0.15220379
C	1.91453862	1.17008153	0.15554686
C	1.04300507	2.30878578	0.31205519
C	1.49660813	3.62769501	0.48000542

C	-0.36128557	2.05138701	0.28390274
C	0.57657879	4.66628797	0.61658269
H	2.57147290	3.82416887	0.49608292
C	-1.25165044	3.09814514	0.42944612
C	-0.80354558	4.43136207	0.59475059
H	0.94054820	5.69010681	0.74406905
H	-2.33023526	2.90879545	0.41616036
C	-1.79815206	5.54430627	0.74616529
H	-2.42334305	5.40312290	1.64355971
H	-1.30336886	6.52227153	0.83068895
H	-2.48672533	5.58439430	-0.11392338
C	-3.50947056	0.64251758	-0.91186965
C	-3.51049310	-0.64185761	0.90951398
C	-3.12354422	1.42500061	-2.00822072
C	-4.87933945	0.42146893	-0.59637390
C	-3.12581226	-1.42459289	2.00612310
C	-4.88000742	-0.42031124	0.59282172
C	-4.13271491	1.98347206	-2.79101710
H	-2.06904684	1.59512201	-2.23131855
C	-5.86793465	0.98492129	-1.38476618
C	-4.13587566	-1.98276579	2.78798513
H	-2.07157460	-1.59512555	2.23013881
C	-5.86949789	-0.98347145	1.38029688
C	-5.48142790	1.76887379	-2.48636780
H	-3.86537441	2.59880983	-3.65196973
H	-6.92515404	0.82982643	-1.16098439
C	-5.48424175	-1.76765183	2.48217552
H	-3.86951228	-2.59827684	3.64911667
H	-6.92646306	-0.82799810	1.15557972
H	-6.25118860	2.22019033	-3.11550840
H	-6.25471609	-2.21875019	3.11059810
N	-2.70401613	0.00023092	-0.00079704
C	5.35260203	-0.00081652	0.00351739
C	6.06858737	-1.19606910	-0.14846071
C	6.06874660	1.19394432	0.15979042
C	7.45951851	-1.19330486	-0.14548196
H	5.51184299	-2.12714900	-0.26574502
C	7.45954946	1.19040224	0.16188775
H	5.51203085	2.12434347	0.28257169
C	8.18253635	-0.00134263	0.00658787
H	8.00006546	-2.13706608	-0.26154656
H	8.00019559	2.13268696	0.28914481
C	9.68247084	0.00317772	-0.02429878
H	10.04849007	0.15329413	-1.05408731

H	10.09684819	0.81650070	0.58888202
H	10.09733969	-0.95058106	0.33237133
C	3.86816897	-0.00070690	0.00304155
N	3.27380966	-1.18967487	-0.14984784
N	3.27395698	1.18845784	0.15508620
N	1.26753445	-0.00028560	0.00121619

Structure of Au6, T2 state

C	-0.97111502	1.95963532	-0.26419571
C	-2.43474886	2.12170515	-0.30856878
C	-2.98152858	3.43667066	-0.49871702
C	-2.14273929	4.51273436	-0.62099560
C	-0.71561214	4.37330552	-0.57219991
C	-0.17419225	3.07992198	-0.39037770
H	-4.06345084	3.57996045	-0.53753905
H	-2.56681463	5.51112120	-0.75926956
H	0.91379447	2.96816205	-0.35508449
C	0.15309082	5.57646717	-0.72721885
H	-0.10093140	6.34604414	0.02114817
H	1.21762242	5.32735462	-0.62056500
H	0.00599916	6.04550694	-1.71542716
C	-3.19886628	0.95158450	-0.15540082
C	-4.61582396	0.76346435	-0.14352307
N	-5.18797756	-0.40441229	0.00274436
H	-5.26835861	1.63562823	-0.26148817
C	-2.97195406	-1.41924160	0.15639682
C	-4.38386088	-1.50454752	0.15337931
H	-4.87985760	-2.46921025	0.27586249
C	-1.99578443	-2.49386585	0.31133156
C	-2.33194121	-3.84268484	0.48795771
C	-0.62643751	-2.09100181	0.27953281
C	-1.32534552	-4.79000314	0.63738961
H	-3.37725068	-4.16011819	0.50907775
C	0.35398523	-3.06101852	0.44158036
C	0.02806280	-4.41776323	0.62009933
H	-1.59160668	-5.84168333	0.77022361
H	1.40830972	-2.76878861	0.41654157
Au	-0.47576085	-0.03383518	-0.00280076
N	-2.43489070	-0.21078621	0.00531618
C	1.11234365	-5.43642577	0.80303588
H	1.53084594	-5.37488569	1.82160705
H	0.74102379	-6.46010892	0.65612185
H	1.94464585	-5.26020573	0.10507548
C	2.37535288	-0.43923508	-0.93242488

C	2.29198012	0.81704186	0.90061903
C	2.06818777	-1.22705520	-2.05215109
C	3.72865914	-0.12605374	-0.61172681
C	1.88504336	1.55188526	2.02456857
C	3.67450449	0.69082149	0.57566434
C	3.11807877	-1.69826006	-2.83232647
H	1.03029211	-1.46657088	-2.29539502
C	4.76759712	-0.61057344	-1.41614772
C	2.86390365	2.15849351	2.80346655
H	0.82521402	1.64665357	2.27218637
C	4.64075256	1.31022299	1.37811749
C	4.45797480	-1.39613048	-2.52216377
H	2.89674174	-2.31626043	-3.70658207
H	5.80791944	-0.37413984	-1.17637597
C	4.23131054	2.04313361	2.48777968
H	2.56358426	2.73716914	3.68107361
H	5.70269335	1.21756364	1.13448129
H	5.25866541	-1.78177717	-3.15787717
H	4.97435557	2.53231302	3.12224381
N	1.52664029	0.13518238	-0.01598402

Structure of Au7, T2 state

C	-1.07980005	2.00961167	0.47199256
C	-2.47680712	2.29290373	0.54558578
C	-2.92000296	3.58962898	0.84202049
C	-1.99344995	4.60135873	1.06363756
C	-0.61361107	4.34736059	0.99827814
C	-0.18006120	3.04374050	0.69754974
H	-3.98761459	3.81686349	0.89342519
H	-2.34377225	5.61191284	1.28885191
H	0.89422401	2.84392639	0.63710926
C	0.38250398	5.43387117	1.27005078
H	1.27835674	5.32140910	0.64191086
H	-0.04588736	6.43164247	1.09954733
H	0.71765681	5.39401915	2.32038808
C	-3.36371660	1.16294815	0.28530138
C	-4.77601496	1.13163842	0.28429853
N	-5.48702157	-0.00798998	0.02132514
H	-5.34846964	2.03644687	0.49766484
C	-3.39569264	-1.17426000	-0.26506064
C	-4.81899622	-1.10586018	-0.24105302
H	-5.39874777	-2.01082315	-0.45393239
C	-2.53601024	-2.26052652	-0.52244403
C	-2.96852360	-3.59430006	-0.81997364

C	-1.09090247	-1.97363453	-0.48829485
C	-2.04203921	-4.56462975	-1.10013542
H	-4.03355496	-3.83547795	-0.83634971
C	-0.20148652	-2.98803781	-0.79549483
C	-0.63190213	-4.29530533	-1.09976947
H	-2.38094786	-5.57767206	-1.33421738
H	0.87243832	-2.77808971	-0.77652705
Au	-0.76044632	0.00586227	0.00535943
N	-2.72902004	0.02254003	0.01382877
C	0.33193840	-5.39580496	-1.39573740
H	1.37180508	-5.04215324	-1.37825792
H	0.23373527	-6.21568816	-0.66368991
H	0.13262701	-5.83956260	-2.38599847
C	2.05202209	-0.71824404	0.86932086
C	2.06527275	0.68757992	-0.85022533
C	1.69467713	-1.57480064	1.92135372
C	3.42352170	-0.48051075	0.57125525
C	1.72407388	1.54749181	-1.90497170
C	3.43213435	0.43507702	-0.54329005
C	2.70857267	-2.18180397	2.64993587
H	0.64333851	-1.76146492	2.15324594
C	4.42341255	-1.10704465	1.32687270
C	2.74917729	2.14368176	-2.62684139
H	0.67644873	1.74545494	-2.14445408
C	4.44360400	1.05050316	-1.29259239
C	4.07888692	-1.96418024	2.37195013
H	2.43984828	-2.85382671	3.47065902
H	5.47760147	-0.92496978	1.09656495
C	4.11520150	1.91141365	-2.33973490
H	2.49301852	2.81826387	-3.44946507
H	5.49424891	0.85668796	-1.05578046
N	1.24860572	-0.01071802	0.00697204
C	5.17847594	2.58768677	-3.15987356
H	5.09722169	2.31858289	-4.22579966
H	5.09371466	3.68531456	-3.10290217
H	6.18677905	2.31105723	-2.82000697
C	5.12940175	-2.65180491	3.19906278
H	5.04446611	-2.38121515	4.26432980
H	5.03276585	-3.74845526	3.14212986
H	6.14284988	-2.38661261	2.86543570

2.6 Frequency Calculation

Structure of Au1, S0 state

	1	2	3
	A	A	A
Frequencies --	15.9359	26.1753	26.3595
Red. masses --	7.7751	1.0232	1.0240
Frc consts --	0.0012	0.0004	0.0004
IR Inten --	0.0007	0.7180	0.8280

Structure of Au2, S0 state

	1	2	3
	A	A	A
Frequencies --	12.7491	15.4740	21.6305
Red. masses --	8.3175	17.1726	7.0045
Frc consts --	0.0008	0.0024	0.0019
IR Inten --	0.0060	0.0515	0.0390

Structure of Au3, S0 state

	1	2	3
	A	A	A
Frequencies --	16.0719	24.6959	24.9724
Red. masses --	5.2949	1.0170	1.0162
Frc consts --	0.0008	0.0004	0.0004
IR Inten --	0.1366	0.7350	0.7864

Structure of Au4, S0 state

	1	2	3
	A	A	
Frequencies --	24.4733	24.8529	26.3097
Red. masses --	1.0200	1.0170	5.7701
Frc consts --	0.0004	0.0004	0.0024
IR Inten --	0.8354	0.8316	0.1601

Structure of Au5, S0 state

	1	2	3
	A	A	A
Frequencies --	15.9812	22.8708	28.1162
Red. masses --	6.0734	5.8062	1.7961
Frc consts --	0.0009	0.0018	0.0008
IR Inten --	0.0113	0.0223	0.425

Structure of Au6, S0 state

	1	2	3
	A	A	A
Frequencies --	23.7777	26.4523	27.0520
Red. masses --	5.2303	5.3526	1.5263
Frc consts --	0.0017	0.0022	0.0007
IR Inten --	0.0091	0.0032	1.5711

Structure of Au7, S0 state

	1	2	3
	A	A	A
Frequencies --	19.1556	21.1972	21.2512
Red. masses --	4.2278	1.0482	1.1046
Frc consts --	0.0009	0.0003	0.0003
IR Inten --	0.0134	0.3725	1.5423

Structure of Au1, S1 state

	1	2	3
	A	A	A
Frequencies --	9.7310	16.5574	24.4991
Red. masses --	10.0721	7.2564	3.0310
Frc consts --	0.0006	0.0012	0.0011
IR Inten --	0.0120	0.0637	0.0047

Structure of Au2, S1 state

	1	2	3
	A	A	A
Frequencies --	7.9095	13.4738	14.3423
Red. masses --	17.3823	7.5580	7.8722
Frc consts --	0.0006	0.0008	0.0010
IR Inten --	0.0615	0.2137	0.1903

Structure of Au3, S1 state

	1	2	3
	A	A	
Frequencies --	5.3604	15.8914	20.6739
Red. masses --	5.2940	5.1571	3.3034
Frc consts --	0.0001	0.0008	0.0008
IR Inten --	0.5629	0.6540	0.8254

Structure of Au4, S1 state

	1	2	3
	A	A	A
Frequencies --	7.5812	15.0766	24.5853
Red. masses --	9.9699	3.6980	7.8540
Frc consts --	0.0003	0.0005	0.0028
IR Inten --	0.0465	1.3488	0.298

Structure of Au5, S1 state

	1	2	3
	A	A	A
Frequencies --	11.4127	16.7759	22.3301
Red. masses --	7.0253	5.4991	5.8589
Frc consts --	0.0005	0.0009	0.0017
IR Inten --	0.7309	0.0475	0.8424

Structure of Au6, S1 state

	1	2	3
	A	A	
Frequencies --	16.0715	23.9627	28.3918
Red. masses --	5.5053	5.6697	5.3139
Frc consts --	0.0008	0.0019	0.0025
IR Inten --	0.0632	0.5958	0.4928

Structure of Au7, S1 state

	1	2	3
	A	A	A
Frequencies --	14.3854	20.4892	25.4586
Red. masses --	4.9355	4.9627	4.8533
Frc consts --	0.0006	0.0012	0.0019
IR Inten --	0.0747	0.5794	0.7165

Structure of Au1, T1 state

	1	2	3
	A	A	
Frequencies --	15.2008	27.8449	28.8147
Red. masses --	7.3444	5.2662	1.1656
Frc consts --	0.0010	0.0024	0.0006

IR Inten --	0.1167	0.0763	0.8389

Structure of Au2, T1 state

	1	2	3
	A	A	A
Frequencies --	12.2997	15.0366	21.6481
Red. masses --	7.9731	17.3024	9.7028
Frc consts --	0.0007	0.0023	0.0027
IR Inten --	0.0949	0.0498	0.0004

Structure of Au3, T1 state

	1	2	3
	A	A	A
Frequencies --	11.1924	15.5570	27.5532
Red. masses --	1.0245	5.1033	1.0639
Frc consts --	0.0001	0.0007	0.0005
IR Inten --	0.7852	0.5434	0.639

Structure of Au4, T1 state

	1	2	3
	A	A	
Frequencies --	21.2957	25.5918	28.2338
Red. masses --	1.0710	4.6080	9.2096
Frc consts --	0.0003	0.0018	0.0043
IR Inten --	1.0331	0.2202	0.0486

Structure of Au5, T1 state

	1	2	3
	A	A	A
Frequencies --	13.8036	23.3171	24.8883
Red. masses --	6.7692	5.2947	5.5064
Frc consts --	0.0008	0.0017	0.0020
IR Inten --	1.1608	0.8319	0.109

Structure of Au6, T1 state

	1	2	3
	A	A	A
Frequencies --	17.0493	23.0869	26.9804
Red. masses --	5.6134	5.5089	5.7418

Frc consts --	0.0010	0.0017	0.0025
IR Inten --	0.1262	0.0102	0.4804

Structure of Au7, T1 state

	1	2	3
	A	A	A
Frequencies --	20.2249	20.4910	24.8580
Red. masses --	4.5794	4.7561	5.3877
Frc consts --	0.0011	0.0012	0.0020
IR Inten --	0.6271	0.0031	0.3756

Structure of Au1, T2 state

	1	2	3
	A	A	A
Frequencies --	16.1918	27.1236	28.3009
Red. masses --	7.7538	4.7271	1.4893
Frc consts --	0.0012	0.0020	0.0007
IR Inten --	0.0144	0.1006	0.8807

Structure of Au2, T2 state

	1	2	3
	A	A	A
Frequencies --	12.8627	14.4097	21.3254
Red. masses --	8.4019	17.1157	8.8993
Frc consts --	0.0008	0.0021	0.0024
IR Inten --	0.0362	0.0523	0.0434

Structure of Au3, T2 state

	1	2	3
	A	A	A
Frequencies --	16.4424	26.0208	26.4219
Red. masses --	5.2779	1.2130	2.3631
Frc consts --	0.0008	0.0005	0.0010
IR Inten --	0.0792	1.2368	0.6731

Structure of Au4, T2 state

	1	2	3
	A	A	A
Frequencies --	26.2619	27.4857	27.9521

Red. masses --	5.2816	2.3411	1.5556
Frc consts --	0.0021	0.0010	0.0007
IR Inten --	0.5910	0.3193	0.5429

Structure of Au5, T2 state

	1	2	3
	A	A	A
Frequencies --	16.6889	24.1103	26.6448
Red. masses --	6.0253	5.9418	3.6173
Frc consts --	0.0010	0.0020	0.0015
IR Inten --	1.0574	1.3466	0.1493

Structure of Au6, T2 state

	1	2	3
	A	A	A
Frequencies --	21.5361	23.6498	25.3835
Red. masses --	1.0537	5.7953	4.8297
Frc consts --	0.0003	0.0019	0.0018
IR Inten --	0.6778	0.1449	0.0494

Structure of Au7, T2 state

	1	2	3
	A	A	A
Frequencies --	19.1358	20.0666	26.8970
Red. masses --	5.0007	4.7399	5.1972
Frc consts --	0.0011	0.0011	0.0022
IR Inten --	0.0656	0.4403	1.5238

2.7 Full CDA Results

Charge decomposition analysis is widespread adopted for structural analysis. It was firstly proposed by Dapprich et al,[25] expanded by Gorelsky et al (ECDA)[26a] and generalized by Tian Lu et al (GCDA).[22] In this work, GCDA are used for studying electronic structure of Au6.

Fragment orbitals that contribute over 1% to complex orbitals have been listed below for complex orbitals range from MO 50 to 180 and such range has already covered all orbitals that may be of importance to the coordinating structure of Au6.

Occupation number of orbital 50 of the complex: 2.00000000

Orbital	24 of fragment	3, Occ: 2.00000	Contribution:	1.50 %
Orbital	26 of fragment	3, Occ: 2.00000	Contribution:	2.16 %
Orbital	29 of fragment	3, Occ: 2.00000	Contribution:	89.06 %
Orbital	33 of fragment	3, Occ: 2.00000	Contribution:	1.13 %
Orbital	37 of fragment	3, Occ: 2.00000	Contribution:	1.31 %
Orbital	17 of fragment	1, Occ: 2.00000	Contribution:	1.06 %

Sum of values shown above: 96.22 %

Occupation number of orbital 51 of the complex: 2.00000000

Orbital	28 of fragment	3, Occ: 2.00000	Contribution:	7.79 %
Orbital	18 of fragment	1, Occ: 2.00000	Contribution:	88.00 %
Orbital	19 of fragment	1, Occ: 2.00000	Contribution:	2.13 %

Sum of values shown above: 97.92 %

Occupation number of orbital 52 of the complex: 2.00000000

Orbital	25 of fragment	3, Occ: 2.00000	Contribution:	2.59 %
Orbital	31 of fragment	3, Occ: 2.00000	Contribution:	82.82 %
Orbital	32 of fragment	3, Occ: 2.00000	Contribution:	9.95 %
Orbital	39 of fragment	3, Occ: 2.00000	Contribution:	1.18 %
Orbital	19 of fragment	1, Occ: 2.00000	Contribution:	1.44 %

Sum of values shown above: 97.97 %

Occupation number of orbital 53 of the complex: 2.00000000

Orbital	30 of fragment	3, Occ: 2.00000	Contribution:	11.05 %
Orbital	33 of fragment	3, Occ: 2.00000	Contribution:	3.50 %
Orbital	16 of fragment	1, Occ: 2.00000	Contribution:	1.36 %
Orbital	17 of fragment	1, Occ: 2.00000	Contribution:	2.53 %
Orbital	20 of fragment	1, Occ: 2.00000	Contribution:	75.04 %

Sum of values shown above: 93.49 %

Occupation number of orbital 54 of the complex: 2.00000000
Orbital 31 of fragment 3, Occ: 2.00000 Contribution: 1.45 %
Orbital 18 of fragment 1, Occ: 2.00000 Contribution: 2.48 %
Orbital 19 of fragment 1, Occ: 2.00000 Contribution: 95.08 %
Sum of values shown above: 99.01 %

Occupation number of orbital 55 of the complex: 2.00000000
Orbital 26 of fragment 3, Occ: 2.00000 Contribution: 3.57 %
Orbital 30 of fragment 3, Occ: 2.00000 Contribution: 71.30 %
Orbital 33 of fragment 3, Occ: 2.00000 Contribution: 6.98 %
Orbital 20 of fragment 1, Occ: 2.00000 Contribution: 15.94 %
Sum of values shown above: 97.80 %

Occupation number of orbital 56 of the complex: 2.00000000
Orbital 10 of fragment 2, Occ: 0.00000 Contribution: 1.10 %
Orbital 29 of fragment 3, Occ: 2.00000 Contribution: 1.38 %
Orbital 30 of fragment 3, Occ: 2.00000 Contribution: 10.08 %
Orbital 33 of fragment 3, Occ: 2.00000 Contribution: 73.74 %
Orbital 34 of fragment 3, Occ: 2.00000 Contribution: 4.33 %
Orbital 38 of fragment 3, Occ: 2.00000 Contribution: 1.82 %
Orbital 20 of fragment 1, Occ: 2.00000 Contribution: 1.41 %
Sum of values shown above: 93.86 %

Occupation number of orbital 57 of the complex: 2.00000000
Orbital 27 of fragment 3, Occ: 2.00000 Contribution: 2.33 %
Orbital 31 of fragment 3, Occ: 2.00000 Contribution: 10.47 %
Orbital 32 of fragment 3, Occ: 2.00000 Contribution: 82.47 %
Orbital 36 of fragment 3, Occ: 2.00000 Contribution: 2.16 %
Orbital 39 of fragment 3, Occ: 2.00000 Contribution: 1.15 %
Sum of values shown above: 98.59 %

Occupation number of orbital 58 of the complex: 2.00000000
Orbital 20 of fragment 1, Occ: 2.00000 Contribution: 1.39 %
Orbital 21 of fragment 1, Occ: 2.00000 Contribution: 93.13 %
Sum of values shown above: 94.52 %

Occupation number of orbital 59 of the complex: 2.00000000
Orbital 30 of fragment 3, Occ: 2.00000 Contribution: 1.54 %
Orbital 33 of fragment 3, Occ: 2.00000 Contribution: 5.02 %

Orbital 34 of fragment 3, Occ: 2.00000 Contribution: 88.43 %
Sum of values shown above: 94.98 %

Occupation number of orbital 60 of the complex: 2.00000000
Orbital 5 of fragment 2, Occ: 2.00000 Contribution: 1.32 %
Orbital 28 of fragment 3, Occ: 2.00000 Contribution: 1.67 %
Orbital 35 of fragment 3, Occ: 2.00000 Contribution: 91.35 %
Orbital 36 of fragment 3, Occ: 2.00000 Contribution: 1.36 %
Sum of values shown above: 95.70 %

Occupation number of orbital 61 of the complex: 2.00000000
Orbital 22 of fragment 1, Occ: 2.00000 Contribution: 91.43 %
Orbital 24 of fragment 1, Occ: 2.00000 Contribution: 1.63 %
Orbital 26 of fragment 1, Occ: 2.00000 Contribution: 2.16 %
Sum of values shown above: 95.22 %

Occupation number of orbital 62 of the complex: 2.00000000
Orbital 9 of fragment 2, Occ: 0.00000 Contribution: 9.17 %
Orbital 29 of fragment 3, Occ: 2.00000 Contribution: 2.66 %
Orbital 33 of fragment 3, Occ: 2.00000 Contribution: 2.07 %
Orbital 37 of fragment 3, Occ: 2.00000 Contribution: 68.02 %
Orbital 38 of fragment 3, Occ: 2.00000 Contribution: 3.22 %
Orbital 45 of fragment 3, Occ: 2.00000 Contribution: 1.41 %
Orbital 62 of fragment 3, Occ: 2.00000 Contribution: 1.10 %
Orbital 68 of fragment 3, Occ: 2.00000 Contribution: 2.22 %
Orbital 23 of fragment 1, Occ: 2.00000 Contribution: 3.11 %
Sum of values shown above: 92.99 %

Occupation number of orbital 63 of the complex: 2.00000000
Orbital 8 of fragment 2, Occ: 2.00000 Contribution: 1.48 %
Orbital 10 of fragment 2, Occ: 0.00000 Contribution: 1.64 %
Orbital 34 of fragment 3, Occ: 2.00000 Contribution: 1.13 %
Orbital 37 of fragment 3, Occ: 2.00000 Contribution: 8.70 %
Orbital 38 of fragment 3, Occ: 2.00000 Contribution: 3.09 %
Orbital 20 of fragment 1, Occ: 2.00000 Contribution: 1.51 %
Orbital 21 of fragment 1, Occ: 2.00000 Contribution: 3.00 %
Orbital 23 of fragment 1, Occ: 2.00000 Contribution: 70.37 %
Orbital 27 of fragment 1, Occ: 2.00000 Contribution: 1.15 %
Orbital 42 of fragment 1, Occ: 2.00000 Contribution: 1.99 %
Sum of values shown above: 94.05 %

Occupation number of orbital 64 of the complex: 2.00000000
Orbital 32 of fragment 3, Occ: 2.00000 Contribution: 2.74 %
Orbital 35 of fragment 3, Occ: 2.00000 Contribution: 1.77 %
Orbital 36 of fragment 3, Occ: 2.00000 Contribution: 88.07 %
Orbital 39 of fragment 3, Occ: 2.00000 Contribution: 1.43 %
Orbital 43 of fragment 3, Occ: 2.00000 Contribution: 1.05 %
Orbital 24 of fragment 1, Occ: 2.00000 Contribution: 1.83 %
Sum of values shown above: 96.89 %

Occupation number of orbital 65 of the complex: 2.00000000
Orbital 36 of fragment 3, Occ: 2.00000 Contribution: 1.66 %
Orbital 22 of fragment 1, Occ: 2.00000 Contribution: 1.80 %
Orbital 24 of fragment 1, Occ: 2.00000 Contribution: 94.90 %
Sum of values shown above: 98.36 %

Occupation number of orbital 66 of the complex: 2.00000000
Orbital 8 of fragment 2, Occ: 2.00000 Contribution: 1.97 %
Orbital 10 of fragment 2, Occ: 0.00000 Contribution: 1.46 %
Orbital 33 of fragment 3, Occ: 2.00000 Contribution: 2.72 %
Orbital 34 of fragment 3, Occ: 2.00000 Contribution: 2.54 %
Orbital 37 of fragment 3, Occ: 2.00000 Contribution: 2.75 %
Orbital 38 of fragment 3, Occ: 2.00000 Contribution: 64.09 %
Orbital 62 of fragment 3, Occ: 2.00000 Contribution: 2.09 %
Orbital 65 of fragment 3, Occ: 2.00000 Contribution: 2.74 %
Orbital 23 of fragment 1, Occ: 2.00000 Contribution: 15.34 %
Sum of values shown above: 95.70 %

Occupation number of orbital 67 of the complex: 2.00000000
Orbital 5 of fragment 2, Occ: 2.00000 Contribution: 1.13 %
Orbital 32 of fragment 3, Occ: 2.00000 Contribution: 1.90 %
Orbital 36 of fragment 3, Occ: 2.00000 Contribution: 2.51 %
Orbital 39 of fragment 3, Occ: 2.00000 Contribution: 90.52 %
Sum of values shown above: 96.07 %

Occupation number of orbital 68 of the complex: 2.00000000
Orbital 9 of fragment 2, Occ: 0.00000 Contribution: 8.22 %
Orbital 37 of fragment 3, Occ: 2.00000 Contribution: 4.18 %
Orbital 38 of fragment 3, Occ: 2.00000 Contribution: 7.47 %

Orbital 40 of fragment 3, Occ: 2.00000 Contribution: 55.40 %
Orbital 65 of fragment 3, Occ: 2.00000 Contribution: 1.35 %
Orbital 68 of fragment 3, Occ: 2.00000 Contribution: 2.02 %
Orbital 23 of fragment 1, Occ: 2.00000 Contribution: 3.34 %
Orbital 25 of fragment 1, Occ: 2.00000 Contribution: 8.93 %
Orbital 27 of fragment 1, Occ: 2.00000 Contribution: 2.14 %
Orbital 42 of fragment 1, Occ: 2.00000 Contribution: 2.04 %
Sum of values shown above: 95.10 %

Occupation number of orbital 69 of the complex: 2.00000000
Orbital 38 of fragment 3, Occ: 2.00000 Contribution: 2.79 %
Orbital 40 of fragment 3, Occ: 2.00000 Contribution: 28.51 %
Orbital 23 of fragment 1, Occ: 2.00000 Contribution: 2.75 %
Orbital 25 of fragment 1, Occ: 2.00000 Contribution: 52.65 %
Orbital 27 of fragment 1, Occ: 2.00000 Contribution: 5.80 %
Orbital 42 of fragment 1, Occ: 2.00000 Contribution: 2.47 %
Sum of values shown above: 94.97 %

Occupation number of orbital 70 of the complex: 2.00000000
Orbital 41 of fragment 3, Occ: 2.00000 Contribution: 65.46 %
Orbital 22 of fragment 1, Occ: 2.00000 Contribution: 1.23 %
Orbital 26 of fragment 1, Occ: 2.00000 Contribution: 26.29 %
Sum of values shown above: 92.98 %

Occupation number of orbital 71 of the complex: 2.00000000
Orbital 7 of fragment 2, Occ: 2.00000 Contribution: 1.10 %
Orbital 41 of fragment 3, Occ: 2.00000 Contribution: 28.57 %
Orbital 22 of fragment 1, Occ: 2.00000 Contribution: 2.41 %
Orbital 26 of fragment 1, Occ: 2.00000 Contribution: 59.52 %
Orbital 28 of fragment 1, Occ: 2.00000 Contribution: 1.70 %
Orbital 30 of fragment 1, Occ: 2.00000 Contribution: 1.06 %
Sum of values shown above: 94.35 %

Occupation number of orbital 72 of the complex: 2.00000000
Orbital 9 of fragment 2, Occ: 0.00000 Contribution: 6.31 %
Orbital 38 of fragment 3, Occ: 2.00000 Contribution: 5.67 %
Orbital 40 of fragment 3, Occ: 2.00000 Contribution: 6.13 %
Orbital 62 of fragment 3, Occ: 2.00000 Contribution: 1.34 %
Orbital 65 of fragment 3, Occ: 2.00000 Contribution: 1.38 %
Orbital 23 of fragment 1, Occ: 2.00000 Contribution: 1.89 %

Orbital 25 of fragment 1, Occ: 2.00000 Contribution: 34.81 %
Orbital 27 of fragment 1, Occ: 2.00000 Contribution: 29.73 %
Orbital 29 of fragment 1, Occ: 2.00000 Contribution: 1.15 %
Orbital 37 of fragment 1, Occ: 2.00000 Contribution: 1.47 %
Orbital 42 of fragment 1, Occ: 2.00000 Contribution: 4.33 %
Sum of values shown above: 94.22 %

Occupation number of orbital 73 of the complex: 2.00000000
Orbital 7 of fragment 2, Occ: 2.00000 Contribution: 7.51 %
Orbital 44 of fragment 3, Occ: 2.00000 Contribution: 75.96 %
Orbital 48 of fragment 3, Occ: 2.00000 Contribution: 2.79 %
Orbital 57 of fragment 3, Occ: 2.00000 Contribution: 3.07 %
Orbital 59 of fragment 3, Occ: 2.00000 Contribution: 5.14 %
Orbital 26 of fragment 1, Occ: 2.00000 Contribution: 2.03 %
Sum of values shown above: 96.51 %

Occupation number of orbital 74 of the complex: 2.00000000
Orbital 8 of fragment 2, Occ: 2.00000 Contribution: 2.41 %
Orbital 9 of fragment 2, Occ: 0.00000 Contribution: 15.53 %
Orbital 10 of fragment 2, Occ: 0.00000 Contribution: 2.73 %
Orbital 37 of fragment 3, Occ: 2.00000 Contribution: 10.14 %
Orbital 40 of fragment 3, Occ: 2.00000 Contribution: 4.68 %
Orbital 42 of fragment 3, Occ: 2.00000 Contribution: 5.74 %
Orbital 45 of fragment 3, Occ: 2.00000 Contribution: 19.59 %
Orbital 47 of fragment 3, Occ: 2.00000 Contribution: 1.63 %
Orbital 54 of fragment 3, Occ: 2.00000 Contribution: 1.66 %
Orbital 58 of fragment 3, Occ: 2.00000 Contribution: 1.30 %
Orbital 62 of fragment 3, Occ: 2.00000 Contribution: 1.01 %
Orbital 68 of fragment 3, Occ: 2.00000 Contribution: 8.40 %
Orbital 27 of fragment 1, Occ: 2.00000 Contribution: 21.88 %
Sum of values shown above: 96.71 %

Occupation number of orbital 75 of the complex: 2.00000000
Orbital 5 of fragment 2, Occ: 2.00000 Contribution: 10.90 %
Orbital 35 of fragment 3, Occ: 2.00000 Contribution: 2.27 %
Orbital 43 of fragment 3, Occ: 2.00000 Contribution: 48.03 %
Orbital 46 of fragment 3, Occ: 2.00000 Contribution: 15.10 %
Orbital 48 of fragment 3, Occ: 2.00000 Contribution: 1.44 %
Orbital 49 of fragment 3, Occ: 2.00000 Contribution: 2.85 %
Orbital 53 of fragment 3, Occ: 2.00000 Contribution: 6.15 %
Orbital 60 of fragment 3, Occ: 2.00000 Contribution: 2.45 %

Orbital 33 of fragment 1, Occ: 2.00000 Contribution: 4.26 %
Sum of values shown above: 93.45 %

Occupation number of orbital 76 of the complex: 2.00000000
Orbital 34 of fragment 3, Occ: 2.00000 Contribution: 1.37 %
Orbital 42 of fragment 3, Occ: 2.00000 Contribution: 75.45 %
Orbital 45 of fragment 3, Occ: 2.00000 Contribution: 13.12 %
Orbital 47 of fragment 3, Occ: 2.00000 Contribution: 1.04 %
Orbital 52 of fragment 3, Occ: 2.00000 Contribution: 5.05 %
Orbital 58 of fragment 3, Occ: 2.00000 Contribution: 1.64 %
Sum of values shown above: 97.68 %

Occupation number of orbital 77 of the complex: 2.00000000
Orbital 8 of fragment 2, Occ: 2.00000 Contribution: 8.69 %
Orbital 10 of fragment 2, Occ: 0.00000 Contribution: 3.55 %
Orbital 33 of fragment 3, Occ: 2.00000 Contribution: 1.01 %
Orbital 38 of fragment 3, Occ: 2.00000 Contribution: 5.93 %
Orbital 42 of fragment 3, Occ: 2.00000 Contribution: 6.27 %
Orbital 45 of fragment 3, Occ: 2.00000 Contribution: 8.33 %
Orbital 54 of fragment 3, Occ: 2.00000 Contribution: 2.01 %
Orbital 62 of fragment 3, Occ: 2.00000 Contribution: 4.73 %
Orbital 65 of fragment 3, Occ: 2.00000 Contribution: 7.46 %
Orbital 25 of fragment 1, Occ: 2.00000 Contribution: 1.18 %
Orbital 27 of fragment 1, Occ: 2.00000 Contribution: 34.65 %
Orbital 29 of fragment 1, Occ: 2.00000 Contribution: 4.49 %
Orbital 42 of fragment 1, Occ: 2.00000 Contribution: 3.49 %
Sum of values shown above: 91.78 %

Occupation number of orbital 78 of the complex: 2.00000000
Orbital 5 of fragment 2, Occ: 2.00000 Contribution: 3.28 %
Orbital 41 of fragment 3, Occ: 2.00000 Contribution: 1.57 %
Orbital 43 of fragment 3, Occ: 2.00000 Contribution: 32.93 %
Orbital 46 of fragment 3, Occ: 2.00000 Contribution: 42.25 %
Orbital 48 of fragment 3, Occ: 2.00000 Contribution: 4.77 %
Orbital 53 of fragment 3, Occ: 2.00000 Contribution: 1.74 %
Orbital 33 of fragment 1, Occ: 2.00000 Contribution: 7.92 %
Sum of values shown above: 94.47 %

Occupation number of orbital 79 of the complex: 2.00000000
Orbital 7 of fragment 2, Occ: 2.00000 Contribution: 1.27 %

Orbital 44 of fragment 3, Occ: 2.00000 Contribution: 1.19 %
Orbital 46 of fragment 3, Occ: 2.00000 Contribution: 1.42 %
Orbital 26 of fragment 1, Occ: 2.00000 Contribution: 6.06 %
Orbital 28 of fragment 1, Occ: 2.00000 Contribution: 72.91 %
Orbital 30 of fragment 1, Occ: 2.00000 Contribution: 10.39 %
Orbital 32 of fragment 1, Occ: 2.00000 Contribution: 3.08 %
Orbital 36 of fragment 1, Occ: 2.00000 Contribution: 1.09 %
Sum of values shown above: 97.40 %

Occupation number of orbital 80 of the complex: 2.00000000
Orbital 9 of fragment 2, Occ: 0.00000 Contribution: 1.73 %
Orbital 42 of fragment 3, Occ: 2.00000 Contribution: 1.52 %
Orbital 45 of fragment 3, Occ: 2.00000 Contribution: 5.40 %
Orbital 50 of fragment 3, Occ: 2.00000 Contribution: 2.90 %
Orbital 62 of fragment 3, Occ: 2.00000 Contribution: 1.38 %
Orbital 65 of fragment 3, Occ: 2.00000 Contribution: 1.17 %
Orbital 68 of fragment 3, Occ: 2.00000 Contribution: 1.23 %
Orbital 29 of fragment 1, Occ: 2.00000 Contribution: 81.05 %
Sum of values shown above: 96.39 %

Occupation number of orbital 81 of the complex: 2.00000000
Orbital 43 of fragment 3, Occ: 2.00000 Contribution: 4.09 %
Orbital 46 of fragment 3, Occ: 2.00000 Contribution: 3.70 %
Orbital 49 of fragment 3, Occ: 2.00000 Contribution: 86.16 %
Orbital 53 of fragment 3, Occ: 2.00000 Contribution: 1.48 %
Orbital 60 of fragment 3, Occ: 2.00000 Contribution: 2.11 %
Sum of values shown above: 97.54 %

Occupation number of orbital 82 of the complex: 2.00000000
Orbital 5 of fragment 2, Occ: 2.00000 Contribution: 1.56 %
Orbital 7 of fragment 2, Occ: 2.00000 Contribution: 3.52 %
Orbital 44 of fragment 3, Occ: 2.00000 Contribution: 1.49 %
Orbital 46 of fragment 3, Occ: 2.00000 Contribution: 18.01 %
Orbital 48 of fragment 3, Occ: 2.00000 Contribution: 1.83 %
Orbital 51 of fragment 3, Occ: 2.00000 Contribution: 2.15 %
Orbital 30 of fragment 1, Occ: 2.00000 Contribution: 1.91 %
Orbital 33 of fragment 1, Occ: 2.00000 Contribution: 59.91 %
Orbital 39 of fragment 1, Occ: 2.00000 Contribution: 3.30 %
Sum of values shown above: 93.66 %

Occupation number of orbital 83 of the complex: 2.00000000
Orbital 6 of fragment 2, Occ: 2.00000 Contribution: 1.48 %
Orbital 8 of fragment 2, Occ: 2.00000 Contribution: 6.69 %
Orbital 9 of fragment 2, Occ: 0.00000 Contribution: 7.69 %
Orbital 10 of fragment 2, Occ: 0.00000 Contribution: 1.92 %
Orbital 38 of fragment 3, Occ: 2.00000 Contribution: 2.73 %
Orbital 40 of fragment 3, Occ: 2.00000 Contribution: 1.44 %
Orbital 42 of fragment 3, Occ: 2.00000 Contribution: 4.93 %
Orbital 45 of fragment 3, Occ: 2.00000 Contribution: 35.35 %
Orbital 47 of fragment 3, Occ: 2.00000 Contribution: 5.17 %
Orbital 50 of fragment 3, Occ: 2.00000 Contribution: 1.11 %
Orbital 54 of fragment 3, Occ: 2.00000 Contribution: 16.15 %
Orbital 62 of fragment 3, Occ: 2.00000 Contribution: 1.16 %
Orbital 65 of fragment 3, Occ: 2.00000 Contribution: 1.54 %
Orbital 68 of fragment 3, Occ: 2.00000 Contribution: 5.60 %
Orbital 29 of fragment 1, Occ: 2.00000 Contribution: 4.16 %
Sum of values shown above: 97.11 %

Occupation number of orbital 84 of the complex: 2.00000000
Orbital 5 of fragment 2, Occ: 2.00000 Contribution: 1.08 %
Orbital 7 of fragment 2, Occ: 2.00000 Contribution: 1.60 %
Orbital 48 of fragment 3, Occ: 2.00000 Contribution: 2.37 %
Orbital 28 of fragment 1, Occ: 2.00000 Contribution: 16.92 %
Orbital 30 of fragment 1, Occ: 2.00000 Contribution: 70.40 %
Orbital 33 of fragment 1, Occ: 2.00000 Contribution: 1.43 %
Sum of values shown above: 93.79 %

Occupation number of orbital 85 of the complex: 2.00000000
Orbital 45 of fragment 3, Occ: 2.00000 Contribution: 1.10 %
Orbital 47 of fragment 3, Occ: 2.00000 Contribution: 1.14 %
Orbital 50 of fragment 3, Occ: 2.00000 Contribution: 86.07 %
Orbital 52 of fragment 3, Occ: 2.00000 Contribution: 2.16 %
Orbital 58 of fragment 3, Occ: 2.00000 Contribution: 2.16 %
Orbital 29 of fragment 1, Occ: 2.00000 Contribution: 3.54 %
Sum of values shown above: 96.17 %

Occupation number of orbital 86 of the complex: 2.00000000
Orbital 6 of fragment 2, Occ: 2.00000 Contribution: 7.58 %
Orbital 45 of fragment 3, Occ: 2.00000 Contribution: 5.26 %
Orbital 47 of fragment 3, Occ: 2.00000 Contribution: 67.09 %
Orbital 50 of fragment 3, Occ: 2.00000 Contribution: 1.99 %

Orbital 55 of fragment 3, Occ: 2.00000 Contribution: 11.34 %
Orbital 29 of fragment 1, Occ: 2.00000 Contribution: 1.27 %
Orbital 31 of fragment 1, Occ: 2.00000 Contribution: 1.63 %
Sum of values shown above: 96.16 %

Occupation number of orbital 87 of the complex: 2.00000000
Orbital 44 of fragment 3, Occ: 2.00000 Contribution: 3.31 %
Orbital 46 of fragment 3, Occ: 2.00000 Contribution: 10.30 %
Orbital 48 of fragment 3, Occ: 2.00000 Contribution: 80.82 %
Orbital 57 of fragment 3, Occ: 2.00000 Contribution: 1.71 %
Orbital 30 of fragment 1, Occ: 2.00000 Contribution: 1.69 %
Sum of values shown above: 97.82 %

Occupation number of orbital 88 of the complex: 2.00000000
Orbital 51 of fragment 3, Occ: 2.00000 Contribution: 1.18 %
Orbital 28 of fragment 1, Occ: 2.00000 Contribution: 3.27 %
Orbital 30 of fragment 1, Occ: 2.00000 Contribution: 4.11 %
Orbital 32 of fragment 1, Occ: 2.00000 Contribution: 86.86 %
Orbital 33 of fragment 1, Occ: 2.00000 Contribution: 1.09 %
Orbital 36 of fragment 1, Occ: 2.00000 Contribution: 1.25 %
Sum of values shown above: 97.76 %

Occupation number of orbital 89 of the complex: 2.00000000
Orbital 47 of fragment 3, Occ: 2.00000 Contribution: 2.95 %
Orbital 62 of fragment 3, Occ: 2.00000 Contribution: 1.13 %
Orbital 65 of fragment 3, Occ: 2.00000 Contribution: 1.19 %
Orbital 31 of fragment 1, Occ: 2.00000 Contribution: 90.08 %
Sum of values shown above: 95.34 %

Occupation number of orbital 90 of the complex: 2.00000000
Orbital 5 of fragment 2, Occ: 2.00000 Contribution: 12.93 %
Orbital 39 of fragment 3, Occ: 2.00000 Contribution: 1.17 %
Orbital 46 of fragment 3, Occ: 2.00000 Contribution: 3.09 %
Orbital 48 of fragment 3, Occ: 2.00000 Contribution: 2.04 %
Orbital 49 of fragment 3, Occ: 2.00000 Contribution: 1.10 %
Orbital 51 of fragment 3, Occ: 2.00000 Contribution: 64.69 %
Orbital 53 of fragment 3, Occ: 2.00000 Contribution: 2.93 %
Orbital 56 of fragment 3, Occ: 2.00000 Contribution: 1.66 %
Orbital 60 of fragment 3, Occ: 2.00000 Contribution: 3.97 %
Orbital 32 of fragment 1, Occ: 2.00000 Contribution: 2.42 %

Sum of values shown above: 96.00 %

Occupation number of orbital 91 of the complex: 2.00000000

Orbital	6 of fragment	2, Occ: 2.00000	Contribution: 36.87 %
Orbital	8 of fragment	2, Occ: 2.00000	Contribution: 2.11 %
Orbital	17 of fragment	2, Occ: 0.00000	Contribution: 1.41 %
Orbital	45 of fragment	3, Occ: 2.00000	Contribution: 1.53 %
Orbital	47 of fragment	3, Occ: 2.00000	Contribution: 18.83 %
Orbital	55 of fragment	3, Occ: 2.00000	Contribution: 28.28 %
Orbital	64 of fragment	3, Occ: 2.00000	Contribution: 1.73 %
Orbital	67 of fragment	3, Occ: 2.00000	Contribution: 2.02 %
Orbital	31 of fragment	1, Occ: 2.00000	Contribution: 3.05 %

Sum of values shown above: 95.82 %

Occupation number of orbital 92 of the complex: 2.00000000

Orbital	7 of fragment	2, Occ: 2.00000	Contribution: 31.04 %
Orbital	44 of fragment	3, Occ: 2.00000	Contribution: 14.15 %
Orbital	51 of fragment	3, Occ: 2.00000	Contribution: 1.90 %
Orbital	57 of fragment	3, Occ: 2.00000	Contribution: 20.98 %
Orbital	59 of fragment	3, Occ: 2.00000	Contribution: 11.93 %
Orbital	60 of fragment	3, Occ: 2.00000	Contribution: 1.93 %
Orbital	30 of fragment	1, Occ: 2.00000	Contribution: 2.72 %
Orbital	33 of fragment	1, Occ: 2.00000	Contribution: 7.78 %

Sum of values shown above: 92.43 %

Occupation number of orbital 93 of the complex: 2.00000000

Orbital	5 of fragment	2, Occ: 2.00000	Contribution: 30.90 %
Orbital	43 of fragment	3, Occ: 2.00000	Contribution: 6.84 %
Orbital	44 of fragment	3, Occ: 2.00000	Contribution: 1.37 %
Orbital	46 of fragment	3, Occ: 2.00000	Contribution: 2.53 %
Orbital	49 of fragment	3, Occ: 2.00000	Contribution: 4.15 %
Orbital	51 of fragment	3, Occ: 2.00000	Contribution: 9.41 %
Orbital	53 of fragment	3, Occ: 2.00000	Contribution: 10.39 %
Orbital	56 of fragment	3, Occ: 2.00000	Contribution: 12.09 %
Orbital	57 of fragment	3, Occ: 2.00000	Contribution: 2.82 %
Orbital	59 of fragment	3, Occ: 2.00000	Contribution: 2.80 %
Orbital	60 of fragment	3, Occ: 2.00000	Contribution: 2.97 %
Orbital	30 of fragment	1, Occ: 2.00000	Contribution: 2.06 %
Orbital	32 of fragment	1, Occ: 2.00000	Contribution: 1.19 %
Orbital	33 of fragment	1, Occ: 2.00000	Contribution: 4.80 %

Sum of values shown above: 94.32 %

Occupation number of orbital 94 of the complex: 2.00000000

Orbital 8 of fragment 2, Occ: 2.00000 Contribution: 2.45 %
Orbital 42 of fragment 3, Occ: 2.00000 Contribution: 3.76 %
Orbital 50 of fragment 3, Occ: 2.00000 Contribution: 1.42 %
Orbital 52 of fragment 3, Occ: 2.00000 Contribution: 61.23 %
Orbital 58 of fragment 3, Occ: 2.00000 Contribution: 4.54 %
Orbital 62 of fragment 3, Occ: 2.00000 Contribution: 1.39 %
Orbital 65 of fragment 3, Occ: 2.00000 Contribution: 1.64 %
Orbital 68 of fragment 3, Occ: 2.00000 Contribution: 1.29 %
Orbital 31 of fragment 1, Occ: 2.00000 Contribution: 1.24 %
Orbital 34 of fragment 1, Occ: 2.00000 Contribution: 9.73 %
Orbital 37 of fragment 1, Occ: 2.00000 Contribution: 2.85 %
Orbital 42 of fragment 1, Occ: 2.00000 Contribution: 4.53 %
Sum of values shown above: 96.07 %

Occupation number of orbital 95 of the complex: 2.00000000

Orbital 5 of fragment 2, Occ: 2.00000 Contribution: 4.61 %
Orbital 51 of fragment 3, Occ: 2.00000 Contribution: 9.06 %
Orbital 56 of fragment 3, Occ: 2.00000 Contribution: 73.66 %
Orbital 69 of fragment 3, Occ: 2.00000 Contribution: 1.17 %
Orbital 33 of fragment 1, Occ: 2.00000 Contribution: 5.24 %
Orbital 39 of fragment 1, Occ: 2.00000 Contribution: 1.09 %
Sum of values shown above: 94.84 %

Occupation number of orbital 96 of the complex: 2.00000000

Orbital 50 of fragment 3, Occ: 2.00000 Contribution: 2.00 %
Orbital 52 of fragment 3, Occ: 2.00000 Contribution: 21.47 %
Orbital 54 of fragment 3, Occ: 2.00000 Contribution: 2.65 %
Orbital 62 of fragment 3, Occ: 2.00000 Contribution: 12.17 %
Orbital 65 of fragment 3, Occ: 2.00000 Contribution: 7.39 %
Orbital 31 of fragment 1, Occ: 2.00000 Contribution: 1.70 %
Orbital 34 of fragment 1, Occ: 2.00000 Contribution: 28.69 %
Orbital 37 of fragment 1, Occ: 2.00000 Contribution: 6.53 %
Orbital 42 of fragment 1, Occ: 2.00000 Contribution: 8.26 %
Sum of values shown above: 90.88 %

Occupation number of orbital 97 of the complex: 2.00000000

Orbital 5 of fragment 2, Occ: 2.00000 Contribution: 6.78 %
Orbital 43 of fragment 3, Occ: 2.00000 Contribution: 3.32 %

Orbital 49 of fragment 3, Occ: 2.00000 Contribution: 1.65 %
Orbital 51 of fragment 3, Occ: 2.00000 Contribution: 7.04 %
Orbital 53 of fragment 3, Occ: 2.00000 Contribution: 73.01 %
Orbital 56 of fragment 3, Occ: 2.00000 Contribution: 4.44 %
Sum of values shown above: 96.25 %

Occupation number of orbital 98 of the complex: 2.00000000
Orbital 7 of fragment 2, Occ: 2.00000 Contribution: 14.47 %
Orbital 48 of fragment 3, Occ: 2.00000 Contribution: 1.82 %
Orbital 57 of fragment 3, Occ: 2.00000 Contribution: 64.85 %
Orbital 59 of fragment 3, Occ: 2.00000 Contribution: 4.83 %
Orbital 66 of fragment 3, Occ: 2.00000 Contribution: 1.91 %
Orbital 30 of fragment 1, Occ: 2.00000 Contribution: 1.89 %
Orbital 36 of fragment 1, Occ: 2.00000 Contribution: 5.62 %
Sum of values shown above: 95.40 %

Occupation number of orbital 99 of the complex: 2.00000000
Orbital 8 of fragment 2, Occ: 2.00000 Contribution: 10.61 %
Orbital 9 of fragment 2, Occ: 0.00000 Contribution: 2.37 %
Orbital 45 of fragment 3, Occ: 2.00000 Contribution: 3.48 %
Orbital 52 of fragment 3, Occ: 2.00000 Contribution: 2.72 %
Orbital 54 of fragment 3, Occ: 2.00000 Contribution: 56.54 %
Orbital 58 of fragment 3, Occ: 2.00000 Contribution: 16.21 %
Orbital 65 of fragment 3, Occ: 2.00000 Contribution: 2.06 %
Orbital 68 of fragment 3, Occ: 2.00000 Contribution: 1.81 %
Sum of values shown above: 95.80 %

Occupation number of orbital 100 of the complex: 2.00000000
Orbital 6 of fragment 2, Occ: 2.00000 Contribution: 35.96 %
Orbital 8 of fragment 2, Occ: 2.00000 Contribution: 1.86 %
Orbital 55 of fragment 3, Occ: 2.00000 Contribution: 54.97 %
Orbital 61 of fragment 3, Occ: 2.00000 Contribution: 1.64 %
Sum of values shown above: 94.42 %

Occupation number of orbital 101 of the complex: 2.00000000
Orbital 57 of fragment 3, Occ: 2.00000 Contribution: 2.93 %
Orbital 59 of fragment 3, Occ: 2.00000 Contribution: 7.59 %
Orbital 32 of fragment 1, Occ: 2.00000 Contribution: 1.60 %
Orbital 36 of fragment 1, Occ: 2.00000 Contribution: 84.32 %
Sum of values shown above: 96.44 %

Occupation number of orbital 102 of the complex: 2.00000000
Orbital 52 of fragment 3, Occ: 2.00000 Contribution: 1.77 %
Orbital 54 of fragment 3, Occ: 2.00000 Contribution: 2.14 %
Orbital 58 of fragment 3, Occ: 2.00000 Contribution: 9.29 %
Orbital 34 of fragment 1, Occ: 2.00000 Contribution: 3.71 %
Orbital 35 of fragment 1, Occ: 2.00000 Contribution: 78.50 %
Orbital 37 of fragment 1, Occ: 2.00000 Contribution: 1.46 %
Sum of values shown above: 96.86 %

Occupation number of orbital 103 of the complex: 2.00000000
Orbital 6 of fragment 2, Occ: 2.00000 Contribution: 1.25 %
Orbital 62 of fragment 3, Occ: 2.00000 Contribution: 4.26 %
Orbital 65 of fragment 3, Occ: 2.00000 Contribution: 1.61 %
Orbital 34 of fragment 1, Occ: 2.00000 Contribution: 48.06 %
Orbital 35 of fragment 1, Occ: 2.00000 Contribution: 4.04 %
Orbital 37 of fragment 1, Occ: 2.00000 Contribution: 28.25 %
Orbital 42 of fragment 1, Occ: 2.00000 Contribution: 7.22 %
Sum of values shown above: 94.70 %

Occupation number of orbital 104 of the complex: 2.00000000
Orbital 6 of fragment 2, Occ: 2.00000 Contribution: 1.26 %
Orbital 8 of fragment 2, Occ: 2.00000 Contribution: 8.90 %
Orbital 10 of fragment 2, Occ: 0.00000 Contribution: 1.26 %
Orbital 52 of fragment 3, Occ: 2.00000 Contribution: 3.70 %
Orbital 54 of fragment 3, Occ: 2.00000 Contribution: 7.36 %
Orbital 58 of fragment 3, Occ: 2.00000 Contribution: 57.76 %
Orbital 62 of fragment 3, Occ: 2.00000 Contribution: 1.48 %
Orbital 35 of fragment 1, Occ: 2.00000 Contribution: 14.82 %
Sum of values shown above: 96.54 %

Occupation number of orbital 105 of the complex: 2.00000000
Orbital 5 of fragment 2, Occ: 2.00000 Contribution: 1.42 %
Orbital 7 of fragment 2, Occ: 2.00000 Contribution: 5.13 %
Orbital 51 of fragment 3, Occ: 2.00000 Contribution: 1.76 %
Orbital 53 of fragment 3, Occ: 2.00000 Contribution: 1.23 %
Orbital 59 of fragment 3, Occ: 2.00000 Contribution: 6.60 %
Orbital 60 of fragment 3, Occ: 2.00000 Contribution: 63.56 %
Orbital 33 of fragment 1, Occ: 2.00000 Contribution: 3.40 %
Orbital 39 of fragment 1, Occ: 2.00000 Contribution: 11.15 %

Orbital 40 of fragment 1, Occ: 2.00000 Contribution: 1.17 %
Sum of values shown above: 95.41 %

Occupation number of orbital 106 of the complex: 2.00000000
Orbital 5 of fragment 2, Occ: 2.00000 Contribution: 6.52 %
Orbital 7 of fragment 2, Occ: 2.00000 Contribution: 17.35 %
Orbital 59 of fragment 3, Occ: 2.00000 Contribution: 49.89 %
Orbital 60 of fragment 3, Occ: 2.00000 Contribution: 11.19 %
Orbital 66 of fragment 3, Occ: 2.00000 Contribution: 1.13 %
Orbital 36 of fragment 1, Occ: 2.00000 Contribution: 4.92 %
Orbital 38 of fragment 1, Occ: 2.00000 Contribution: 6.54 %
Sum of values shown above: 97.53 %

Occupation number of orbital 107 of the complex: 2.00000000
Orbital 5 of fragment 2, Occ: 2.00000 Contribution: 1.11 %
Orbital 7 of fragment 2, Occ: 2.00000 Contribution: 2.58 %
Orbital 59 of fragment 3, Occ: 2.00000 Contribution: 4.25 %
Orbital 38 of fragment 1, Occ: 2.00000 Contribution: 90.94 %
Sum of values shown above: 98.89 %

Occupation number of orbital 108 of the complex: 2.00000000
Orbital 6 of fragment 2, Occ: 2.00000 Contribution: 1.36 %
Orbital 8 of fragment 2, Occ: 2.00000 Contribution: 25.86 %
Orbital 9 of fragment 2, Occ: 0.00000 Contribution: 3.35 %
Orbital 10 of fragment 2, Occ: 0.00000 Contribution: 14.18 %
Orbital 54 of fragment 3, Occ: 2.00000 Contribution: 4.75 %
Orbital 58 of fragment 3, Occ: 2.00000 Contribution: 1.53 %
Orbital 62 of fragment 3, Occ: 2.00000 Contribution: 10.55 %
Orbital 65 of fragment 3, Occ: 2.00000 Contribution: 4.07 %
Orbital 68 of fragment 3, Occ: 2.00000 Contribution: 2.59 %
Orbital 34 of fragment 1, Occ: 2.00000 Contribution: 3.79 %
Orbital 37 of fragment 1, Occ: 2.00000 Contribution: 24.40 %
Orbital 42 of fragment 1, Occ: 2.00000 Contribution: 2.32 %
Sum of values shown above: 98.73 %

Occupation number of orbital 109 of the complex: 2.00000000
Orbital 8 of fragment 2, Occ: 2.00000 Contribution: 10.89 %
Orbital 10 of fragment 2, Occ: 0.00000 Contribution: 14.73 %
Orbital 54 of fragment 3, Occ: 2.00000 Contribution: 2.59 %
Orbital 68 of fragment 3, Occ: 2.00000 Contribution: 5.17 %

Orbital 34 of fragment 1, Occ: 2.00000 Contribution: 1.49 %
Orbital 37 of fragment 1, Occ: 2.00000 Contribution: 29.68 %
Orbital 42 of fragment 1, Occ: 2.00000 Contribution: 31.92 %
Sum of values shown above: 96.49 %

Occupation number of orbital 110 of the complex: 2.00000000
Orbital 5 of fragment 2, Occ: 2.00000 Contribution: 5.77 %
Orbital 7 of fragment 2, Occ: 2.00000 Contribution: 6.38 %
Orbital 60 of fragment 3, Occ: 2.00000 Contribution: 8.16 %
Orbital 69 of fragment 3, Occ: 2.00000 Contribution: 1.63 %
Orbital 39 of fragment 1, Occ: 2.00000 Contribution: 73.45 %
Sum of values shown above: 95.40 %

Occupation number of orbital 111 of the complex: 2.00000000
Orbital 55 of fragment 3, Occ: 2.00000 Contribution: 1.98 %
Orbital 61 of fragment 3, Occ: 2.00000 Contribution: 96.29 %
Sum of values shown above: 98.27 %

Occupation number of orbital 112 of the complex: 2.00000000
Orbital 58 of fragment 3, Occ: 2.00000 Contribution: 1.58 %
Orbital 62 of fragment 3, Occ: 2.00000 Contribution: 48.09 %
Orbital 65 of fragment 3, Occ: 2.00000 Contribution: 46.17 %
Orbital 42 of fragment 1, Occ: 2.00000 Contribution: 2.22 %
Sum of values shown above: 98.07 %

Occupation number of orbital 113 of the complex: 2.00000000
Orbital 39 of fragment 1, Occ: 2.00000 Contribution: 1.96 %
Orbital 40 of fragment 1, Occ: 2.00000 Contribution: 94.72 %
Sum of values shown above: 96.68 %

Occupation number of orbital 114 of the complex: 2.00000000
Orbital 13 of fragment 2, Occ: 0.00000 Contribution: 1.10 %
Orbital 57 of fragment 3, Occ: 2.00000 Contribution: 1.07 %
Orbital 63 of fragment 3, Occ: 2.00000 Contribution: 72.00 %
Orbital 66 of fragment 3, Occ: 2.00000 Contribution: 21.46 %
Sum of values shown above: 95.63 %

Occupation number of orbital 115 of the complex: 2.00000000

Orbital 6 of fragment 2, Occ: 2.00000 Contribution: 2.50 %
Orbital 64 of fragment 3, Occ: 2.00000 Contribution: 43.59 %
Orbital 41 of fragment 1, Occ: 2.00000 Contribution: 51.73 %
Sum of values shown above: 97.82 %

Occupation number of orbital 116 of the complex: 2.00000000
Orbital 59 of fragment 3, Occ: 2.00000 Contribution: 3.24 %
Orbital 63 of fragment 3, Occ: 2.00000 Contribution: 22.73 %
Orbital 66 of fragment 3, Occ: 2.00000 Contribution: 71.03 %
Sum of values shown above: 97.00 %

Occupation number of orbital 117 of the complex: 2.00000000
Orbital 6 of fragment 2, Occ: 2.00000 Contribution: 2.34 %
Orbital 64 of fragment 3, Occ: 2.00000 Contribution: 49.29 %
Orbital 41 of fragment 1, Occ: 2.00000 Contribution: 46.51 %
Sum of values shown above: 98.15 %

Occupation number of orbital 118 of the complex: 2.00000000
Orbital 5 of fragment 2, Occ: 2.00000 Contribution: 1.90 %
Orbital 11 of fragment 2, Occ: 0.00000 Contribution: 1.93 %
Orbital 20 of fragment 2, Occ: 0.00000 Contribution: 1.08 %
Orbital 56 of fragment 3, Occ: 2.00000 Contribution: 2.27 %
Orbital 69 of fragment 3, Occ: 2.00000 Contribution: 81.33 %
Orbital 39 of fragment 1, Occ: 2.00000 Contribution: 2.46 %
Orbital 40 of fragment 1, Occ: 2.00000 Contribution: 1.59 %
Orbital 44 of fragment 1, Occ: 2.00000 Contribution: 3.23 %
Sum of values shown above: 95.79 %

Occupation number of orbital 119 of the complex: 2.00000000
Orbital 6 of fragment 2, Occ: 2.00000 Contribution: 2.09 %
Orbital 64 of fragment 3, Occ: 2.00000 Contribution: 2.47 %
Orbital 67 of fragment 3, Occ: 2.00000 Contribution: 94.64 %
Sum of values shown above: 99.20 %

Occupation number of orbital 120 of the complex: 2.00000000
Orbital 43 of fragment 1, Occ: 2.00000 Contribution: 98.80 %
Sum of values shown above: 98.80 %

Occupation number of orbital 121 of the complex: 2.00000000

Orbital 5 of fragment 2, Occ: 2.00000 Contribution: 1.77 %
Orbital 69 of fragment 3, Occ: 2.00000 Contribution: 4.12 %
Orbital 39 of fragment 1, Occ: 2.00000 Contribution: 1.52 %
Orbital 44 of fragment 1, Occ: 2.00000 Contribution: 89.70 %
Sum of values shown above: 97.11 %

Occupation number of orbital 122 of the complex: 0.00000000

Orbital 7 of fragment 2, Occ: 2.00000 Contribution: 1.25 %
Orbital 13 of fragment 2, Occ: 0.00000 Contribution: 4.62 %
Orbital 63 of fragment 3, Occ: 2.00000 Contribution: 1.33 %
Orbital 71 of fragment 3, Occ: 0.00000 Contribution: 88.88 %
Sum of values shown above: 96.08 %

Occupation number of orbital 123 of the complex: 0.00000000

Orbital 70 of fragment 3, Occ: 0.00000 Contribution: 97.98 %
Sum of values shown above: 97.98 %

Occupation number of orbital 124 of the complex: 0.00000000

Orbital 9 of fragment 2, Occ: 0.00000 Contribution: 38.38 %
Orbital 10 of fragment 2, Occ: 0.00000 Contribution: 2.51 %
Orbital 16 of fragment 2, Occ: 0.00000 Contribution: -6.90 %
Orbital 62 of fragment 3, Occ: 2.00000 Contribution: 1.25 %
Orbital 65 of fragment 3, Occ: 2.00000 Contribution: 3.58 %
Orbital 68 of fragment 3, Occ: 2.00000 Contribution: 42.93 %
Orbital 42 of fragment 1, Occ: 2.00000 Contribution: 14.71 %
Sum of values shown above: 96.46 %

Occupation number of orbital 125 of the complex: 0.00000000

Orbital 13 of fragment 2, Occ: 0.00000 Contribution: 2.19 %
Orbital 72 of fragment 3, Occ: 0.00000 Contribution: 7.51 %
Orbital 74 of fragment 3, Occ: 0.00000 Contribution: 2.09 %
Orbital 45 of fragment 1, Occ: 0.00000 Contribution: 87.09 %
Sum of values shown above: 98.87 %

Occupation number of orbital 126 of the complex: 0.00000000

Orbital 13 of fragment 2, Occ: 0.00000 Contribution: 7.92 %
Orbital 66 of fragment 3, Occ: 2.00000 Contribution: 1.81 %
Orbital 71 of fragment 3, Occ: 0.00000 Contribution: 2.58 %

Orbital 72 of fragment 3, Occ: 0.00000 Contribution: 34.20 %
Orbital 74 of fragment 3, Occ: 0.00000 Contribution: 39.08 %
Orbital 45 of fragment 1, Occ: 0.00000 Contribution: 10.94 %
Sum of values shown above: 96.53 %

Occupation number of orbital 127 of the complex: 0.00000000
Orbital 13 of fragment 2, Occ: 0.00000 Contribution: 3.68 %
Orbital 72 of fragment 3, Occ: 0.00000 Contribution: 55.99 %
Orbital 74 of fragment 3, Occ: 0.00000 Contribution: 35.67 %
Sum of values shown above: 95.34 %

Occupation number of orbital 128 of the complex: 0.00000000
Orbital 6 of fragment 2, Occ: 2.00000 Contribution: 1.71 %
Orbital 73 of fragment 3, Occ: 0.00000 Contribution: 88.96 %
Orbital 75 of fragment 3, Occ: 0.00000 Contribution: 2.33 %
Orbital 46 of fragment 1, Occ: 0.00000 Contribution: 4.82 %
Sum of values shown above: 97.82 %

Occupation number of orbital 129 of the complex: 0.00000000
Orbital 73 of fragment 3, Occ: 0.00000 Contribution: 5.07 %
Orbital 46 of fragment 1, Occ: 0.00000 Contribution: 93.91 %
Sum of values shown above: 98.99 %

Occupation number of orbital 130 of the complex: 0.00000000
Orbital 73 of fragment 3, Occ: 0.00000 Contribution: 1.95 %
Orbital 75 of fragment 3, Occ: 0.00000 Contribution: 85.82 %
Orbital 85 of fragment 3, Occ: 0.00000 Contribution: 1.18 %
Orbital 47 of fragment 1, Occ: 0.00000 Contribution: 8.27 %
Sum of values shown above: 97.22 %

Occupation number of orbital 131 of the complex: 0.00000000
Orbital 13 of fragment 2, Occ: 0.00000 Contribution: 3.10 %
Orbital 74 of fragment 3, Occ: 0.00000 Contribution: 2.48 %
Orbital 44 of fragment 1, Occ: 2.00000 Contribution: 1.24 %
Orbital 48 of fragment 1, Occ: 0.00000 Contribution: 89.05 %
Orbital 56 of fragment 1, Occ: 0.00000 Contribution: 1.91 %
Sum of values shown above: 97.78 %

Occupation number of orbital 132 of the complex: 0.00000000
Orbital 75 of fragment 3, Occ: 0.00000 Contribution: 8.57 %
Orbital 47 of fragment 1, Occ: 0.00000 Contribution: 89.25 %
Sum of values shown above: 97.82 %

Occupation number of orbital 133 of the complex: 0.00000000
Orbital 11 of fragment 2, Occ: 0.00000 Contribution: 1.86 %
Orbital 13 of fragment 2, Occ: 0.00000 Contribution: 58.93 %
Orbital 71 of fragment 3, Occ: 0.00000 Contribution: 2.89 %
Orbital 74 of fragment 3, Occ: 0.00000 Contribution: 16.56 %
Orbital 82 of fragment 3, Occ: 0.00000 Contribution: 1.87 %
Orbital 88 of fragment 3, Occ: 0.00000 Contribution: 1.06 %
Orbital 48 of fragment 1, Occ: 0.00000 Contribution: 5.52 %
Orbital 50 of fragment 1, Occ: 0.00000 Contribution: 9.20 %
Orbital 52 of fragment 1, Occ: 0.00000 Contribution: 1.16 %
Sum of values shown above: 99.04 %

Occupation number of orbital 134 of the complex: 0.00000000
Orbital 8 of fragment 2, Occ: 2.00000 Contribution: 3.67 %
Orbital 10 of fragment 2, Occ: 0.00000 Contribution: 16.93 %
Orbital 12 of fragment 2, Occ: 0.00000 Contribution: 8.88 %
Orbital 14 of fragment 2, Occ: 0.00000 Contribution: 50.84 %
Orbital 19 of fragment 2, Occ: 0.00000 Contribution: -5.43 %
Orbital 21 of fragment 2, Occ: 0.00000 Contribution: 1.29 %
Orbital 30 of fragment 2, Occ: 0.00000 Contribution: -1.35 %
Orbital 65 of fragment 3, Occ: 2.00000 Contribution: 2.40 %
Orbital 68 of fragment 3, Occ: 2.00000 Contribution: 5.62 %
Orbital 76 of fragment 3, Occ: 0.00000 Contribution: 13.91 %
Orbital 78 of fragment 3, Occ: 0.00000 Contribution: 2.79 %
Orbital 91 of fragment 3, Occ: 0.00000 Contribution: -1.46 %
Orbital 96 of fragment 3, Occ: 0.00000 Contribution: -3.80 %
Orbital 99 of fragment 3, Occ: 0.00000 Contribution: -2.78 %
Orbital 101 of fragment 3, Occ: 0.00000 Contribution: -1.39 %
Orbital 130 of fragment 3, Occ: 0.00000 Contribution: -1.62 %
Orbital 49 of fragment 1, Occ: 0.00000 Contribution: 7.05 %
Orbital 51 of fragment 1, Occ: 0.00000 Contribution: 5.43 %
Orbital 53 of fragment 1, Occ: 0.00000 Contribution: 1.51 %
Sum of values shown above: 102.50 %

Occupation number of orbital 135 of the complex: 0.00000000
Orbital 12 of fragment 2, Occ: 0.00000 Contribution: 6.67 %

Orbital 14 of fragment 2, Occ: 0.00000 Contribution: 3.13 %
Orbital 76 of fragment 3, Occ: 0.00000 Contribution: 43.24 %
Orbital 78 of fragment 3, Occ: 0.00000 Contribution: 18.03 %
Orbital 49 of fragment 1, Occ: 0.00000 Contribution: 28.39 %
Sum of values shown above: 99.45 %

Occupation number of orbital 136 of the complex: 0.00000000
Orbital 11 of fragment 2, Occ: 0.00000 Contribution: 6.65 %
Orbital 77 of fragment 3, Occ: 0.00000 Contribution: 79.82 %
Orbital 79 of fragment 3, Occ: 0.00000 Contribution: 8.89 %
Orbital 50 of fragment 1, Occ: 0.00000 Contribution: 3.79 %
Sum of values shown above: 99.14 %

Occupation number of orbital 137 of the complex: 0.00000000
Orbital 10 of fragment 2, Occ: 0.00000 Contribution: 1.35 %
Orbital 14 of fragment 2, Occ: 0.00000 Contribution: 5.62 %
Orbital 76 of fragment 3, Occ: 0.00000 Contribution: 1.43 %
Orbital 78 of fragment 3, Occ: 0.00000 Contribution: 61.32 %
Orbital 83 of fragment 3, Occ: 0.00000 Contribution: 2.16 %
Orbital 49 of fragment 1, Occ: 0.00000 Contribution: 24.17 %
Orbital 51 of fragment 1, Occ: 0.00000 Contribution: 2.46 %
Sum of values shown above: 98.52 %

Occupation number of orbital 138 of the complex: 0.00000000
Orbital 10 of fragment 2, Occ: 0.00000 Contribution: 2.15 %
Orbital 12 of fragment 2, Occ: 0.00000 Contribution: 8.08 %
Orbital 14 of fragment 2, Occ: 0.00000 Contribution: 4.33 %
Orbital 21 of fragment 2, Occ: 0.00000 Contribution: 1.05 %
Orbital 68 of fragment 3, Occ: 2.00000 Contribution: 1.35 %
Orbital 76 of fragment 3, Occ: 0.00000 Contribution: 32.25 %
Orbital 78 of fragment 3, Occ: 0.00000 Contribution: 13.69 %
Orbital 49 of fragment 1, Occ: 0.00000 Contribution: 37.17 %
Orbital 51 of fragment 1, Occ: 0.00000 Contribution: 2.16 %
Sum of values shown above: 102.24 %

Occupation number of orbital 139 of the complex: 0.00000000
Orbital 11 of fragment 2, Occ: 0.00000 Contribution: 3.62 %
Orbital 13 of fragment 2, Occ: 0.00000 Contribution: 2.54 %
Orbital 79 of fragment 3, Occ: 0.00000 Contribution: 60.92 %
Orbital 82 of fragment 3, Occ: 0.00000 Contribution: 1.46 %

Orbital 50 of fragment 1, Occ: 0.00000 Contribution: 29.05 %
Sum of values shown above: 97.59 %

Occupation number of orbital 140 of the complex: 0.00000000
Orbital 13 of fragment 2, Occ: 0.00000 Contribution: 1.51 %
Orbital 77 of fragment 3, Occ: 0.00000 Contribution: 13.06 %
Orbital 79 of fragment 3, Occ: 0.00000 Contribution: 28.71 %
Orbital 82 of fragment 3, Occ: 0.00000 Contribution: 5.82 %
Orbital 84 of fragment 3, Occ: 0.00000 Contribution: 1.43 %
Orbital 88 of fragment 3, Occ: 0.00000 Contribution: 1.26 %
Orbital 50 of fragment 1, Occ: 0.00000 Contribution: 43.70 %
Orbital 52 of fragment 1, Occ: 0.00000 Contribution: 2.23 %
Sum of values shown above: 97.72 %

Occupation number of orbital 141 of the complex: 0.00000000
Orbital 11 of fragment 2, Occ: 0.00000 Contribution: 6.45 %
Orbital 13 of fragment 2, Occ: 0.00000 Contribution: 9.38 %
Orbital 22 of fragment 2, Occ: 0.00000 Contribution: 1.02 %
Orbital 77 of fragment 3, Occ: 0.00000 Contribution: 1.35 %
Orbital 81 of fragment 3, Occ: 0.00000 Contribution: 9.36 %
Orbital 82 of fragment 3, Occ: 0.00000 Contribution: 50.64 %
Orbital 84 of fragment 3, Occ: 0.00000 Contribution: 7.17 %
Orbital 88 of fragment 3, Occ: 0.00000 Contribution: 10.90 %
Orbital 50 of fragment 1, Occ: 0.00000 Contribution: 2.17 %
Orbital 52 of fragment 1, Occ: 0.00000 Contribution: 1.37 %
Sum of values shown above: 99.80 %

Occupation number of orbital 142 of the complex: 0.00000000
Orbital 10 of fragment 2, Occ: 0.00000 Contribution: 5.46 %
Orbital 12 of fragment 2, Occ: 0.00000 Contribution: 35.36 %
Orbital 14 of fragment 2, Occ: 0.00000 Contribution: 3.42 %
Orbital 21 of fragment 2, Occ: 0.00000 Contribution: 3.51 %
Orbital 76 of fragment 3, Occ: 0.00000 Contribution: 6.92 %
Orbital 78 of fragment 3, Occ: 0.00000 Contribution: 1.26 %
Orbital 83 of fragment 3, Occ: 0.00000 Contribution: 34.64 %
Orbital 85 of fragment 3, Occ: 0.00000 Contribution: 1.04 %
Orbital 42 of fragment 1, Occ: 2.00000 Contribution: 1.47 %
Orbital 49 of fragment 1, Occ: 0.00000 Contribution: 1.44 %
Orbital 51 of fragment 1, Occ: 0.00000 Contribution: 7.73 %
Orbital 65 of fragment 1, Occ: 0.00000 Contribution: -1.00 %
Sum of values shown above: 101.23 %

Occupation number of orbital 143 of the complex: 0.00000000

Orbital 11 of fragment	2, Occ: 0.00000	Contribution:	42.19 %
Orbital 20 of fragment	2, Occ: 0.00000	Contribution:	5.33 %
Orbital 77 of fragment	3, Occ: 0.00000	Contribution:	2.26 %
Orbital 81 of fragment	3, Occ: 0.00000	Contribution:	29.51 %
Orbital 82 of fragment	3, Occ: 0.00000	Contribution:	8.06 %
Orbital 103 of fragment	3, Occ: 0.00000	Contribution:	-1.09 %
Orbital 108 of fragment	3, Occ: 0.00000	Contribution:	-1.83 %
Orbital 113 of fragment	3, Occ: 0.00000	Contribution:	-1.59 %
Orbital 138 of fragment	3, Occ: 0.00000	Contribution:	-1.16 %
Orbital 50 of fragment	1, Occ: 0.00000	Contribution:	3.84 %
Orbital 52 of fragment	1, Occ: 0.00000	Contribution:	17.24 %
Orbital 56 of fragment	1, Occ: 0.00000	Contribution:	1.52 %

Sum of values shown above: 104.29 %

Occupation number of orbital 144 of the complex: 0.00000000

Orbital 12 of fragment	2, Occ: 0.00000	Contribution:	10.53 %
Orbital 14 of fragment	2, Occ: 0.00000	Contribution:	1.76 %
Orbital 21 of fragment	2, Occ: 0.00000	Contribution:	1.01 %
Orbital 80 of fragment	3, Occ: 0.00000	Contribution:	1.80 %
Orbital 83 of fragment	3, Occ: 0.00000	Contribution:	1.42 %
Orbital 89 of fragment	3, Occ: 0.00000	Contribution:	3.31 %
Orbital 51 of fragment	1, Occ: 0.00000	Contribution:	72.95 %
Orbital 53 of fragment	1, Occ: 0.00000	Contribution:	4.63 %
Orbital 55 of fragment	1, Occ: 0.00000	Contribution:	1.98 %

Sum of values shown above: 99.39 %

Occupation number of orbital 145 of the complex: 0.00000000

Orbital 80 of fragment	3, Occ: 0.00000	Contribution:	94.81 %
Orbital 51 of fragment	1, Occ: 0.00000	Contribution:	1.73 %

Sum of values shown above: 96.54 %

Occupation number of orbital 146 of the complex: 0.00000000

Orbital 11 of fragment	2, Occ: 0.00000	Contribution:	26.06 %
Orbital 20 of fragment	2, Occ: 0.00000	Contribution:	2.50 %
Orbital 77 of fragment	3, Occ: 0.00000	Contribution:	1.97 %
Orbital 81 of fragment	3, Occ: 0.00000	Contribution:	56.02 %
Orbital 84 of fragment	3, Occ: 0.00000	Contribution:	5.65 %
Orbital 87 of fragment	3, Occ: 0.00000	Contribution:	1.56 %

Orbital 103 of fragment 3, Occ: 0.00000 Contribution: -1.02 %
Orbital 52 of fragment 1, Occ: 0.00000 Contribution: 8.61 %
Orbital 56 of fragment 1, Occ: 0.00000 Contribution: 1.69 %
Sum of values shown above: 103.04 %

Occupation number of orbital 147 of the complex: 0.00000000
Orbital 11 of fragment 2, Occ: 0.00000 Contribution: -1.51 %
Orbital 81 of fragment 3, Occ: 0.00000 Contribution: 1.10 %
Orbital 82 of fragment 3, Occ: 0.00000 Contribution: 18.80 %
Orbital 84 of fragment 3, Occ: 0.00000 Contribution: 63.58 %
Orbital 88 of fragment 3, Occ: 0.00000 Contribution: 2.10 %
Orbital 52 of fragment 1, Occ: 0.00000 Contribution: 8.35 %
Orbital 56 of fragment 1, Occ: 0.00000 Contribution: 1.23 %
Sum of values shown above: 93.66 %

Occupation number of orbital 148 of the complex: 0.00000000
Orbital 12 of fragment 2, Occ: 0.00000 Contribution: 3.10 %
Orbital 75 of fragment 3, Occ: 0.00000 Contribution: 1.13 %
Orbital 83 of fragment 3, Occ: 0.00000 Contribution: 8.84 %
Orbital 85 of fragment 3, Occ: 0.00000 Contribution: 70.39 %
Orbital 89 of fragment 3, Occ: 0.00000 Contribution: 1.56 %
Orbital 92 of fragment 3, Occ: 0.00000 Contribution: 8.83 %
Orbital 53 of fragment 1, Occ: 0.00000 Contribution: 3.25 %
Sum of values shown above: 97.10 %

Occupation number of orbital 149 of the complex: 0.00000000
Orbital 85 of fragment 3, Occ: 0.00000 Contribution: 3.66 %
Orbital 89 of fragment 3, Occ: 0.00000 Contribution: 8.84 %
Orbital 53 of fragment 1, Occ: 0.00000 Contribution: 76.18 %
Orbital 55 of fragment 1, Occ: 0.00000 Contribution: 5.41 %
Sum of values shown above: 94.09 %

Occupation number of orbital 150 of the complex: 0.00000000
Orbital 11 of fragment 2, Occ: 0.00000 Contribution: 11.91 %
Orbital 20 of fragment 2, Occ: 0.00000 Contribution: 1.26 %
Orbital 84 of fragment 3, Occ: 0.00000 Contribution: 2.20 %
Orbital 88 of fragment 3, Occ: 0.00000 Contribution: 7.70 %
Orbital 93 of fragment 3, Occ: 0.00000 Contribution: 1.12 %
Orbital 50 of fragment 1, Occ: 0.00000 Contribution: 1.85 %
Orbital 52 of fragment 1, Occ: 0.00000 Contribution: 36.32 %

Orbital 54 of fragment 1, Occ: 0.00000 Contribution: 35.46 %
Orbital 56 of fragment 1, Occ: 0.00000 Contribution: 3.04 %
Sum of values shown above: 100.85 %

Occupation number of orbital 151 of the complex: 0.00000000
Orbital 11 of fragment 2, Occ: 0.00000 Contribution: 2.79 %
Orbital 81 of fragment 3, Occ: 0.00000 Contribution: 1.64 %
Orbital 82 of fragment 3, Occ: 0.00000 Contribution: 5.67 %
Orbital 84 of fragment 3, Occ: 0.00000 Contribution: 10.32 %
Orbital 87 of fragment 3, Occ: 0.00000 Contribution: 10.63 %
Orbital 88 of fragment 3, Occ: 0.00000 Contribution: 53.03 %
Orbital 93 of fragment 3, Occ: 0.00000 Contribution: 9.46 %
Orbital 52 of fragment 1, Occ: 0.00000 Contribution: 1.28 %
Orbital 54 of fragment 1, Occ: 0.00000 Contribution: 3.47 %
Sum of values shown above: 98.28 %

Occupation number of orbital 152 of the complex: 0.00000000
Orbital 14 of fragment 2, Occ: 0.00000 Contribution: 1.56 %
Orbital 83 of fragment 3, Occ: 0.00000 Contribution: 3.46 %
Orbital 85 of fragment 3, Occ: 0.00000 Contribution: 2.17 %
Orbital 86 of fragment 3, Occ: 0.00000 Contribution: 85.79 %
Orbital 89 of fragment 3, Occ: 0.00000 Contribution: 3.92 %
Sum of values shown above: 96.90 %

Occupation number of orbital 153 of the complex: 0.00000000
Orbital 14 of fragment 2, Occ: 0.00000 Contribution: 2.58 %
Orbital 83 of fragment 3, Occ: 0.00000 Contribution: 5.10 %
Orbital 85 of fragment 3, Occ: 0.00000 Contribution: 3.16 %
Orbital 86 of fragment 3, Occ: 0.00000 Contribution: 7.37 %
Orbital 89 of fragment 3, Occ: 0.00000 Contribution: 47.29 %
Orbital 92 of fragment 3, Occ: 0.00000 Contribution: 1.43 %
Orbital 96 of fragment 3, Occ: 0.00000 Contribution: 1.70 %
Orbital 53 of fragment 1, Occ: 0.00000 Contribution: 2.55 %
Orbital 55 of fragment 1, Occ: 0.00000 Contribution: 22.81 %
Orbital 59 of fragment 1, Occ: 0.00000 Contribution: 2.18 %
Orbital 60 of fragment 1, Occ: 0.00000 Contribution: 1.66 %
Sum of values shown above: 97.83 %

Occupation number of orbital 154 of the complex: 0.00000000
Orbital 11 of fragment 2, Occ: 0.00000 Contribution: 2.01 %

Orbital 87 of fragment 3, Occ: 0.00000 Contribution: 77.45 %
Orbital 88 of fragment 3, Occ: 0.00000 Contribution: 9.52 %
Orbital 93 of fragment 3, Occ: 0.00000 Contribution: 1.88 %
Orbital 56 of fragment 1, Occ: 0.00000 Contribution: 5.67 %
Sum of values shown above: 96.51 %

Occupation number of orbital 155 of the complex: 0.00000000
Orbital 11 of fragment 2, Occ: 0.00000 Contribution: 2.74 %
Orbital 13 of fragment 2, Occ: 0.00000 Contribution: 1.57 %
Orbital 84 of fragment 3, Occ: 0.00000 Contribution: 1.19 %
Orbital 87 of fragment 3, Occ: 0.00000 Contribution: 7.17 %
Orbital 48 of fragment 1, Occ: 0.00000 Contribution: 1.15 %
Orbital 54 of fragment 1, Occ: 0.00000 Contribution: 2.18 %
Orbital 56 of fragment 1, Occ: 0.00000 Contribution: 80.11 %
Sum of values shown above: 96.09 %

Occupation number of orbital 156 of the complex: 0.00000000
Orbital 10 of fragment 2, Occ: 0.00000 Contribution: 2.32 %
Orbital 12 of fragment 2, Occ: 0.00000 Contribution: 37.30 %
Orbital 21 of fragment 2, Occ: 0.00000 Contribution: 1.54 %
Orbital 83 of fragment 3, Occ: 0.00000 Contribution: 22.35 %
Orbital 85 of fragment 3, Occ: 0.00000 Contribution: 4.94 %
Orbital 89 of fragment 3, Occ: 0.00000 Contribution: 16.54 %
Orbital 92 of fragment 3, Occ: 0.00000 Contribution: 1.47 %
Orbital 95 of fragment 3, Occ: 0.00000 Contribution: 5.12 %
Orbital 96 of fragment 3, Occ: 0.00000 Contribution: 6.40 %
Orbital 53 of fragment 1, Occ: 0.00000 Contribution: 1.61 %
Orbital 55 of fragment 1, Occ: 0.00000 Contribution: 1.58 %
Orbital 59 of fragment 1, Occ: 0.00000 Contribution: -2.31 %
Sum of values shown above: 98.84 %

Occupation number of orbital 157 of the complex: 0.00000000
Orbital 10 of fragment 2, Occ: 0.00000 Contribution: 5.80 %
Orbital 83 of fragment 3, Occ: 0.00000 Contribution: 4.64 %
Orbital 85 of fragment 3, Occ: 0.00000 Contribution: 2.83 %
Orbital 89 of fragment 3, Occ: 0.00000 Contribution: 5.78 %
Orbital 91 of fragment 3, Occ: 0.00000 Contribution: 9.40 %
Orbital 92 of fragment 3, Occ: 0.00000 Contribution: 23.40 %
Orbital 53 of fragment 1, Occ: 0.00000 Contribution: 1.64 %
Orbital 55 of fragment 1, Occ: 0.00000 Contribution: 38.61 %
Orbital 59 of fragment 1, Occ: 0.00000 Contribution: 2.12 %

Orbital 60 of fragment 1, Occ: 0.00000 Contribution: 1.27 %
Sum of values shown above: 95.48 %

Occupation number of orbital 158 of the complex: 0.00000000
Orbital 90 of fragment 3, Occ: 0.00000 Contribution: 76.24 %
Orbital 93 of fragment 3, Occ: 0.00000 Contribution: 1.38 %
Orbital 94 of fragment 3, Occ: 0.00000 Contribution: 3.17 %
Orbital 97 of fragment 3, Occ: 0.00000 Contribution: 2.69 %
Orbital 54 of fragment 1, Occ: 0.00000 Contribution: 2.40 %
Orbital 57 of fragment 1, Occ: 0.00000 Contribution: 9.26 %
Sum of values shown above: 95.14 %

Occupation number of orbital 159 of the complex: 0.00000000
Orbital 11 of fragment 2, Occ: 0.00000 Contribution: 2.47 %
Orbital 90 of fragment 3, Occ: 0.00000 Contribution: 12.97 %
Orbital 52 of fragment 1, Occ: 0.00000 Contribution: 2.49 %
Orbital 54 of fragment 1, Occ: 0.00000 Contribution: 5.86 %
Orbital 57 of fragment 1, Occ: 0.00000 Contribution: 73.93 %
Sum of values shown above: 97.73 %

Occupation number of orbital 160 of the complex: 0.00000000
Orbital 10 of fragment 2, Occ: 0.00000 Contribution: 7.56 %
Orbital 68 of fragment 3, Occ: 2.00000 Contribution: 1.37 %
Orbital 85 of fragment 3, Occ: 0.00000 Contribution: 7.72 %
Orbital 89 of fragment 3, Occ: 0.00000 Contribution: 1.87 %
Orbital 91 of fragment 3, Occ: 0.00000 Contribution: 3.76 %
Orbital 92 of fragment 3, Occ: 0.00000 Contribution: 55.45 %
Orbital 42 of fragment 1, Occ: 2.00000 Contribution: 1.16 %
Orbital 55 of fragment 1, Occ: 0.00000 Contribution: 8.40 %
Orbital 58 of fragment 1, Occ: 0.00000 Contribution: 1.24 %
Orbital 60 of fragment 1, Occ: 0.00000 Contribution: 4.12 %
Sum of values shown above: 92.65 %

Occupation number of orbital 161 of the complex: 0.00000000
Orbital 82 of fragment 3, Occ: 0.00000 Contribution: 1.28 %
Orbital 88 of fragment 3, Occ: 0.00000 Contribution: 9.75 %
Orbital 90 of fragment 3, Occ: 0.00000 Contribution: 2.20 %
Orbital 93 of fragment 3, Occ: 0.00000 Contribution: 76.70 %
Orbital 94 of fragment 3, Occ: 0.00000 Contribution: 2.55 %
Orbital 54 of fragment 1, Occ: 0.00000 Contribution: 1.35 %

Orbital 57 of fragment 1, Occ: 0.00000 Contribution: 1.98 %
Sum of values shown above: 95.81 %

Occupation number of orbital 162 of the complex: 0.00000000
Orbital 8 of fragment 2, Occ: 2.00000 Contribution: 1.26 %
Orbital 10 of fragment 2, Occ: 0.00000 Contribution: 6.59 %
Orbital 14 of fragment 2, Occ: 0.00000 Contribution: 10.65 %
Orbital 19 of fragment 2, Occ: 0.00000 Contribution: 2.76 %
Orbital 65 of fragment 3, Occ: 2.00000 Contribution: 1.43 %
Orbital 68 of fragment 3, Occ: 2.00000 Contribution: 1.44 %
Orbital 91 of fragment 3, Occ: 0.00000 Contribution: 8.44 %
Orbital 92 of fragment 3, Occ: 0.00000 Contribution: 1.29 %
Orbital 95 of fragment 3, Occ: 0.00000 Contribution: 7.37 %
Orbital 96 of fragment 3, Occ: 0.00000 Contribution: 1.70 %
Orbital 99 of fragment 3, Occ: 0.00000 Contribution: 1.76 %
Orbital 100 of fragment 3, Occ: 0.00000 Contribution: 1.10 %
Orbital 101 of fragment 3, Occ: 0.00000 Contribution: 1.39 %
Orbital 110 of fragment 3, Occ: 0.00000 Contribution: 1.88 %
Orbital 130 of fragment 3, Occ: 0.00000 Contribution: 1.45 %
Orbital 42 of fragment 1, Occ: 2.00000 Contribution: 1.24 %
Orbital 58 of fragment 1, Occ: 0.00000 Contribution: 36.57 %
Orbital 59 of fragment 1, Occ: 0.00000 Contribution: 1.40 %
Orbital 60 of fragment 1, Occ: 0.00000 Contribution: 2.88 %
Sum of values shown above: 92.61 %

Occupation number of orbital 163 of the complex: 0.00000000
Orbital 10 of fragment 2, Occ: 0.00000 Contribution: 4.79 %
Orbital 14 of fragment 2, Occ: 0.00000 Contribution: 4.86 %
Orbital 68 of fragment 3, Occ: 2.00000 Contribution: 1.48 %
Orbital 91 of fragment 3, Occ: 0.00000 Contribution: 9.78 %
Orbital 92 of fragment 3, Occ: 0.00000 Contribution: 4.06 %
Orbital 95 of fragment 3, Occ: 0.00000 Contribution: 3.08 %
Orbital 58 of fragment 1, Occ: 0.00000 Contribution: 60.88 %
Orbital 59 of fragment 1, Occ: 0.00000 Contribution: 1.13 %
Orbital 60 of fragment 1, Occ: 0.00000 Contribution: 1.28 %
Sum of values shown above: 91.33 %

Occupation number of orbital 164 of the complex: 0.00000000
Orbital 10 of fragment 2, Occ: 0.00000 Contribution: -5.35 %
Orbital 14 of fragment 2, Occ: 0.00000 Contribution: 16.88 %
Orbital 16 of fragment 2, Occ: 0.00000 Contribution: -1.17 %

Orbital 68 of fragment 3, Occ: 2.00000 Contribution: -1.17 %
Orbital 83 of fragment 3, Occ: 0.00000 Contribution: 2.99 %
Orbital 86 of fragment 3, Occ: 0.00000 Contribution: 1.56 %
Orbital 91 of fragment 3, Occ: 0.00000 Contribution: 52.75 %
Orbital 95 of fragment 3, Occ: 0.00000 Contribution: 7.88 %
Orbital 96 of fragment 3, Occ: 0.00000 Contribution: 3.95 %
Orbital 101 of fragment 3, Occ: 0.00000 Contribution: 2.82 %
Orbital 53 of fragment 1, Occ: 0.00000 Contribution: 2.47 %
Orbital 55 of fragment 1, Occ: 0.00000 Contribution: 9.04 %
Sum of values shown above: 92.65 %

Occupation number of orbital 165 of the complex: 0.00000000
Orbital 11 of fragment 2, Occ: 0.00000 Contribution: 13.64 %
Orbital 13 of fragment 2, Occ: 0.00000 Contribution: 3.88 %
Orbital 20 of fragment 2, Occ: 0.00000 Contribution: 1.40 %
Orbital 90 of fragment 3, Occ: 0.00000 Contribution: 1.04 %
Orbital 93 of fragment 3, Occ: 0.00000 Contribution: 3.87 %
Orbital 94 of fragment 3, Occ: 0.00000 Contribution: 7.06 %
Orbital 104 of fragment 3, Occ: 0.00000 Contribution: 1.28 %
Orbital 50 of fragment 1, Occ: 0.00000 Contribution: 2.67 %
Orbital 52 of fragment 1, Occ: 0.00000 Contribution: 12.40 %
Orbital 54 of fragment 1, Occ: 0.00000 Contribution: 34.66 %
Orbital 57 of fragment 1, Occ: 0.00000 Contribution: 13.11 %
Orbital 61 of fragment 1, Occ: 0.00000 Contribution: 3.11 %
Sum of values shown above: 98.12 %

Occupation number of orbital 166 of the complex: 0.00000000
Orbital 11 of fragment 2, Occ: 0.00000 Contribution: 2.09 %
Orbital 15 of fragment 2, Occ: 0.00000 Contribution: 1.83 %
Orbital 84 of fragment 3, Occ: 0.00000 Contribution: 1.48 %
Orbital 90 of fragment 3, Occ: 0.00000 Contribution: 3.15 %
Orbital 93 of fragment 3, Occ: 0.00000 Contribution: 1.19 %
Orbital 94 of fragment 3, Occ: 0.00000 Contribution: 71.22 %
Orbital 97 of fragment 3, Occ: 0.00000 Contribution: 4.35 %
Orbital 52 of fragment 1, Occ: 0.00000 Contribution: 2.40 %
Orbital 54 of fragment 1, Occ: 0.00000 Contribution: 5.06 %
Orbital 63 of fragment 1, Occ: 0.00000 Contribution: 1.90 %
Sum of values shown above: 94.66 %

Occupation number of orbital 167 of the complex: 0.00000000
Orbital 10 of fragment 2, Occ: 0.00000 Contribution: -2.00 %

Orbital 14 of fragment 2, Occ: 0.00000 Contribution: 8.22 %
Orbital 91 of fragment 3, Occ: 0.00000 Contribution: 3.13 %
Orbital 95 of fragment 3, Occ: 0.00000 Contribution: 36.02 %
Orbital 96 of fragment 3, Occ: 0.00000 Contribution: 21.20 %
Orbital 99 of fragment 3, Occ: 0.00000 Contribution: 1.46 %
Orbital 100 of fragment 3, Occ: 0.00000 Contribution: 15.61 %
Orbital 101 of fragment 3, Occ: 0.00000 Contribution: 3.33 %
Orbital 59 of fragment 1, Occ: 0.00000 Contribution: 2.36 %
Orbital 62 of fragment 1, Occ: 0.00000 Contribution: 1.26 %
Sum of values shown above: 90.60 %

Occupation number of orbital 168 of the complex: 0.00000000
Orbital 10 of fragment 2, Occ: 0.00000 Contribution: 2.00 %
Orbital 12 of fragment 2, Occ: 0.00000 Contribution: -1.63 %
Orbital 14 of fragment 2, Occ: 0.00000 Contribution: -2.61 %
Orbital 95 of fragment 3, Occ: 0.00000 Contribution: 3.99 %
Orbital 96 of fragment 3, Occ: 0.00000 Contribution: 1.13 %
Orbital 100 of fragment 3, Occ: 0.00000 Contribution: 2.32 %
Orbital 51 of fragment 1, Occ: 0.00000 Contribution: 1.26 %
Orbital 53 of fragment 1, Occ: 0.00000 Contribution: 1.49 %
Orbital 55 of fragment 1, Occ: 0.00000 Contribution: 2.21 %
Orbital 59 of fragment 1, Occ: 0.00000 Contribution: 48.94 %
Orbital 60 of fragment 1, Occ: 0.00000 Contribution: 19.44 %
Orbital 62 of fragment 1, Occ: 0.00000 Contribution: 7.17 %
Orbital 65 of fragment 1, Occ: 0.00000 Contribution: 9.58 %
Sum of values shown above: 95.30 %

Occupation number of orbital 169 of the complex: 0.00000000
Orbital 90 of fragment 3, Occ: 0.00000 Contribution: 1.68 %
Orbital 94 of fragment 3, Occ: 0.00000 Contribution: 4.47 %
Orbital 97 of fragment 3, Occ: 0.00000 Contribution: 18.59 %
Orbital 98 of fragment 3, Occ: 0.00000 Contribution: 70.19 %
Orbital 102 of fragment 3, Occ: 0.00000 Contribution: 3.04 %
Sum of values shown above: 97.97 %

Occupation number of orbital 170 of the complex: 0.00000000
Orbital 97 of fragment 3, Occ: 0.00000 Contribution: 49.14 %
Orbital 98 of fragment 3, Occ: 0.00000 Contribution: 16.69 %
Orbital 108 of fragment 3, Occ: 0.00000 Contribution: 1.11 %
Orbital 61 of fragment 1, Occ: 0.00000 Contribution: 25.61 %
Orbital 63 of fragment 1, Occ: 0.00000 Contribution: 1.62 %

Sum of values shown above: 94.17 %

Occupation number of orbital 171 of the complex: 0.00000000
Orbital 94 of fragment 3, Occ: 0.00000 Contribution: 3.22 %
Orbital 97 of fragment 3, Occ: 0.00000 Contribution: 18.08 %
Orbital 98 of fragment 3, Occ: 0.00000 Contribution: 7.15 %
Orbital 54 of fragment 1, Occ: 0.00000 Contribution: 1.11 %
Orbital 61 of fragment 1, Occ: 0.00000 Contribution: 59.26 %
Orbital 63 of fragment 1, Occ: 0.00000 Contribution: 2.24 %
Orbital 66 of fragment 1, Occ: 0.00000 Contribution: 3.10 %
Orbital 67 of fragment 1, Occ: 0.00000 Contribution: 1.29 %
Sum of values shown above: 95.45 %

Occupation number of orbital 172 of the complex: 0.00000000
Orbital 10 of fragment 2, Occ: 0.00000 Contribution: 1.54 %
Orbital 12 of fragment 2, Occ: 0.00000 Contribution: 1.05 %
Orbital 95 of fragment 3, Occ: 0.00000 Contribution: 2.56 %
Orbital 99 of fragment 3, Occ: 0.00000 Contribution: 2.51 %
Orbital 100 of fragment 3, Occ: 0.00000 Contribution: 3.33 %
Orbital 60 of fragment 1, Occ: 0.00000 Contribution: 30.32 %
Orbital 62 of fragment 1, Occ: 0.00000 Contribution: 52.28 %
Sum of values shown above: 93.60 %

Occupation number of orbital 173 of the complex: 0.00000000
Orbital 12 of fragment 2, Occ: 0.00000 Contribution: 5.26 %
Orbital 14 of fragment 2, Occ: 0.00000 Contribution: 2.03 %
Orbital 89 of fragment 3, Occ: 0.00000 Contribution: 2.12 %
Orbital 95 of fragment 3, Occ: 0.00000 Contribution: 13.22 %
Orbital 96 of fragment 3, Occ: 0.00000 Contribution: 11.76 %
Orbital 99 of fragment 3, Occ: 0.00000 Contribution: 44.68 %
Orbital 101 of fragment 3, Occ: 0.00000 Contribution: 2.31 %
Orbital 105 of fragment 3, Occ: 0.00000 Contribution: 2.82 %
Orbital 107 of fragment 3, Occ: 0.00000 Contribution: 1.58 %
Orbital 109 of fragment 3, Occ: 0.00000 Contribution: 1.57 %
Orbital 60 of fragment 1, Occ: 0.00000 Contribution: 1.79 %
Orbital 62 of fragment 1, Occ: 0.00000 Contribution: 5.22 %
Orbital 64 of fragment 1, Occ: 0.00000 Contribution: 2.05 %
Sum of values shown above: 96.40 %

Occupation number of orbital 174 of the complex: 0.00000000

Orbital 95 of fragment 3, Occ: 0.00000 Contribution: 6.32 %
Orbital 96 of fragment 3, Occ: 0.00000 Contribution: 2.84 %
Orbital 99 of fragment 3, Occ: 0.00000 Contribution: 2.84 %
Orbital 100 of fragment 3, Occ: 0.00000 Contribution: 57.56 %
Orbital 101 of fragment 3, Occ: 0.00000 Contribution: 16.64 %
Orbital 105 of fragment 3, Occ: 0.00000 Contribution: 3.25 %
Orbital 62 of fragment 1, Occ: 0.00000 Contribution: 1.80 %
Orbital 64 of fragment 1, Occ: 0.00000 Contribution: 1.79 %
Sum of values shown above: 93.04 %

Occupation number of orbital 175 of the complex: 0.00000000
Orbital 15 of fragment 2, Occ: 0.00000 Contribution: 6.46 %
Orbital 18 of fragment 2, Occ: 0.00000 Contribution: 2.05 %
Orbital 102 of fragment 3, Occ: 0.00000 Contribution: 2.46 %
Orbital 103 of fragment 3, Occ: 0.00000 Contribution: 7.50 %
Orbital 108 of fragment 3, Occ: 0.00000 Contribution: 1.46 %
Orbital 61 of fragment 1, Occ: 0.00000 Contribution: 4.25 %
Orbital 63 of fragment 1, Occ: 0.00000 Contribution: 59.90 %
Orbital 67 of fragment 1, Occ: 0.00000 Contribution: 5.83 %
Sum of values shown above: 89.90 %

Occupation number of orbital 176 of the complex: 0.00000000
Orbital 10 of fragment 2, Occ: 0.00000 Contribution: 3.98 %
Orbital 12 of fragment 2, Occ: 0.00000 Contribution: 1.66 %
Orbital 14 of fragment 2, Occ: 0.00000 Contribution: 4.80 %
Orbital 83 of fragment 3, Occ: 0.00000 Contribution: 1.03 %
Orbital 95 of fragment 3, Occ: 0.00000 Contribution: 1.73 %
Orbital 99 of fragment 3, Occ: 0.00000 Contribution: 15.37 %
Orbital 100 of fragment 3, Occ: 0.00000 Contribution: 5.45 %
Orbital 101 of fragment 3, Occ: 0.00000 Contribution: 6.00 %
Orbital 107 of fragment 3, Occ: 0.00000 Contribution: 8.23 %
Orbital 110 of fragment 3, Occ: 0.00000 Contribution: 4.54 %
Orbital 112 of fragment 3, Occ: 0.00000 Contribution: 1.87 %
Orbital 130 of fragment 3, Occ: 0.00000 Contribution: 1.71 %
Orbital 59 of fragment 1, Occ: 0.00000 Contribution: 3.51 %
Orbital 60 of fragment 1, Occ: 0.00000 Contribution: 6.99 %
Orbital 62 of fragment 1, Occ: 0.00000 Contribution: 16.96 %
Orbital 64 of fragment 1, Occ: 0.00000 Contribution: 1.11 %
Orbital 65 of fragment 1, Occ: 0.00000 Contribution: 7.62 %
Sum of values shown above: 92.55 %

Occupation number of orbital 177 of the complex: 0.00000000

Orbital	83 of fragment	3, Occ: 0.00000	Contribution: 2.28 %
Orbital	95 of fragment	3, Occ: 0.00000	Contribution: 6.48 %
Orbital	96 of fragment	3, Occ: 0.00000	Contribution: 3.62 %
Orbital	99 of fragment	3, Occ: 0.00000	Contribution: 11.54 %
Orbital	100 of fragment	3, Occ: 0.00000	Contribution: 2.53 %
Orbital	101 of fragment	3, Occ: 0.00000	Contribution: 19.98 %
Orbital	107 of fragment	3, Occ: 0.00000	Contribution: 1.90 %
Orbital	112 of fragment	3, Occ: 0.00000	Contribution: 1.98 %
Orbital	59 of fragment	1, Occ: 0.00000	Contribution: 2.26 %
Orbital	60 of fragment	1, Occ: 0.00000	Contribution: 12.05 %
Orbital	62 of fragment	1, Occ: 0.00000	Contribution: 4.79 %
Orbital	64 of fragment	1, Occ: 0.00000	Contribution: 1.66 %
Orbital	65 of fragment	1, Occ: 0.00000	Contribution: 20.94 %

Sum of values shown above: 92.00 %

Occupation number of orbital 178 of the complex: 0.00000000

Orbital	97 of fragment	3, Occ: 0.00000	Contribution: 2.38 %
Orbital	102 of fragment	3, Occ: 0.00000	Contribution: 58.40 %
Orbital	103 of fragment	3, Occ: 0.00000	Contribution: 26.40 %
Orbital	104 of fragment	3, Occ: 0.00000	Contribution: 1.15 %
Orbital	108 of fragment	3, Occ: 0.00000	Contribution: 2.18 %
Orbital	63 of fragment	1, Occ: 0.00000	Contribution: 2.26 %
Orbital	67 of fragment	1, Occ: 0.00000	Contribution: 2.32 %

Sum of values shown above: 95.10 %

Occupation number of orbital 179 of the complex: 0.00000000

Orbital	12 of fragment	2, Occ: 0.00000	Contribution: -1.18 %
Orbital	21 of fragment	2, Occ: 0.00000	Contribution: 1.76 %
Orbital	91 of fragment	3, Occ: 0.00000	Contribution: 1.01 %
Orbital	99 of fragment	3, Occ: 0.00000	Contribution: 2.32 %
Orbital	100 of fragment	3, Occ: 0.00000	Contribution: 1.03 %
Orbital	101 of fragment	3, Occ: 0.00000	Contribution: 10.99 %
Orbital	105 of fragment	3, Occ: 0.00000	Contribution: 13.55 %
Orbital	107 of fragment	3, Occ: 0.00000	Contribution: 5.97 %
Orbital	109 of fragment	3, Occ: 0.00000	Contribution: 3.52 %
Orbital	110 of fragment	3, Occ: 0.00000	Contribution: 12.29 %
Orbital	60 of fragment	1, Occ: 0.00000	Contribution: 1.56 %
Orbital	62 of fragment	1, Occ: 0.00000	Contribution: 1.70 %
Orbital	64 of fragment	1, Occ: 0.00000	Contribution: 35.44 %
Orbital	65 of fragment	1, Occ: 0.00000	Contribution: 3.34 %

Sum of values shown above: 93.30 %

Occupation number of orbital 180 of the complex: 0.00000000

Orbital	11 of fragment	2, Occ: 0.00000	Contribution: 1.15 %
Orbital	15 of fragment	2, Occ: 0.00000	Contribution: 4.79 %
Orbital	94 of fragment	3, Occ: 0.00000	Contribution: 1.11 %
Orbital	102 of fragment	3, Occ: 0.00000	Contribution: 19.43 %
Orbital	103 of fragment	3, Occ: 0.00000	Contribution: 14.39 %
Orbital	104 of fragment	3, Occ: 0.00000	Contribution: 21.27 %
Orbital	108 of fragment	3, Occ: 0.00000	Contribution: 4.51 %
Orbital	61 of fragment	1, Occ: 0.00000	Contribution: 1.48 %
Orbital	63 of fragment	1, Occ: 0.00000	Contribution: 14.73 %
Orbital	66 of fragment	1, Occ: 0.00000	Contribution: 6.09 %
Orbital	67 of fragment	1, Occ: 0.00000	Contribution: 1.19 %
Orbital	68 of fragment	1, Occ: 0.00000	Contribution: 3.21 %
Orbital	70 of fragment	1, Occ: 0.00000	Contribution: 1.26 %

Sum of values shown above: 94.62 %
